# The effects of thioamide backbone substitution on protein stability: a study in α-helical, β-sheet, and polyproline II helical contexts†
†Electronic supplementary information (ESI) available: Syntheses of peptides and proteins, characterization, data fitting, supplementary CD spectra, and other experimental details. See DOI: 10.1039/c6sc05580j



**DOI:** 10.1039/c6sc05580j

**Published:** 2017-02-08

**Authors:** Christopher R. Walters, D. Miklos Szantai-Kis, Yitao Zhang, Zachary E. Reinert, W. Seth Horne, David M. Chenoweth, E. James Petersson

**Affiliations:** a Department of Chemistry , University of Pennsylvania , 231 S. 34th Street , Philadelphia , PA 19104 , USA; b Biochemistry and Molecular Biophysics Graduate Group , University of Pennsylvania , 3700 Hamilton Walk , Philadelphia , PA 19104 , USA; c Department of Chemistry , University of Pittsburgh , 219 Parkman Avenue , Pittsburgh , PA 15260 , USA

## Abstract

Thioamides are single atom substitutions of the peptide bond that serve as versatile probes of protein structure. Effective use of thioamides requires a robust understanding of the impact that the substitution has on a protein of interest. However, the thermodynamic effects of thioamide incorporation have only been studied in small structural motifs, and their influence on secondary structure in the context of full-length proteins is not known. Here we describe a comprehensive survey of thioamide substitutions in three benchmark protein systems (calmodulin, the B1 domain of protein G, and collagen) featuring the most prevalent secondary structure motifs: α-helix, β-sheet, and polyproline type II helix. We find that in most cases, effects on thermostability can be understood in terms of the positioning and local environment of the thioamide relative to proximal structural elements and hydrogen bonding networks. These observations set the stage for the rational design of thioamide substituted proteins with predictable stabilities.

## Abstract 2

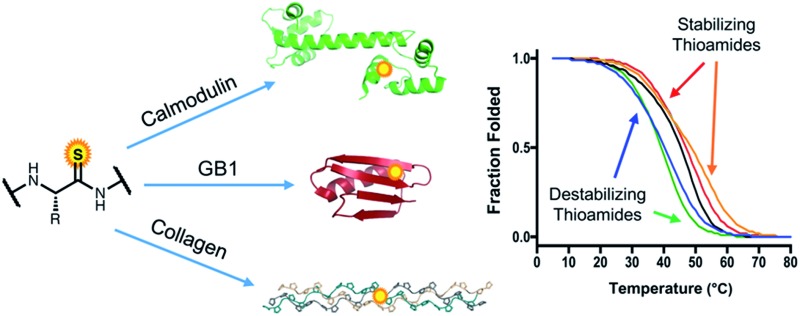
Thioamides are single atom substitutions of the peptide bond that serve as versatile probes of protein structure.

## Introduction

The incorporation of functional molecules for monitoring and manipulating protein folding has greatly facilitated studies of biochemical phenomena.^1^ The optimal choice of modification to a protein of interest depends on the type of process to be tracked, disrupted, strengthened, or isolated. Recently, there has been an emerging interest in making subtle alterations to the protein backbone to provide additional means to probe and mimic dynamic protein movements and interactions.^2–6^ In particular, substituting a thioamide peptide linkage at single or multiple positions provides a small but powerful means to influence and/or monitor protein dynamics or function.

Thioamides (a.k.a. thionoamides, thioxoamides) have distinct properties from oxoamides, including a larger van der Waals radius, red shifted π → π* and n → π* transitions, lower oxidation potential, lower infrared (IR) stretching frequency and lower N–H p*K*_a_.^7–11^ These properties have allowed thioamides to be leveraged as photoswitches, Förster resonance energy transfer (FRET) acceptors, and photoinduced electron transfer quenchers, as well as IR and circular dichroism (CD) spectroscopic probes in biochemical systems.^12–21^ Although thioamides have already proven to be valuable multifaceted probes, few studies have sought to elucidate the energetic impacts they have on peptide or protein folding. This consideration is of the utmost importance in assessing the thioamide's potential as a “minimalist” spectroscopic label.

Previous structural studies of thioamide substitutions largely relied upon short, isolated protein motifs in the absence of a tertiary structure context. In fact, when we began this work, there were no published studies of thioamides in proteins of defined tertiary structure. Fischer, Kiefhaber, and coworkers provided the most complete study of the effects of thioamides in α-helices, using model helical polyalanine peptides bearing thioalanine (Ala^S^, thioamide substitutions are denoted by superscript “S” throughout) substitutions.^19^ Their analysis revealed that the thioamide disrupts the helix if substituted at a central position, but is less destabilizing at the N-terminus. Their results suggest that the thioamide is similar to a glycine substitution at the same position in terms of helix disruption. Thioamides have also been incorporated into the 15-*mer* S peptide, which forms an α-helix upon binding to ribonuclease S.^22^ Substitutions were made throughout the helix and were destabilizing at every position, with varying ΔΔ*G* values in comparison to the WT complex (0.6–4.7 kcal mol^–1^). However, in a different study, Miwa and coworkers observed that a Leu^S^ substitution in the central region of an α-helical coiled-coil dimer gave a similarly helical structure to the oxoamide counterpart (as observed by CD) and increased the melting temperature by 10 °C.^17^ This discrepancy, which likely arises from subtle geometric differences between an isolated α-helix and that in a coiled coil,^23^ implies that tertiary structure may serve to lessen or even reverse destabilization by a thioamide.

In an alternate secondary structure context, the thioamide was found to be tolerated between residues *i* + 2 and *i* + 3 in a type II′ β-turn.^18^ Here, the thiocarbonyl is solvent-exposed, and the interior of the turn does not require any reorganization to accommodate the larger sulfur atom. Thioamide substitutions have been made in a tryptophan-rich β-hairpin to interrogate the role of hydrogen bond formation in the folding transition state.^12^ However, the results of this study may not be general to all β-sheets due to the highly engineered sequence of the tryptophan zipper peptide. Most recently, Raines and coworkers made thioamide substitutions in collagen model peptides (CMPs) to assess their impact in the Pro-Pro-Gly (PPG) repeats of an all PPG polyproline type II (PPII) helix.^24^ It was found that incorporation of Gly^S^ (*i.e.*, PPG^S^) was significantly destabilizing, whereas substitution with Pro^S^ (*i.e.*, PP^S^G) was slightly stabilizing to the triple helix.

Taken together, the above studies present a limited understanding about where thioamides can be utilized most efficiently in biological systems. Thioamides are likely to have much more nuanced effects in proteins with complex tertiary folds and dynamic regions. Thus far, only three full-length thioamide proteins have been described in the literature: the semi-synthetic constructs α-synuclein and dihydrofolate reductase, and the natural protein methyl-coenzyme M reductase.^25–27^ Consequently, there have been no systematic studies of thioamides in folded proteins, which will be essential to guiding their future use as spectroscopic probes or as modulators of protein structure and function.

Herein, we describe an in-depth study of the effects of thioamide backbone substitution in three benchmark protein systems: (1) the C-terminal loop and helix of calmodulin (CaM), an α-helical protein; (2) the β-sheet of the B1 domain of protein G (GB1), a compact α/β tertiary structure; and (3) the PPII helix of a Pro-Hyp-Gly (POG) based CMP. For each protein, we performed CD spectroscopy studies to elucidate the structural and thermodynamic stability changes resulting from thioamide insertion. The first two systems represent new hosts for examination of the effects of thioamides on protein secondary structure in a tertiary fold context. In CMPs, our results build on prior published work through complete positional scanning substitutions in both PPG and POG subunits. Overall, the findings reported here lay the groundwork for the rational implementation of thioamides as biophysical probes in diverse protein systems.

## Results and discussion

### Design and semi-synthesis of CaM thioproteins

CaM is a 148 amino acid, α-helical calcium signaling protein that is ubiquitous in eukaryotes. It is comprised of two structurally similar domains (N- and C-terminal), each containing two calcium binding sites.^28^ Upon binding calcium, CaM undergoes a conformational change to expose a *trans*-domain helix, which acts as a binding platform for many of its regulatory target proteins.^29,30^ Previously, a semi-synthesis of CaM was performed to modify the C-terminal EF-hand.^31^ Since these authors observed some destabilization of CaM, we decided to limit thioamide substitutions in our investigations to the C-terminal loop and helix, near the highest affinity calcium binding site. Thus, we performed native chemical ligation (NCL) reactions between the expressed fragment CaM_1–134_ and the synthetic fragment CaM_135–148_.

**Fig. 1:**
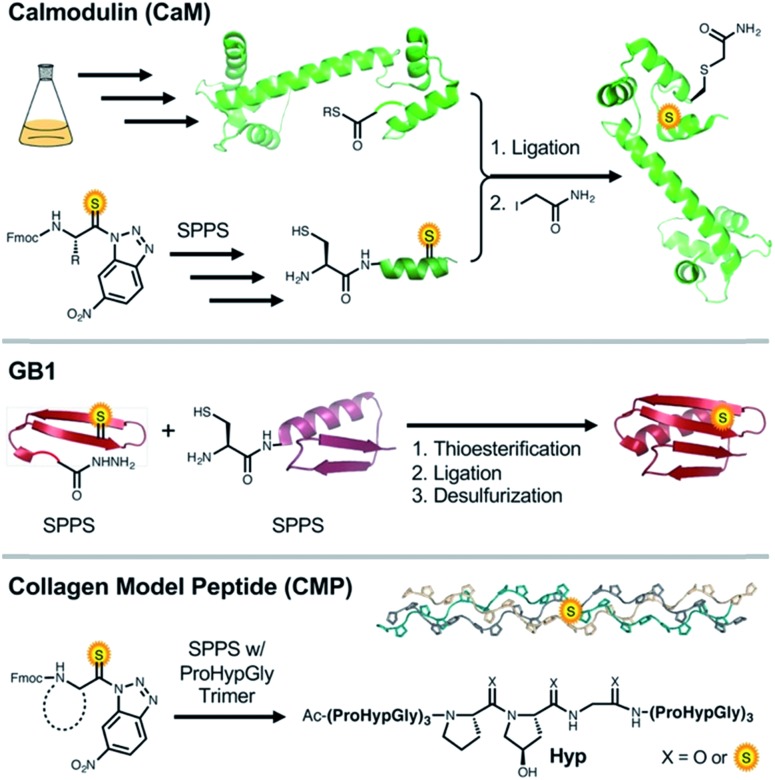
Top: NCL synthesis of CaM thioproteins. Cys_135_ is masked as glutamine by alkylation with iodoacetamide. Middle: synthesis of GB1 thioproteins by hydrazide ligation. Cys_24_ undergoes desulfurization to the native residue Ala_24_. Bottom: total solid phase synthesis of collagen strands by POG trimer and thioamide precursor couplings. Upon completion of synthesis, triple helices are formed by incubation of the monomers at 4 °C.

An existing CaM intein fusion was modified to produce CaM_1–134_-GyrA-His_6_, which was expressed, purified, and cleaved with 2-mercaptoethanesulfonate (MES) to yield the C-terminal thioester CaM_1–134_-MES (Fig. 1, Top).^32^ Each thiopeptide was made by solid phase peptide synthesis (SPPS) with the thioamide installed as previously described.^33^ NCL reactions were performed between the CaM_135–148_ fragment and CaM_1–134_-MES using published conditions optimized for thiopeptides.^25^ The native residue at position 135 in CaM is a Gln. Therefore, Cys at this position was masked after NCL to mimic Gln (denoted as Cys^Q^) by iodoacetamide treatment. This process routinely yielded 1–2 mg quantities of purified CaM (8–25% isolated yield with CaM_1–134_-MES protein as a limiting reagent).

**Fig. 2:**
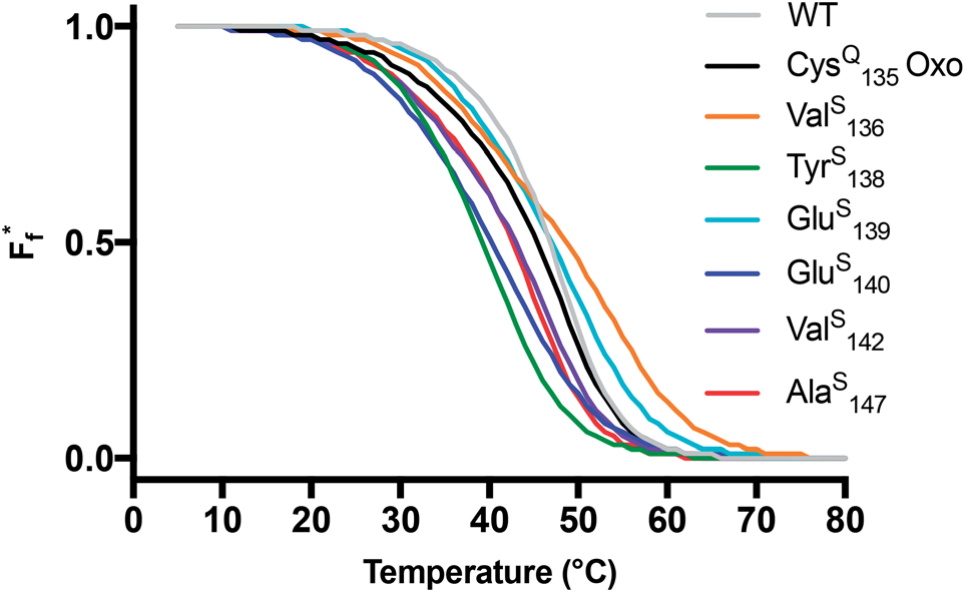
Thermal stability of CaM thioamide variants. CD thermal melts of stabilizing and destabilizing apo CaM variants plotted as fraction folded 
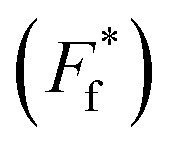
 fits showing a weighted contribution of the intermediate to the folded state as discussed in the text. Melting curves were obtained by measuring the *θ*_222_ at 1 °C increments in 10 mM Tris, 0.5 mM EDTA pH 7.5. Transformation of raw CD signal to MRE values and three-state fitting procedures were performed as described in eqn (S1) and (S4)–(S6).† Data for additional mutants can be found in the ESI.†

**Fig. 3:**
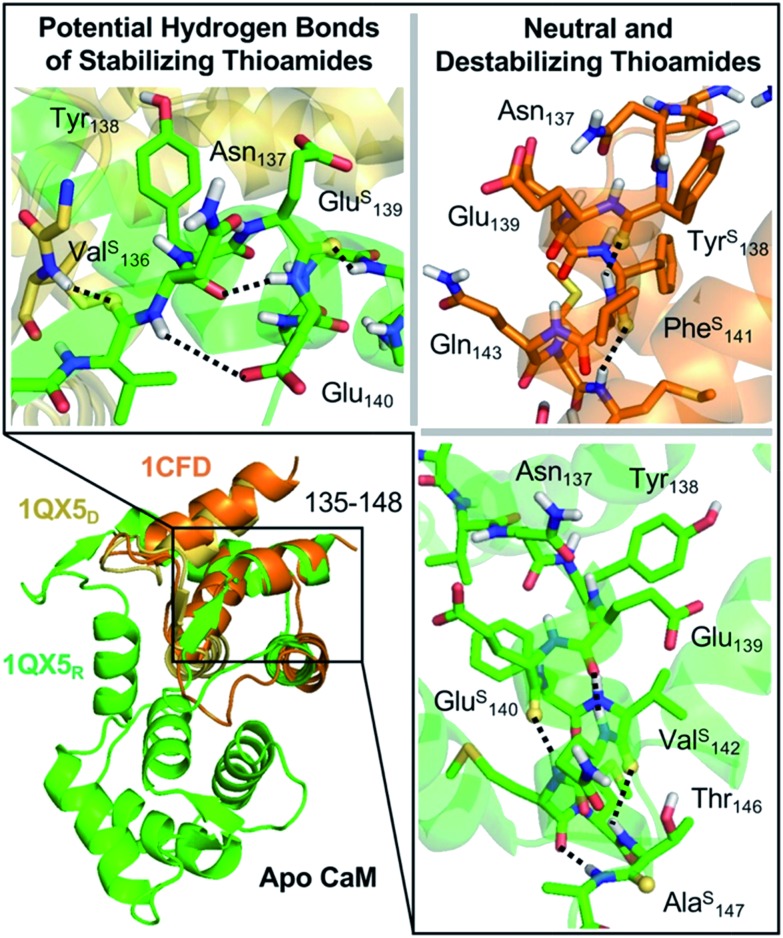
Structural analysis of CaM thioamide variants. Top Left: potential hydrogen bonding interactions for stabilization observed in Val^S^_136_ and Glu^S^_139_ substitutions. Right: neutral (Val^S^_142_ and Ala^S^_147_) and destabilizing (Tyr^S^_138_, Glu^S^_140_, and Phe^S^_141_) substitutions. Destabilization of CaM by Phe^S^_141_ and Tyr^S^_138_ and may be due to disruption in the packing of the helix. Glu^S^_140_ disrupts hydrogen bond acceptance without making a compensatory hydrogen bond donor interaction. Increased C=O···N distance (3.1 Å) in the turn of the helix at Val^S^_142_ may allow for better accommodation of the thioamide sulfur. Ala^S^_147_ is the penultimate C-terminal residue and only serves as a hydrogen bond donor. Structures rendered from PDB entry ; 1QX5 chains D (yellow) and R (green) and from PDB entry ; 1CFD (orange, only N-terminal region shown).^34,35^

Positions for thioamide substitutions were selected with three criteria in mind: importance to local secondary structure, functional importance for Ca^2+^ binding, and ease of synthesis of peptides and thioamide precursors. Of particular interest were residues in the C-terminal helix of Ca^2+^-bound CaM (Tyr_138_, Glu_139_, Glu_140_, Phe_141_, and Val_142_; Fig. 2 and 3). Glu_140_ is functionally important as it undergoes significant conformational change from the apo protein in order to directly chelate a Ca^2+^ ion in the holo protein.^29^ Tyr_138_, Glu_139_, Phe_141_, and Val_142_ reside in the N-terminal and central portions of the helix. We hypothesized that thioamide substitution at these sites would be destabilizing due to the weaker hydrogen bond acceptor capacity of the thiocarbonyl. Two additional sites were chosen to assess the impact of substitutions in loops and solvent exposed areas proximal to this helix (Val_136_ and Ala_147_, the penultimate residue in CaM).

Proteins bearing each of these thioamide substitutions were synthesized using the MES thioester method described above and verified by MALDI-MS (Table S3†). Wild-type (WT) CaM and a Cys_135_ mutant were expressed in *E. coli* as controls for proper refolding of the thioproteins. The Cys_135_ mutant was subjected to denaturing NCL conditions similar to the thioproteins and subsequently underwent the same purification and capping reaction.

### CaM thioprotein folding thermodynamics

Each CaM variant was characterized by CD spectroscopy under two different buffer conditions: one for the calcium-bound holo protein (10 mM Tris pH 7.5, 2 mM CaCl_2_) and another for the calcium-free apo form (10 mM Tris pH 7.5, 0.5 mM ethylenediaminetetraacetic acid, EDTA). CaM CD spectra show a prototypical α-helical signature with minima at 208 and 222 nm (Fig. S5†). Spectra for the thioprotein variants include a small minimum between 260 and 280 nm resulting from the thioamide π → π* transition. This peak is weaker than the comparable transition observed in GB1 and collagen (*vide infra*), or in previous reported small thiopeptides.^17–19^ The attenuation of the thiocarbonyl CD signal is likely an environmental and concentration based effect, as the thioamide in CaM is a small fraction of the overall amide content and the region containing the thioamide is destabilized in several variants. The thioamide n → π* transition at 340 nm is not visible in any of the CaM CD spectra.

Among the holo proteins, the spectrum of Cys^Q^_135_ is similar in shape and magnitude to that of WT CaM, indicating the side chain modification necessary to enable NCL has no significant effect on the fold (Fig. S5,† Top). Most of the thioamide substitutions appear well tolerated in the holo protein; however, the Tyr^S^_138_ and Phe^S^_141_ modifications considerably alter the folded state, with a nearly three-fold decrease in molar residue ellipticity (MRE, [*θ*]) at 222 and 208 nm. These mutants show a similar helical signature to the N-terminal truncation mutant CaM_1–71_, suggesting that the C-terminal domain may be completely disordered as a result of thioamide incorporation (Fig. S6†). To understand more about the nature of each thioamide substitution, we subjected the CaM variants to thermal denaturation. Like WT CaM, each of the thioamide variants and the Cys^Q^_135_ oxo control have melting temperatures greater than 80 °C in the presence of Ca^2+^ (Fig. S7†).

Upon the removal of calcium by EDTA, CaM reverts to the apo structure, which lacks the stable transdomain helix and open EF hands observed in the holo form.^34,35^ We hypothesized that the substantial structural differences between the apo and holo folds may lead a thioamide at a given site to have distinct effects in those two contexts. Thus, we carried out an analogous series of CD experiments to characterize folding in the apo CaM variants. Surprisingly, the Cys^Q^_135_ modification appears to be significantly perturbing to the apo form, reducing helicity by half relative to apo WT CaM (Fig. S5,† Bottom). While the origins of the effect of side chain modification at residue 135 are not clear, we used the data observed for the Cys^Q^_135_ oxo control as a benchmark to probe the impact of thioamide incorporation on the apo fold.

Each of the apo proteins show complete unfolding transitions over the temperature range of 5° to 95 °C. Initial apo thermal melts were performed on WT CaM and the Cys^Q^_135_ control. The melting temperature for our WT CaM sample is lower than values previously reported in literature,^36–39^ likely due to different buffer conditions. Although we attempted to fit our data to a two-state model (Table S7, Fig. S8 and S9†), we ultimately chose to analyze our results using a three-state model as it is well established that CaM unfolding proceeds through a semi-stable intermediate.^39^ We therefore report thermodynamic data for each transition (*T*_M1_, Δ*H*_1_, *T*_M2_, and Δ*H*_2_) and an overall Δ*G*_U_ that is the sum of the individual unfolding free energies for each transition calculated at 25 °C (Table 1).

**Table 1:** Apo CaM thermodynamic values

CaM variant	*T*_M1_ ^*a*^ (°C)	Δ*H*_1_ ^*a*^ (kcal mol^–1^)	*T*_M2_ ^*b*^ (°C)	Δ*H*_2_ ^*b*^ (kcal mol^–1^)	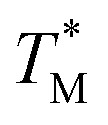 ^*c*^ (°C)	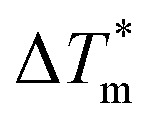 (°C)	Δ*G*_U_ ^*d*^ (kcal mol^–1^)	ΔΔ*G*_U_ (kcal mol^–1^)
WT	43.5 ± 0.6	33.1 ± 0.8	48.6 ± 0.6	60.5 ± 0.7	46.8 ± 0.3	—	6.4 ± 0.2	—
Cys^Q^_135_ Oxo	38.9 ± 0.8	30.2 ± 0.9	48.8 ± 0.7	61.7 ± 0.8	45.5 ± 0.4	—	5.9 ± 0.1	—
Val^S^_136_	39.6 ± 0.5	36.7 ± 0.6	55.3 ± 0.8	52.0 ± 0.5	48.6 ± 0.7	3.2	6.5 ± 0.1	0.6
Tyr^S^_138_	34.7 ± 0.8	39.7 ± 0.7	43.0 ± 0.9	51.1 ± 1.0	39.3 ± 0.8	–6.2	4.1 ± 0.2	–1.8
Glu^S^_139_	40.7 ± 0.7	40.1 ± 0.4	51.9 ± 0.6	53.9 ± 0.9	47.0 ± 0.1	1.6	6.4 ± 0.1	0.5
Glu^S^_140_	33.9 ± 1.1	34.5 ± 0.6	45.5 ± 0.8	45.9 ± 1.0	40.4 ± 0.8	–5.1	3.9 ± 0.2	–2.0
Val^S^_142_	36.4 ± 0.6	35.5 ± 0.8	48.4 ± 0.8	62.9 ± 0.9	44.0 ± 0.7	–1.5	5.8 ± 0.2	–0.1
Ala^S^_147_	37.2 ± 0.9	32.1 ± 0.2	47.0 ± 0.6	63.2 ± 1.3	43.7 ± 0.7	–1.8	5.5 ± 0.2	–0.3

^*a*^Values obtained from the global three state fit for the first transition (nominally C-terminal unfolding).

^*b*^Values obtained from the global three state fit for the second transition (nominally N-terminal unfolding).

^*c*^Melting temperature weighted for contributions from the unfolding of each domain.

^*d*^Δ*G*_U_ calculated from *T*_M1_, Δ*H*_1_, *T*_M2_, and Δ*H*_2_ using eqn (S4)–(S7) as described in the ESI.

For comparison of three-state CaM unfolding to the two-state unfolding of GB1 and CMPs, we generated pseudo fraction folded 
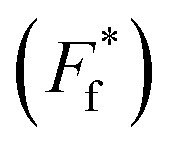
 plots based on this three-state model, where the intermediate state makes a weighted contribution to the total fraction of folded protein (Fig. 2). We denote the half-point of these weighted unfolding curves as 
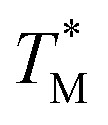
. Using this fitting model, we determined that Cys^Q^_135_ CaM is slightly less stable than WT CaM in 
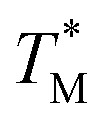
 and Δ*G*_U_; however, this minor change in stability should not prevent it from serving as an appropriate control to study the effects of the thioamide substitutions.

Using the Cys^Q^_135_ protein as a basis for comparison, we see that Ala^S^_147_ slightly destabilizes the apo fold, lowering Δ*G*_U_(25) by 0.3 kcal mol^–1^. In agreement with the CD spectra, Tyr^S^_138_ and Glu^S^_140_ appear to be the most destabilizing mutations, decreasing Δ*G*_U_ by 1.8 and 2.0 kcal mol^–1^, respectively. Val^S^_142_ is well-tolerated, giving rise to a Δ*G*_U_ within error of the oxoamide. Unexpectedly, both Val^S^_136_ and Glu^S^_139_ show elevated melting temperatures and an increase of Δ*G*_U_ by ∼0.5 kcal mol^–1^. The unstable Phe^S^_141_ construct was prone to aggregation during thermal denaturation, precluding rigorous thermodynamic analysis. Nevertheless, the thioamide substitutions that we could study span a range of thermodynamic effects, which we can interpret in terms of known structures.

The analysis of the thioamide substitutions in apo CaM is limited by the small number of high-resolution structures available. Here, our analysis will focus on structures of the apo protein from *Rattus norvegicus* (crystal, PDB entry ; 1QX5) and *Xenopus laevis* (NMR, PDB entry ; 1CFD).^34,35^ While the differences in these two structures are valuable in considering the dynamics of apo CaM, it should be noted that crystal packing in the ; 1QX5 structure gives rise to non-native intermolecular interactions. However, these are analogous to intramolecular interactions observed in the NMR structure (Fig. 3 and described in detail in Fig. S14†). As detailed below, both structures provide valuable insight into the effects of the thioamide substitutions.

We analyze the destabilizing substitutions first. Based on comparison to the Cys^Q^_135_ control, Ala^S^_147_ is mildly destabilizing. This is somewhat surprising as the thioamide in question is located at the C-terminal end of the helix and therefore should serve as an N–H hydrogen bond donor with the thiocarbonyl sulfur oriented into solvent (Fig. 3 and S16†). Val^S^_142_ shows only a minimal reduction in *T*_M_ (Fig. 3). In both the NMR and X-ray structure, the C=O···N distance for the hydrogen bond that the Val^S^_142_ thiocarbonyl would make with Thr_146_ is somewhat long (3.1 Å and 3.3 Å, respectively), so the thiocarbonyl could reasonably be accommodated there (Fig. S16†). Placing a thioamide near the start of the C-terminal helix (Tyr^S^_138_ or Glu^S^_140_) is highly destabilizing, presumably due to disruption of backbone hydrogen bonding. Both these residues have relatively short C=O···N hydrogen bond distances and also interact with neighboring loops or helices (Fig. 3 Right, Fig. S16–S17 and Table S8†).

Two thioamide variants were significantly stabilized relative to the oxoamide: Val^S^_136_ and Glu^S^_139_. In both cases, the stabilization appears to arise from a combination of a location sterically tolerant to the thioamide carbonyl and a strengthening of hydrogen bonds involving the thioamide N–H. In the ; 1CFD structure, the C=O···N distance for the hydrogen bond that the Val^S^_136_ thiocarbonyl would make with Ile_100_ is 3.4 Å and the C=O···N angle is 137°. In the ; 1QX5 structure, this carbonyl makes an intermolecular hydrogen bond with another CaM molecule in the crystal lattice (Fig. 3 Left, Fig. S15, and Table S8†). Thus, the longer thiocarbonyl could certainly be accommodated in this flexible region of apo CaM and might even make for a more favorable interaction. In the ; 1QX5 structure, the Glu^S^_139_ carbonyl C=O···N distance from the Val_142_/Gln_143_ amide N–H is 3.0 Å and the C=O···N angle is 144°, making it seemingly unfavorable for thioamide substitution. However, in the ; 1CFD NMR structure, this distance and angle are 3.4 Å and 145°, respectively, indicating sufficient flexibility in this region to accommodate the thiocarbonyl (Fig. S16 and Table S8†).

For both Val^S^_136_ and Glu^S^_139_, there is also a potential to acquire a new favorable thioamide N–H interaction. In the ; 1QX5 structure, the Glu_140_ N–H (part of a thioamide in Glu^S^_139_) is positioned ideally in the plane of the backbone carbonyl of Asn_137_ with a 3.3 Å N···O=C hydrogen bond length (Fig. 3 Left). Alternatively, in the ; 1CFD structure, the Glu^S^_139_ thioamide N–H makes a hydrogen bond with the sidechain carbonyl of Asn_137_ (Fig. S16†). Although this region of CaM is dynamic, both structures offer plausible explanations for the increased stability of the Glu^S^_139_ variant. On the other hand, for Val^S^_136_, the explanation is less clear-cut. In the ; 1QX5 structure, the distance from the side chain carbonyl of Glu_140_ to the backbone nitrogen of Asn_137_ is 4.2 Å and the carbonyl is out of plane from the N–H bond (Fig. S15†). No interaction of this N–H with Glu_140_ is apparent in the ; 1CFD structure. Nonetheless, the side chain of Glu_140_ appears to be highly mobile (average B-factor of 86.5 Å^2^ in ; 1QX5) and reorientation of the side chain to favor this hydrogen bond may be possible. Although previously unappreciated, these interactions may be responsible for the positioning of the C-terminal helix for correct folding of the helix-loop-helix motif in the EF-hand. However, it is impossible to know the exact details of how each thioamide is changing the local and distal dynamics of the protein until further high-resolution structural studies are performed.

### Design and synthesis of GB1 thioproteins

The immunoglobulin-binding B1 domain of protein G from *Streptococcus* bacteria is 56 amino acids in length and has a tertiary structure comprised of three of the most common secondary structural motifs in proteins.^40^ The compact GB1 fold consists of a protein-spanning α-helix packed against a four-stranded β-sheet with both parallel and anti-parallel strands, making it an ideal model to study local folding dynamics in a tertiary structure context. Indeed, NMR, X-ray crystallography, and an array of computational studies have been used to great effect in understanding the order in which individual secondary structure elements fold to form the final tertiary structure of GB1.^41,42^ More recently, various unnatural backbone substitutions have been examined in GB1.^43–46^ While these substitutions were thermodynamically destabilizing to the folded state, many of the proteins showed a native-like fold by X-ray crystallography and CD. Given these precedents, GB1 is an excellent platform to examine effects of thioamides on folding in a β-sheet, a structural motif not yet studied in detail with thioamide substitutions.

GB1 and three thioamide variants were synthesized using a combination of automated and manual SPPS, followed by NCL. We originally intended to synthesize thioamide GB1 through SPPS only, but encountered low yields due to suboptimal coupling reactions after thioamide insertion and Edman degradation-type cleavage of the amide bond C-terminal to the thioamide under acidic deprotection conditions. Thioprotein production by NCL allowed us to work with shorter thiopeptide fragments where the number of couplings subsequent to thioamide insertion and the length of the deprotection reaction can be limited. In the synthesis of GB1 and variants, we performed a ligation between a thioamide containing GB1_1–23_ thioester and GB1_24–56_Cys_24_. After desulfurization of Cys_24_ using VA-044 and sacrificial thioacetamide, we obtained the thioamide GB1 constructs with no trace of the ligation point and yields ranging between 10 and 20% (Fig. 1, Middle).^47^

Using this NCL and desulfurization strategy, we synthesized three GB1 variants with single thioamide substitutions at Leu_5_, Ile_6_, or Leu_7_. The Leu_5_ residue acts as a hydrogen bond acceptor in an anti-parallel β-sheet interaction and as a hydrogen bond donor in a parallel β-sheet interaction. In contrast, Ile_6_ acts as a hydrogen bond acceptor in a parallel β-sheet interaction and as a hydrogen bond donor in an anti-parallel β-sheet interaction. The hydrogen bonding pattern of Leu_7_ is similar to that of Leu_5_ (anti-parallel acceptor, parallel donor), but the carbonyl points outward slightly, increasing the hydrogen bond distance by 0.2 Å and placing it out of plane with respect to its partner in the sheet. Thus, we anticipated that the increased size and C=X bond length of the thiocarbonyl might be better accommodated at Leu_7_ than at Leu_5_ or Ile_6_. These three substitutions, while not comprehensive, provide examples of how thioamides may be accommodated into both types of β-sheet folds within the context of a complex tertiary domain.

### GB1 thioprotein folding thermodynamics

GB1 CD spectra typically contain a broad minimum between 208 and 222 nm with few defined features in this range due to the combination of the α-helical and β-sheet contributions to the signal. The Leu^S^_5_ and Ile^S^_6_ substitutions have nearly identical spectra and maintain similar curve features as the GB1 Oxo control, but with significantly reduced MRE values (Fig. S19†). It is likely that the internal hydrogen bonding networks are perturbed by these substitutions, leading to a disruption in the packing of the tertiary structure. Leu^S^_7_ seems to be the most tolerated thioamide substitution with a CD signature closest in magnitude to the control at 208 nm. However, the MRE values are still significantly reduced across the entire set of spectra.

**Fig. 4:**
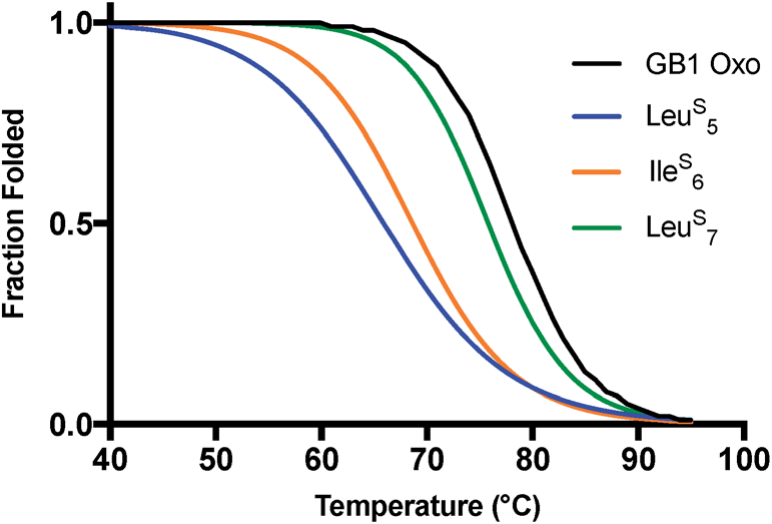
Thermal stability of GB1 thioamide variants. CD thermal melts of GB1 variants plotted as fraction folded values generated from a two-state fitting model. Melting curves were obtained by measuring the *θ*_220_ at 1 °C increments in 20 mM Na_2_HPO_4_ pH 7.0. Transformation of raw CD signal to MRE values and two-state fitting procedures were performed as described in eqn (S1)–(S3).†

For GB1, the thermal denaturation curves (Fig. 4) fit well to a two-state unfolding model. The *T*_M_ of GB1 Oxo is 78.2 °C and includes a sharp unfolding transition that is preceded by only a modest initial unfolding event occurring gradually between 5 and 65 °C (Fig. S20 and S21†). The thermodynamic differences for each thioamide substitution track with the changes in signal observed in the wavelength scans. Ile^S^_6_ leads to a 9.5 °C destabilization in melting temperature while Leu^S^_5_ leads to an even greater destabilization of 12.6 °C. Leu^S^_7_ is the least destabilizing thioamide substitution with a melting temperature only 2.3 °C lower than WT GB1. The corresponding unfolding free energies (ΔΔ*G*_U_ calculated according to eqn (S8), see ESI†) show that Leu^S^_7_ is destabilized by only 0.5 kcal mol^–1^. Thus, we see that the precise context of thioamide substitution is also important for β-sheet systems.

**Fig. 5:**
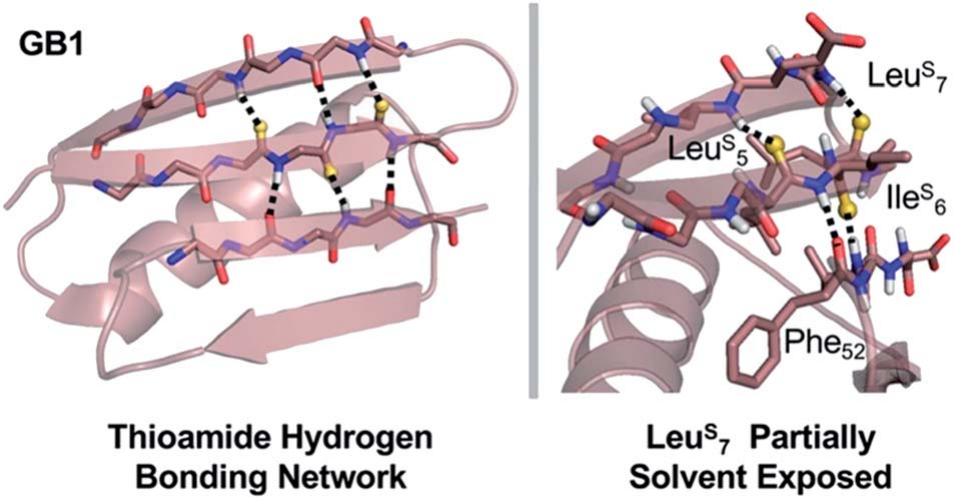
Structural analysis of GB1 thioamide variants. Left: Overall structure of GB1 with modeled thioamide substitutions. Each thioamide substitution is poised to make two hydrogen bonding interactions with opposing strands. Right: Zoomed in region showing that the Leu^S^_7_ thiocarbonyl is skewed out of the plane of the anti-parallel β-sheet (C=O···N angle = 134°), providing a potential explanation for why it is less destabilizing than Leu^S^_5_ or Ile^S^_6_. Structures rendered from PDB entry ; 2QMT.^48^

In GB1, the thermodynamic and structural changes from thioamide incorporation can be readily rationalized based on existing structural data (PDB entry 2QMT).^48^ Leu^S^_5_ thiocarbonyl substitution likely disrupts the hydrogen bond with the N–H of Thr_16_, altering packing of only the outer strand of the β-sheet (Fig. 5). The Leu^S^_5_ thioamide N–H should maintain the hydrogen bond to the Phe_52_ carbonyl in the core parallel β-sheet interface and might even strengthen this interaction. Computational modeling indicates that Leu_5_ forms part of the folding nucleus of GB1,^41^ so perhaps it is not surprising that the Leu_5_ substitution has the most significant effect on the *T*_M_ and reduced the cooperativity of the folding transition. Ile^S^_6_ reverses this hydrogen bonding pattern, maintaining the N–H hydrogen bond to the carbonyl of Gly_14_ in the outer strand and disrupting hydrogen bonding with the Val_54_ amide N–H in the core strand. Disturbance of the core of the β-sheet region would explain why the Ile^S^_6_ substitution induces such a significant change in the *T*_M_. Leu^S^_7_ causes almost no disruption in folding as determined by CD. It seems that the longer hydrogen bond distance (3.1 Å to the Lys_13_/Gly_14_ amide N) and out-of-plane orientation of the Leu_7_ carbonyl are permissive of the sulfur substitution, as we anticipated. If there is any minor disturbance, it could be compensated for by the increased strength of the Leu^S^_7_ thioamide N–H hydrogen bond with the carbonyl of Val_54_. Taken together, these analyses reinforce our observation that the effects of thioamide incorporation in β-sheets can depend dramatically on the location of the substitution (Table 2).

**Table 2:** GB1 thermodynamic values

GB1 variant	*T*_m_ (°C)	Δ*T*_M_ (°C)	ΔΔ*G*_U_ ^*a*^ (kcal mol^–1^)
Oxo	78.2 ± 0.0	—	—
Leu^S^_5_	65.6 ± 1.6	–12.6	–2.5 ± 0.3
Ile^S^_6_	68.7 ± 0.5	–9.5	–1.9 ± 0.1
Leu^S^_7_	75.9 ± 1.2	–2.3	–0.5 ± 0.2

^*a*^Change in unfolding free energy, ΔΔ*G*_U_, calculated from Δ*T*_M_ using eqn (S8) as described in the ESI.

### Design and synthesis of collagen model thiopeptides

Collagen provides an opportunity to explore the thioamide's potential as a modulator of key stabilizing hydrogen bonds and n → π* interactions. The protein is comprised of three monomeric left-handed PPII helices that are made up of Xaa-Yaa-Gly repeats. These monomers anneal to one another to form the native structure, a right-handed triple helix.^49^ Although, ProProGly (PPG) is one of the most prevalent repeat elements in collagen, nearly every other amino acid has been observed in the Xaa or Yaa position.^50^ Within this PPG unit, a variety of substitutions have been made on the 4 position of the pyrrolidine ring.^51–56^ The most common alteration found physiologically is (2*S*,4*R*)-4-hydroxyproline at the Yaa position (Pro-Hyp-Gly, POG).^50^ Backbone ester, alkene, and aza-glycine substitutions have also been incorporated to modify and study the PPII triple helix.^57–59^ Recently Raines *et al.* reported the effects of thioamide incorporation in two positions (ProPro^S^Gly and ProProGly^S^) by thermal denaturation in a PPG based CMP.^24^ Here, we build on this precedent by providing detailed kinetics and thermodynamics for single substitutions in all Xaa, Yaa, and Gly positions in a 21 *mer* POG host peptide system.

To probe the effect of the thioamide on CMP self-assembly, we installed the moiety near the central positions of the 21-*mer* host system Ac-(POG)_3_(XYG)(POG)_3_-NH_2_. Peptides were synthesized through SPPS, coupling Fmoc-POG-OH trimers at all but the central POG subunit. Here, individual residues were installed, including the suitable building block for incorporation of the thioamide. Typical yields of completed thioamide CMPs range between 4–6%. To analyze the thioamide impact on structure, thermodynamics, and kinetics of folding, we subjected each CMP variant to CD scans, thermal melts, and kinetic refolding experiments.

### Collagen model thiopeptide folding thermodynamics

**Fig. 6:**
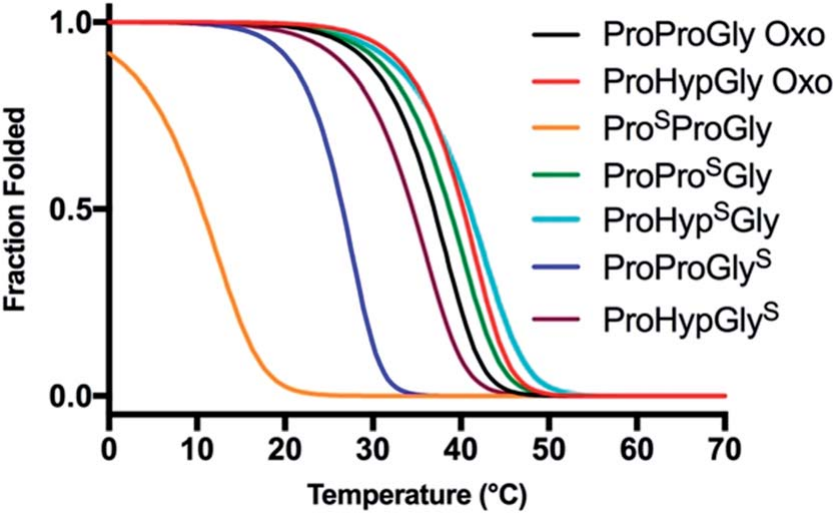
Thermal stability of CMP thioamide variants. CD thermal melts of collagen plotted as fraction folded (*F*_f_) values generated from the fit. Melting curves were obtained by measuring the *θ*_224_ at 1 °C increments in PBS buffer. Transformation of raw CD signal to MRE values is described in eqn (S1)† and fitting procedures were as previously described.^52,60,61^

**Fig. 7:**
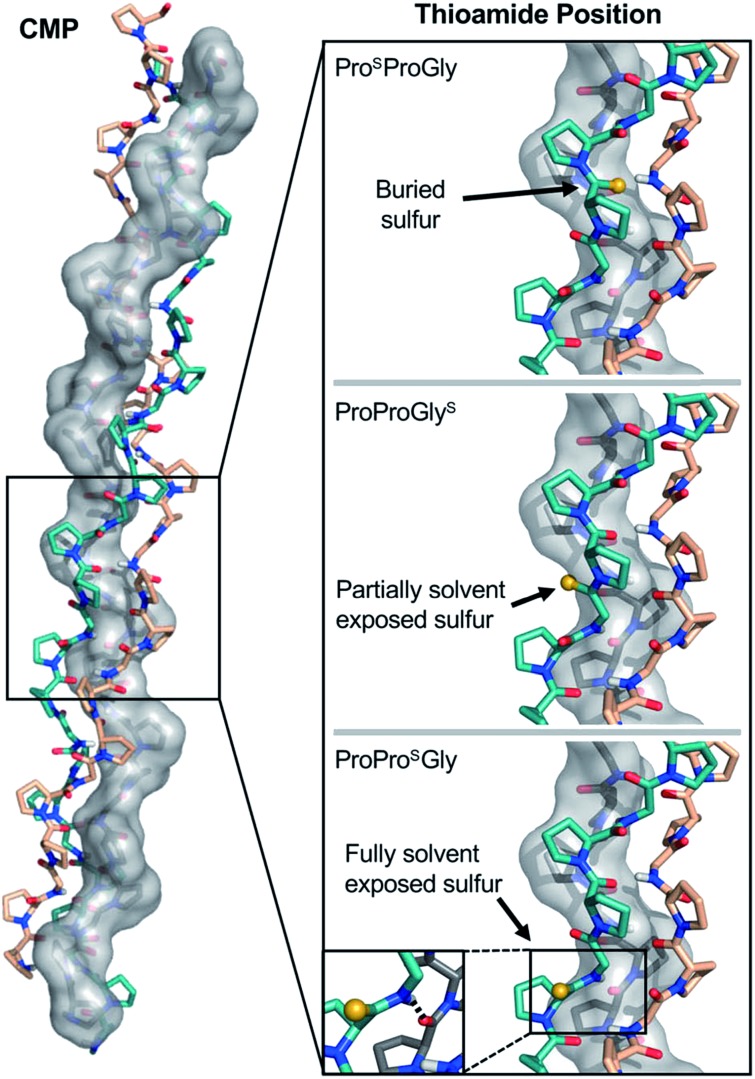
Structural analysis of CMP thioamide variants. Pro^S^ProGly and ProProGly^S^ have buried and partially buried sulfur atoms, respectively (Top and Middle), accounting for the disruptions observed in the CD experiments. The ProPro^S^Gly (Bottom) sulfur atom is fully solvent exposed and the N–H of the thioamide forms a hydrogen bond with the strand shown in grey (inset), accounting for the stabilization observed in the CD experiments. Structures rendered from PDB entry ; 2CUO.^67^

CD scans of each thioamide variant display a characteristic collagen spectrum with a minimum at 198 nm and maximum around 225 nm, along with a broad minimum centered around 265 nm, representing the π → π* contribution from the thioamide. The magnitude of the minima at 198 nm and 265 nm depend upon the position of the thioamide, with the P^S^PG substitution leading to the greatest loss in MRE signal (Fig. S27–S31†). Unsurprisingly, the P^S^PG replacement massively destabilizes the protein (Fig. 6 and Table 3), likely because the sulfur points inward towards the core of the trimer, imposing a steric obstacle to the packing of the strands (Fig. 7). Additionally, the lower electronegativity of the elongated carbon–sulfur bond weakens key interchain H-bonding between the glycine N–H and the C=O of the Xaa proline. The PP^S^G modification shows a stabilization of the triple helix with a significant increase in *T*_m_ and 0.4 kcal mol^–1^ increase in Δ*G*_U_, while PPG^S^ shows a moderate destabilization, in agreement with previous studies.^24^ Hysteresis studies showed that the free energy differences are in good agreement with the melting temperature profiles (Fig. S32–S36†). Additionally, each thioamide CMP folded on a similar timescale to the PPG control, demonstrating that the kinetics of folding are not altered by thioamide substitution.

**Table 3:** CMP thermodynamic and kinetic values

Collagen variant	*T*_M_ (°C)	Δ*G*_U_ ^*a*^	ΔΔ*G*_U_ ^*a*^	*t*_1/2_ (min)
Pro-Pro-Gly Oxo	36.7 ± 0.4	10.8		32.5 ± 2.0
Pro^S^-Pro-Gly	11.0 ± 0.9	5.1	–5.7	25.0 ± 4.5
Pro-Pro^S-^Gly	38.5 ± 0.5	11.2	0.4	35.3 ± 5.2
Pro-Pro-Gly^S^	30.3 ± 0.5	9.2	–1.6	40.3 ± 7.6
Pro-Hyp-Gly Oxo	39.9 ± 0.5	12.0		24.0 ± 4.5
Pro-Hyp^S^-Gly	41.0 ± 0.2	12.6	0.6	17.7 ± 4.0
Pro-Hyp-Gly^S^	34.5 ± 0.0	11.1	–0.9	23.8 ± 2.8

^*a*^Unfolding free energy in kcal mol^–1^, calculated as described in the ESI.

To assess thioamide backbone compatibility with substitutions at the 4 position of the pyrrolidine ring in proline, we incorporated Hyp into the Yaa position. This POG mutant is well known to stabilize collagen triple helices through stereoelectronic effects and hydrogen bonding.^62–66^ Results show that peptides PO^S^G and POG^S^ exhibit *T*_M_ increases of 2.5 and 4.2 °C, respectively, relative to their corresponding Pro congeners. Interestingly, the stabilizing effects from the Hyp substitution and thioamide appear additive when compared to appropriate PPG and POG controls. Thioamide substitution at the central Yaa position contributes about 0.5 kcal mol^–1^ of stability and Hyp substitution contributes about 1.3 kcal mol^–1^. Furthermore, the PO^S^G substitution leads to faster folding compared to that of any thioamide variant or control peptide examined here. Like for CaM and GB1, thioamide substitutions in collagen have effects that strongly depend on the position of incorporation. Further combinations of thioamide and pyrrolidine modifications may also garner additive gains in collagen stability, or even act to compensate for other modifications previously observed to be destructive.

Analysis of thioamide substitutions in PPG and POG CMPs illustrates the three fundamental effects that thioamides are expected to have on proteins. In PP^S^G, the combination of weaker hydrogen bonding and steric clashes due to the larger van der Waals radius of sulfur and longer C=S bond length significantly destabilizes (Δ*T*_M_ = –25.7 °C) the protein (Fig. 7). In contrast, both PP^S^G and PO^S^G collagen peptides are moderately stabilized compared to their oxoamide counterparts. At the Yaa position, the thioamide N–H can form a stabilizing interstrand hydrogen bond, while the thiocarbonyl projects into solvent. Thus, thioamide substitution at the Yaa position confers the energetic benefits of the stronger hydrogen bond donor without the penalties of the weaker acceptor or steric clashes. The disruption introduced by the PPG^S^ thioamide is not as drastic as the P^S^PG replacement because it does not pack against the core of the triple helix, but it may provide general steric interference with neighboring strand contacts. The PO^S^G and POG^S^ peptides recapitulate the positional effects of the thioamide while retaining the stabilizing effects of Hyp. The additivity of the backbone and sidechain substitutions supports the idea that the conformations of the thioamide collagen variants are similar to the parent collagen model peptides (with the exception of P^S^PG, for which few conclusions can be drawn). In this regard, our data show that a thioamide substitution can act synergistically with a proline ring substitution for potential applications in thiopeptide based collagen materials.

## Conclusions

Thioamides have the potential to be one of the most multifunctional probes in the large repertoire of unnatural amino acid substitutions available for protein labeling. They can be used as functional handles in IR, CD, or fluorescence spectroscopy, as photoswitches, or as perturbants in structure/function studies. With thioamide substitutions across three protein systems, CaM, GB1, and CMPs, the data set amassed here represents the most comprehensive study to date bearing on the question of how thioamides affect protein thermostability. While virtually all previous reports of thioamides in secondary structure contexts have found the modification to be disruptive, we show that, within CaM, the replacement is tolerated in some positions within the C-terminal α-helix as well as the preceding loop. While all thioamide substitutions in the β-sheet of GB1 were destabilizing, at one position the impact was minimal. Our CMP results are consistent with findings by Raines, and although we do not yet have high resolution structures, the additivity of the effects of thioamidation and proline hydroxylation suggests that our CMPs adopt a fold consistent with structures like PDB entry 2CUO, shown in Fig. 7.

Overall, our analysis based on existing crystal and NMR structures allows us to rationalize many of our findings in terms of the physical properties of the thioamide bond and to consider how both perturbing and non-perturbing thioamide locations might be useful. Thioamide incorporation in the first turn of an α-helix is a strong helix breaker, and it is disruptive in sheets where it replaces a short hydrogen bond that is in the plane of the sheet. These findings can be explained by the longer C=S bond length and larger van der Waals radius of sulfur, and highlight the potential of thioamides as tools to modulate protein folding. Thioamides will be tolerated in helices where the substituted amide oxygen participates in longer H-bonds and in sheets where the carbonyl bond is at a more acute angle. We have also seen that thioamides are tolerated, or even stabilizing, when the disruptive effect of the thiocarbonyl is compensated by the stronger hydrogen bond donation of the thioamide N–H. Minimally perturbing substitutions are ideal for fluorescence studies using the thioamide as a quenching moiety. Increased thermal stability of collagen PP^S^G and PO^S^G substitutions, as well as Val^S^_136_ and Glu^S^_139_ in CaM, highlight the fact that thioamides can serve as stabilizing backbone replacements for interrogating hydrogen bonding networks.

The results reported here have set a foundation for rational thioprotein design to realize the above benefits to biophysics and protein engineering. A growing database of thioamide “mutants” could, in conjunction with appropriate computational models, allow one to predict the effects of thioamide substitution to achieve the desired destabilizing, neutral, or stabilizing effects on a protein of interest. Efforts are underway in our laboratories to obtain high resolution structural and dynamic information on thioamide substitution. In addition to the generation of thioamide proteins by NCL, Hecht's recent co-translational incorporation of thioamide dipeptides using mutant ribosomes offers a potentially more facile semi-synthetic route to thioproteins.^26^ Deciphering the mechanism of incorporation of the natural thioglycine residue in the archael methyl-coenzyme M reductase could also permit the *in vivo* biosynthetic incorporation of backbone thioamides.^27^ As thioamide proteins become more synthetically accessible, the results reported here should provide valuable insights into the design of appropriate thioproteins for diverse applications.
