# Supplementary material for: The effects of thioamide backbone substitution on protein stability: a study in α-helical, β-sheet, and polyproline II helical contexts
Source: Chem Sci. 2017 Feb 8;8(4):2868–77. doi: 10.1039/c6sc05580j (PMC5428018; doi:10.1039/c6sc05580j)
Supplement: Supplementary file 1 [file SC-008-C6SC05580J-s001.pdf]

Electronic Supplementary Material (ESI) for Chemical Science.
This journal is © The Royal Society of Chemistry 2017

The Effects of Thioamide Backbone Substitution on Protein Stability: A study in α-helical, β-Sheet, and polyproline II helical contexts

Christopher R. Walters,[a] D. Miklos Szantai-Kis,[b] Yitao Zhang,[a] Zachary E. Reinert,[c] W. Seth Horne,[c] David M. Chenoweth,[a] and E. James Petersson[a,*]

[a]Department of Chemistry, University of Pennsylvania, 231 S. 34th Street, Philadelphia, PA, 19104

[b]Biochemistry and Molecular Biophysics Graduate Group, University of Pennsylvania, 3700 Hamilton Walk, Philadelphia, PA 19104

[c]Department of Chemistry, University of Pittsburgh, 219 Parkman Avenue, Pittsburgh, PA 15260

General Information. Fmoc protected amino acids and peptide coupling reagents as well as Rink amide and 2-chlorotrityl resins were purchased from EMD Millipore (Billerica, MA, USA) with the exception of Fmoc Hyp(tBu)-OH, which was purchased from Advanced Chemtech (Louisville, KY, USA). Hydrazide hydrate solution was purchased from Oakwood Chemical (Estill, SC, USA). Piperidine was purchased from American Bioanalytical (Natick, MA, USA). VA-044 was purchased from Wako Pure Chemical Industries (Osaka, Japan). Triisopropylsilane (TIPS) was purchased from Santa Cruz Biotechnology, Inc (Dallas, TX, USA). $GB1_{24\text{-}56}$ A24C was purchased from Genscript (Piscataway, NJ, USA). Restriction enzymes and chitin purification resin were purchased from New England Biolabs (Ipswich, MA, USA) and *Pfu* Turbo DNA Polymerase was purchased from Agilent Technologies (Santa Clara, CA, USA). DNA oligomers were purchased from Integrated DNA Technologies, Inc (Coralville, IA, USA). The pTXB1 plasmid containing $CaM_{S17C,\ 1\text{-}116}$ was a gift from the Linse Laboratory of the Department of Biochemistry and Structural Biology at Lund University. Isopropyl β-D-1-thiogalactopyranoside (IPTG) was purchased from LabScientific, Inc (Highlands, NJ, USA). Protease inhibitor tablets were purchased from Roche Boehringer Mannheim (Indianapolis, IN, USA). Amicon Ultra centrifugal filter units were purchased from EMD Millipore (Billerica, MA, USA). Sodium 2-mercaptoethanelsulfonate (MES•Na) was purchased from TCI America (Portland, OR, USA). β-mercaptoethanol (βME) was purchased from Bio-Rad Laboratories (Hercules, CA, USA). FPLC purification of proteins was performed on an AKTA system with HiTrap Q columns (GE Healthcare Bio-Sciences, Pittsburgh, PA, USA), while peptide HPLC was performed on a Varian Prostar system (currently Agilent Technologies, Santa Clara, CA, USA) for CaM and GB1 peptides and a Jasco HPLC instrument (Easton, MD, USA) for collagen. Thiopropyl sepharose 6b resin was also purchased from GE Healthcare (Princeton, NJ, USA). Cuvettes for CD spectroscopy were purchased from Hellma Analytics USA (Plainview, NY, USA). All other reagents, solvents, and materials were purchased from either Fisher Scientific (Pittsburgh, PA, USA) or Sigma-Aldrich (St. Louis, MO, USA) unless otherwise specified. Matrix assisted laser desorption/ionization mass spectrometry (MALDI) was performed on a Bruker Ultraflex III mass spectrometer (Billerica, MA). High Resolution Mass Spectrometry (HRMS) for small molecules were obtained on a Waters LCT Premier XE LC/MS system (Milford, MA, USA). Nuclear magnetic resonance spectra were obtained on a Bruker DRX 500 MHz instrument. Common abbreviations for all other chemicals are as follows: 1-[Bis(dimethylamino)methylene]-1*H*-1,2,3-

triazolo[4,5-b]pyridinium 3-oxid hexafluorophosphate (HATU), 2-(1H-benzotriazol-1-yl)-1,1,3,3-tetramethyluronium hexafluorophosphate (HBTU), *O*-(1H-6-Chlorobenzotriazole-1-yl)-1,1,3,3-tetramethyluronium hexafluorophosphate (HCTU), *N*-methylmorpholine (NMM), isobutyl chloroformate (ICBF), *N*,*N*-diisopropylethylamine (DIPEA), trifluoroacetic acid (TFA), 1,2-ethanedithiol (EDT), hydroxybenzotriazole (HOBt), 4-(2-hydroxyethyl)-1-piperazineethanesulfonic acid (HEPES), tris(2-carboxyethyl)phosphine (TCEP), and guanidinium hydrochloride (Gdn•HCl).

Chemical Synthesis of Thioacylating Monomers for Thiopeptides

The below route was adapted from Rapoport *et al.*[1] Abbreviations for reagents can be found in the general information section. The syntheses of Ala, Phe, Glu, Val, Pro, and Leu thioamide precursors have been reported previously.[2-7]

Scheme S1. Synthesis of Nitrobenzotriazolide Thioacylating Monomers

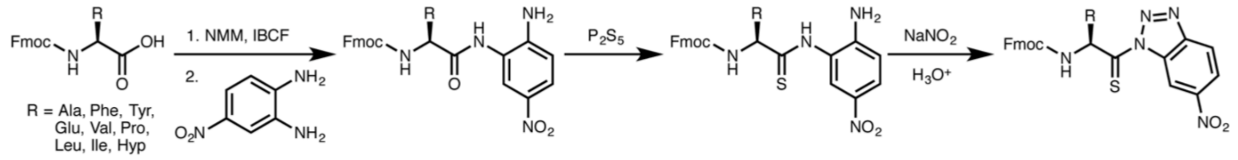

Synthesis and Purification of Thiotyrosine (Fmoc-Tyr^S-NBt) Precursor

(9*H*-fluoren-9-yl)methyl (*S*)-(1-((2-amino-5-nitrophenyl)amino)-3-(4-(*tert*-butoxy)phenyl)-1-oxopropan-2-yl)carbamate (1). Fmoc-Tyr(*t*-Bu)-OH (1.49 g, 3.25 mmol) was dissolved in 25 mL of tetrahydrofuran (THF) under argon flow with stirring. The solution was then cooled to -10 °C in a 1:3 NaCl/ice bath. To this solution, 2 equiv of *N*-methylmorpholine (6.25 mmol, 0.71 mL) and 1.1 equiv of isobutyl chloroformate (3.58 mmol, 0.464 mL) were added dropwise, respectively. The reaction was then allowed to stir at -10 °C for 15 min. Next, 1.1 equiv of 4-nitro-*o*-phenylenediamine (3.58 mmol, 0.55 g) was added to the reaction. The reaction was then stirred for 2 h at -10 °C and then stirred at room temperature (RT) overnight under Ar. The resulting solution turned orange overnight. The reaction was then removed from argon flow and the solvent was removed under reduced pressure. The resulting orange solid was dissolved in *N*,*N*-dimethylformamide (DMF, 20 mL) and added to a saturated aqueous potassium chloride solution

(200 mL) to precipitate the desired product. This precipitate was removed from solution by vacuum filtration with a Büchner funnel and washed with cold water. The solid was then allowed to air dry for 2 h before being dried under vacuum. The resulting solid was still judged impure by thin layer silica chromatography (TLC) and purified further by flash chromatography by dissolving the solid in THF (5 mL) and loading it on silica (500 mL bed volume in 45:55 ethyl acetate:hexane). Compound **1** eluted with an R_f of 0.5 and was dried by rotary evaporation, washed with chloroform, and dried under high vacuum overnight. Compound **1** was obtained in 71.4 % yield (1.38 g, 2.32 mmol). HRMS (ESI) m/z calculated for $C_{34}H_{34}N_4O_6Na$ $[M+Na]^+$ is 617.2376, found 617.2379. ^1H NMR (500 MHz, C_2D_6OS): δ 9.44 (1 H, s), 8.13 (1 H, s), 7.88 (4 H, d, *J* = 7.5), 7.71 (2 H, d, *J* = 7.4), 7.41 (2 H, t, *J* = 7.4), 7.32 (2 H, q, *J* = 7.4), 7.25 (2 H, d, *J* = 8.6), 6.87 (2 H, d, *J* = 8.5), 6.78 (1 H, d, *J* = 9.1), 6.39 (2 H, s), 4.45 (1 H, d, *J* = 7.5), 4.28 – 4.15 (3 H, m), 3.13 – 2.89 (2 H, m), 2.80, 1.22 (9 H, s). ^{13}C NMR (126 MHz, C_2D_6OS): δ 171.1, 162.3, 156.1, 153.56, 149.3, 143.7, 140.7, 135.5, 132.2, 129.8, 127.6, 127.0, 125.3, 123.4, 123.4, 123.2, 121.8, 120.1, 113.6, 77.6, 65.8, 56.7, 46.6, 39.5, 36.7, 28.5.

(9*H*-fluoren-9-yl)methyl (*S*)-(1-((2-amino-5-nitrophenyl)amino)-3-(4-(*tert*-butoxy)phenyl)-1-thioxopropan-2-yl)carbamate (2). Sodium carbonate (0.246 g, 2.32 mmol) and phosphorus pentasulfide (1.03 g, 2.32 mmol) were added to THF (20 mL) in an oven-dried round bottom flask and allowed to stir under Ar at RT until the phosphorus pentasulfide was completely dissolved (30 min). Then the solution was cooled to 0 °C in an ice bath and **1** was dissolved in minimal THF and added to the reaction. The reaction was then monitored by TLC with a mobile phase of 2:3 ethyl acetate/hexanes. The product has an R_f of 0.4. After 4 h, the starting material was mostly consumed and a small amount of the benzimidazole byproduct had been formed, therefore the reaction was judged to be at completion. The reaction was condensed to dark yellow oil by rotary evaporation. This oil was then dissolved in minimal ethyl acetate and was loaded onto silica plug (bed volume approximately 400 mL in ethyl acetate) to remove the phosphorus pentasulfide. The crude product was eluted in ethyl acetate as a dark yellow band. This solution was dried by rotary evaporation and redissolved in 2:3 ethyl acetate/hexanes (5 mL). This mixture was purified by flash chromatography on silica with a running solvent of 2:3 ethyl acetate/hexanes. The product eluted as a yellow solution and solvent was removed under reduced pressure. The resulting yellow solid was washed with dichloromethane and chloroform, then dried under high vacuum overnight.

Compound **2** was obtained in 71.1% yield (1.01 g, 1.65 mmol). HRMS (ESI) m/z calculated for $C_{34}H_{35}N_4O_5S$ $[M+H]^+$ is 611.2328, found 611.2339. ^{1}H NMR (500 MHz, C_2D_6OS): δ 11.24 (1 H, s), 8.37 – 8.25 (1 H, m), 8.10 (1 H, d, *J* = 6.6), 7.94 (1 H, dd, *J* = 9.1, 2.7), 7.89 (2 H, d, *J* = 7.6), 7.73 (2 H, dd, *J* = 13.1, 7.5), 7.65 (1 H, d, *J* = 2.7), 7.42 (1 H, t, *J* = 7.5), 7.36 – 7.30 (1 H, m), 7.29 (2 H, d, *J* = 8.5), 6.91 (2 H, d, *J* = 8.4), 6.77 (1 H, d, *J* = 9.0), 6.25 (2 H, s), 4.68 (1 H, q, *J* = 7.2), 4.35 – 4.25 (1 H, m), 4.21 (2 H, q, *J* = 7.5), 3.14 (1 H, dd, *J* = 13.4, 7.2), 3.05 (1 H, dd, *J* = 13.4, 7.6), 1.26 (9 H, s). ^{13}C NMR (126 MHz, C_2D_6OS): δ 206.8, 156.2, 153.8, 150.5, 143.8, 143.6, 140.7, 135.3, 131.8, 130.0, 127.6, 127.0, 125.4, 125.3, 124.9, 124.4, 123.3, 122.2, 120.1, 113.8, 79.2, 77.7, 65.9, 62.6, 46.6, 39.5, 28.5.

(9*H*-fluoren-9-yl)methyl (*S*)-(3-(4-(*tert*-butoxy)phenyl)-1-(6-nitro-1*H*-benzo[*d*][1,2,3]triazol-1-yl)-1-thioxopropan-2-yl)carbamate (3, Fmoc-Tyr^S-NBt). Compound **2** (1.01 g, 1.65 mmol) was dissolved in glacial acetic acid diluted with 5% water (15 mL) and allowed to stir while cooling to 0 °C in an ice bath. After 5 min, 1.25 equiv of $NaNO_2$ (0.142 g, 2.06 mmol) was added slowly to the reaction. The reaction was monitored by TLC using 2:3 ethyl acetate in hexanes as the mobile phase; compound **3** has an R_f of 0.7. After 30 min, no starting material remained by TLC and the reaction became thick with an orange precipitate. Cold Milli-Q water (100 mL) was added to the reaction to precipitate the remaining product. The resulting orange precipitate was filtered on a Büchner funnel, washed with additional cold Milli-Q water, allowed to air dry for 20 min, and then dried under high vacuum overnight. The product was then lyophilized due to residual water remaining after high vacuum. The crude yield of the reaction was 82% (0.836 g, 1.35 mmol) and was used directly in solid phase peptide synthesis without further purification. HRMS (ESI) m/z calculated for $C_{34}H_{31}N_5O_5S$ $[M-H]^-$ is 621.2046, found 621.2055. ^{1}H NMR (500 MHz, $CDCl_3$): δ 9.62 (1 H, s), 8.44 (1 H, d, *J* = 8.6), 8.28 (1 H, d, *J* = 8.8), 7.77 (2 H, d, *J* = 6.9), 7.61 – 7.53 (3 H, m), 7.47 – 7.36 (4 H, m), 7.36 – 7.28 (3 H, m), 7.05 (2 H, d, *J* = 7.7), 6.82 (2 H, d, *J* = 7.8), 6.59 (1 H, d, *J* = 7.2), 5.74 (1 H, d, *J* = 8.9), 4.51 – 4.40 (2 H, m), 4.39 – 4.33 (1 H, m), 4.20 (1 H, s), 3.33 (1 H, dd, *J* = 13.5, 5.7), 3.15 (1 H, dd, *J* = 13.6, 7.5), 1.25 (9 H, s). ^{13}C NMR (126 MHz, $CDCl_3$): δ 208.4, 155.6, 154.9, 149.7, 149.2, 143.8, 141.5, 131.8, 129.9, 127.9, 127.2, 125.1, 124.3, 122.35, 121.7, 120.2, 112.7, 100.1, 78.6, 77.4, 77.2, 76.9, 67.3, 62.4, 47.3, 42.6, 28.9.

Synthesis and Purification of Thioisoleucine (Fmoc-Ile^S-NBt) Precursor

(9*H*-fluoren-9-yl)methyl ((2*S*,3*S*)-1-((2-amino-5-nitrophenyl)amino)-3-methyl-1-oxopentan-2-yl)carbamate (4). Fmoc-Ile-OH (3.53 g, 10.0 mmol) was dissolved in anhydrous THF (90 mL). N-methylmorpholine (2.20 mL, 20.0 mmol) was added and the reaction was cooled to -10 °C while the flask was purged with argon. Isobutyl chloroformate (1.30 mL, 10.0 mmol) was added dropwise while stirring. The reaction mixture was stirred for 15 min at -10 °C, then 4-nitro-*o*-phenylenediamine (1.53 g, 10.0 mmol) was added. The reaction was stirred under argon for 2 hours at -10 °C, then at room temperature overnight. After removing the solvent *in vacuo*, the residue was dissolved in DMF (50 mL). Upon addition of saturated aqueous KCl (250 mL) solution and Milli-Q water (250 mL), a yellow solid precipitated. The solid was filtered, washed with Milli-Q water and dried under vacuum. Compound **4** was isolated in 78.5% yield (3.84 g, 7.85 mmol) and 79.4% purity, as determined by HPLC. HRMS (ESI) m/z calculated for $C_{27}H_{28}N_4O_5$ $[M+H]^+$ is 489.2138, found 489.2130. ^{1}H NMR (500 MHz, C_2D_6OS): δ 9.54 (s, 1H), 8.29 (d, *J* = 2.7 Hz, 1H), 7.91 – 7.83 (m, 3H), 7.80 – 7.72 (m, 3H), 7.41 (t, *J* = 7.2 Hz, 4H), 7.32 (t, *J* = 7.5 Hz, 2H), 6.79 (d, *J* = 9.1 Hz, 1H), 6.52 (s, 2H), 4.35 – 4.20 (m, 4H), 4.09 (t, *J* = 8.2 Hz, 1H), 1.90 (ddt, *J* = 12.4, 8.5, 4.2 Hz, 1H), 1.56 (ddd, *J* = 13.7, 7.4, 3.3 Hz, 1H), 1.30 – 1.17 (m, 1H), 0.94 (d, *J* = 6.8 Hz, 3H), 0.88 (t, *J* = 7.4 Hz, 3H). ^{13}C NMR (126 MHz, C_2D_6OS): δ 171.2, 156.4, 148.8, 143.7, 140.7, 135.5, 127.6, 127.0, 125.3, 122.9, 121.2, 121.1, 120.1, 113.7, 65.8, 59.8, 46.7, 35.8, 24.7, 15.4, 10.7.

(9*H*-fluoren-9-yl)methyl ((2*S*,3*S*)-1-((2-amino-5-nitrophenyl)amino)-3-methyl-1-thioxopentan-2-yl)carbamate (5). Anhydrous Na_2CO_3 (0.239 g, 2.25 mmol) and P_4S_{10} (1.00 g, 2.25 mmol) were suspended in anhydrous THF (30 mL) in an oven-dried round bottom flask and stirred under argon for 30 minutes until solution became clear. Compound **4** (1.10 g, 3.00 mmol) was added and the reaction stirred over night at room temperature under argon. The next day the solvent was removed *in vacuo*. The resulting residue was resuspended in ethyl acetate and filtered over a pad of Celite® (Sigma-Aldrich, St. Louis, MO) to remove insoluble P_4S_{10} adducts. The filtrate was washed twice with 5% $NaHCO_3$ and once with brine. The aqueous layer was acidified with HCl and back extracted twice with ethyl acetate. The combined organic layers were dried over $MgSO_4$, filtered and the solvent was removed *in vacuo*. The crude product was redissolved

in dichloromethane and purified over silica to afford pure product as a yellow foam in 50.8% yield (0.770 g, 1.52 mmol). R_f = 0.5 in ethyl acetate/hexanes (1:1). HRMS (ESI) m/z calculated for $C_{27}H_{28}N_4O_4S$ $[M+H]^+$ is 505.1910, found 505.1909. ^1H NMR (500 MHz, $CDCl_3$) δ 9.57 (s, 1H), 7.98 (s, 1H), 7.95 (d, *J* = 8.9 Hz, 1H), 7.77 – 7.70 (m, 2H), 7.49 (d, *J* = 7.5 Hz, 1H), 7.44 (d, *J* = 7.4 Hz, 1H), 7.42 – 7.33 (m, 2H), 7.27 (s, 2H), 7.30 – 7.20 (m, 2H), 6.58 (d, *J* = 9.0 Hz, 1H), 5.70 (s, 1H), 4.27 (d, *J* = 7.7 Hz, 3H), 4.14 (t, *J* = 7.1 Hz, 1H), 2.10 – 2.02 (m, 1H), 1.29 – 1.20 (m, 2H), 1.02 (d, *J* = 6.7 Hz, 3H), 0.97 (t, *J* = 7.3 Hz, 3H). ^{13}C NMR (126 MHz, $CDCl_3$) δ 206.2, 157.4, 148.5, 143.3, 141.3, 138.4, 128.1, 128.0, 127.4, 127.3, 125.6, 125.0, 125.0, 124.9, 122.3, 120.3, 120.2, 115.3, 77.4, 67.6, 66.89, 47.0, 39.1, 29.83, 15.9, 11.1.

(9*H*-fluoren-9-yl)methyl ((2*S*,3*S*)-3-methyl-1-(6-nitro-1*H*-benzo[*d*][1,2,3]triazol-1-yl)-1-thioxopentan-2-yl)carbamate (6, Fmoc-Ile^S-NBt). Compound **5** (0.770 g, 1.52 mmol) was dissolved in 95% glacial acetic acid diluted with 5 % water (20 mL). The solution was cooled to 0 °C and $NaNO_2$ (156 mg, 2.26 mmol) was added slowly. Upon complete addition, the reaction mixture was stirred for 30 min at 0 °C, after which cold Milli-Q water (125 mL) was added. The precipitated orange solid was filtered, washed with cold Milli-Q water and dried *in vacuo*. The crude yield of the reaction was 57.7% (0.453 g, 0.88 mmol) and was used directly in solid phase peptide synthesis without further purification. R_f = 0.45 in dichloromethane. HRMS (ESI) m/z calculated for $C_{27}H_{25}N_5O_4S$ $[M+Na]^+$ is 538.1525, found 538.1508. ^1H NMR (500 MHz, $CDCl_3$) δ 9.70 (s, 1H), 8.46 (d, *J* = 8.7 Hz, 1H), 8.32 (d, *J* = 8.9 Hz, 1H), 7.76 (d, *J* = 7.6 Hz, 2H), 7.60 (d, *J* = 7.5 Hz, 2H), 7.40 (t, *J* = 7.5 Hz, 2H), 7.31 (q, *J* = 8.1 Hz, 2H), 6.22 (dd, *J* = 9.9, 6.2 Hz, 1H), 5.70 (d, *J* = 9.9 Hz, 1H), 4.49 (dd, *J* = 10.9, 6.9 Hz, 1H), 4.41 (dd, *J* = 10.9, 6.8 Hz, 1H), 4.22 (t, *J* = 6.9 Hz, 1H), 2.13 – 2.04 (m, 1H), 1.72 – 1.61 (m, 1H), 1.30 – 1.19 (m, 1H), 1.03 (d, *J* = 6.7 Hz, 3H), 0.91 (t, *J* = 7.4 Hz, 3H). ^{13}C NMR (126 MHz, $CDCl_3$) δ 209.5, 156.0, 149.5, 149.0, 143.6, 143.6, 141.3, 131.7, 127.7, 127.0, 127.0, 125.0, 124.9, 122.2, 121.5, 119.9, 112.8, 66.9, 65.7, 47.2, 41.1, 24.1, 16.2, 11.2.

Synthesis and Purification of Thiohydroxyproline (Fmoc-Hyp^S-NBt) Precursor

(9*H*-fluoren-9-yl)methyl (2*S*,4*R*)-2-((2-amino-5-nitrophenyl)carbamoyl)-4-(*tert*-butoxy) pyrrolidine-1-carboxylate (7). Fmoc-Hyp(*t*-Bu)-OH (4.09 g, 10.0 mmol) was dissolved in THF

(75 mL) under argon flow with stirring in a -10 °C NaCl/ice bath. To this solution, 2.0 equiv of *N*-methylmorpholine (20.0 mmol, 2.2 mL) and 1.0 equiv of isobutyl chloroformate (10.0 mmol, 1.3 mL) were added dropwise, respectively. The mixture was then stirred for 10 min and 4-nitro-*o*-phenylenediamine (1.53 g, 10.0 mmol) was added to the reaction. The resulting slurry was then stirred at -20 °C for 1 hour. The precipitate that formed was filtered off and the filtrate was evaporated to dryness. The residue was dissolved in ethyl acetate and washed twice with 10% $KHCO_3$. The organic layer was concentrated under reduced pressure and dissolved in DMF (36 mL). To this solution, saturated aqueous potassium chloride solution (180 mL) was added to precipitate the desired product. This precipitate was removed from solution by vacuum filtration with a Büchner funnel and washed with cold water. The solid was then dried under vacuum overnight. Compound **7** was obtained in an isolated yield of 96.6 % (5.26 g, 9.66 mmol) and has an R_f of 0.2 in 2:3 ethyl acetate:hexanes. HRMS (ESI) m/z calculated for $C_{30}H_{32}N_4O_6$ $[M+Na]^+$ is 545.2400, found 545.2401. ^1H NMR (500 MHz, $CDCl_3$): δ 8.35 (s, 1H), 8.13 (s, 1H), 7.80 – 7.45 (m, 5H), 7.42 – 7.20 (m, 4H), 6.49 (d, *J* = 9.0 Hz, 1H), 4.92 (s, 2H), 4.52 – 4.14 (m, 4H), 3.74 (dd, *J* = 10.5 Hz, *J* = 6.0 Hz, 1H), 3.40 (dd, *J* = 10.5 Hz, *J* = 4.0 Hz, 1H), 2.34 (m, 1H), 2.09 (m, 1H), 1.22 (s, 9H). ^{13}C NMR (125 MHz, $CDCl_3$): δ 170.7, 156.0, 147.9, 143.4, 143.3, 141.1, 137.6, 127.7, 127.1, 127.0, 124.9, 124.8, 123.7, 122.5, 120.5, 119.9, 119.9, 114.1, 76.8, 69.3, 67.9, 59.6, 53.8, 46.8, 37.0, 28.1.

(9*H*-fluoren-9-yl) methyl(2*S*,4*R*)-2-((2-amino-5-nitrophenyl)carbamothioyl)-4-(*tert*-butoxy)pyrrolidine-1-carboxylate (8). Sodium carbonate (1.02 g, 9.66 mmol) and phosphorus pentasulfide (4.30 g, 9.66 mmol) were added to THF (64 mL) in an oven-dried round bottom flask and allowed to stir under Ar at RT for 1 h. To this mixture, compound **7** (5.26 g, 9.66 mmol) was added and the reaction mixture was stirred overnight at RT. The reaction was monitored by TLC with a mobile phase of 2:3 ethyl acetate/hexanes. The product has an R_f of 0.3. At reaction completion, THF was removed by rotary evaporation and ethyl acetate was added to the resulting residue. The suspension formed was passed through Celite® and the filtrate was washed with 5% $KHCO_3$ (2x) and brine. The organic layer was dried over Na_2SO_4, filtered, and concentrated. Further purification was conducted with silica gel column chromatography. Compound **7** was obtained in 75% yield (4.06 g, 7.25 mmol). HRMS (ESI) m/z calculated for $C_{30}H_{32}N_4O_5S$ $[M+Na]^+$ is 583.1991, found 583.2015. ^1H NMR (500 MHz, $CDCl_3$): δ 9.26 (s, 1H), 8.01 (s, 1H), 7.95 –

7.45 (m, 5H), 7.42 – 7.20 (m, 4H), 6.55 (m, 1H), 5.14 – 4.15 (m, 7H), 3.80 (m, 1H), 3.51 (m, 1H), 2.53 – 2.23 (m, 2H), 1.20 (s, 9H). ^{13}C NMR (125 MHz, $CDCl_3$): 205.5, 171.2, 156.1, 149.1, 143.4, 141.1, 137.3, 127.7, 127.7, 127.1, 127.0, 125.5, 124.9, 124.9, 121.6, 119.9, 114.4, 74.3, 69.4, 67.8, 66.9, 60.3, 54.7, 46.9, 41.1, 28.2, 20.9, 14.0.

(9*H*-fluoren-9-yl)methyl (2*S*,4*R*)-4-(*tert*-butoxy)-2-(6-nitro-1*H*-benzo[*d*][1,2,3]triazole-1-carbonothioyl)pyrrolidine-1-carboxylate (9, Fmoc-Hyp^S-NBt). Compound **8** (0.77 g, 1.37 mmol) was dissolved in glacial acetic acid diluted with 10% Milli-Q water (15 mL) and allowed to stir while cooling to 0 °C in an ice bath. Then $NaNO_2$ (0.143 g, 2.06 mmol) was added slowly to the reaction. The reaction was monitored by TLC using 3:7 ethyl acetate in hexanes as the mobile phase; compound **9** has an R_f of 0.35. After 30 min, cold Milli-Q water (70 mL) was added to the reaction to precipitate the product. The resulting orange precipitate was filtered on a Büchner funnel, washed with additional cold Milli-Q water. The orange solid was re-dissolved in dichloromethane and the solution was dried over Na_2SO_4, then evaporated to dryness. Isolated yield for compound **9** was 75% (0.59 g, 1.03 mmol). HRMS (ESI) m/z calculated for $C_{30}H_{29}N_5O_5S$ $[M+Na]^+$ is 594.1787, found 594.1786. ^{1}H NMR (500 MHz, CDCl3, rotameric mixtures): δ 9.46 (9.60) (d, J = 1.5 Hz, 1H), 8.53-8.18 (m, 2H), 7.78-6.82 (m, 8H), 5.85 (6.31) (m, 1H), 4.79 – 3.41 (m, 6H) 2.72-1.85 (m, 2H), 1.09 (1.23) (s, 9H). ^{13}C NMR (125 MHz, $CDCl_3$, rotameric mixtures): δ 206.3 (207.4), 153.5 (154.4), 148.6 (149.3), 148.6 (149.2), 143.4 (143.8), 143.3 (143.6), 141.1, 140.7 (140.6), 131.7 (131.8), 126.9 (127.6), 126.9 (126.7), 126.7 (126.6), 124.9 (125.0), 123.9 (123.7), 121.9, 121.0 (121.2), 119.8, 119.0 (119.0), 113.1 (112.6), 74.0 (74.1), 68.0 (69.2), 66.8 (67.4), 66.0 (65.0), 54.1 (54.2), 47.0 (47.05), 41.7 (41.4), 28.0 (28.2).

Synthesis and Purification of Thioglutamate (Fmoc-Glu^S-NBt), Thiophenylalnine (Fmoc-Phe^S-NBt), Thioalanine (Fmoc-Ala^S-NBt), Thioleucine (Fmoc-Leu^S-NBt), Thioproline (Fmoc-Pro^S-NBt), Thioglycine (Fmoc-Gly^S-NBt), and Thiovaline (Fmoc-Val^S-NBt) Precursors. Each of these precursors were synthesized using modified synthesis from Rapoport *et al*. and have been reported previously.[1-3, 6, 8, 9]

Calmodulin Synthesis and Analysis

Peptide Synthesis of $CaM_{135-148}$ Cys_{135} Phe^{S}_{138}. The first CaM thiopeptide construct made was $CaM_{135-148}Cys_{135}Phe^{S}_{138}$. Residue 138 is a tyrosine in the native sequence, but Phe^{S} was used for the initial method development as it had been previously synthesized and used in our lab for ligation. Solid phase peptide synthesis (SPPS) of $CaM_{135-148}$ Cys_{135} Phe^{S}_{138} (H_2N-CVNF^{S}EEFVQMMTAK-OH) was performed on 2-chlorotrityl resin (100-200 mesh) on a 50 µmol scale. The peptide was elongated up to the thioamide position using standard SPPS protocols by adding 5 equiv of amino acid, HBTU, and DIPEA in DMF (2 mL) and stirring each coupling for 40 min at RT. Fmoc removal was performed by stirring the resin in 20% piperidine in DMF (5 mL) for 20 min at RT. It should be noted that each amino acid coupling was performed twice to improve isolated peptide yields (data not shown). Prior to the thioamide coupling, the resin was washed with dry dichloromethane (DCM, obtained by drying over molecular sieves; washed 2 x 4 mL). Fmoc-Phe^{S}-NBt (1.5 eq.) was then added to the vessel with DIPEA (1.5 equiv) in DCM (1.5 mL) and the reaction was stirred at RT for 30 min. The vessel was then drained and the resin washed with dry DCM (2 x 4 mL) before repeating the thioamide coupling. At completion of the second coupling, the resin was washed with DCM, then DMF (2 x 4 mL each) and standard SPPS was used to add the remaining amino acids. After the Fmoc deprotection of Cys_{135}, the resin was washed with DMF and DCM, before being dried by vacuum for 10 min. A cleavage cocktail was prepared with 24:16:1:1:1 DCM:TFA:TIPS:EDT:Thioanisole (total volume of 4.3 mL). This was added to the dried resin and allowed to stir at RT for 1 h before being drained and collected. DCM (4 mL) was added to the resin to wash out any remaining peptide and the collected solution was dried to a yellow oil by rotary evaporation. This oil was washed with DCM (3 x 5 mL) to remove any excess organic reagents. The crude peptide product was then precipitated by adding cold diethyl ether (10 mL) and vortexing vigorously. The resulting precipitate was centrifuged to a pellet and the supernatant was discarded. Crude peptide was stored under argon at 4 °C until purification by HPLC could proceed.

HPLC Purification of $CaM_{135-148}$ Cys_{135} Phe^{S}_{138}. The crude white solid was dissolved in 35% v/v acetonitrile in Milli-Q water with 0.1 % TFA. The peptide was purified by reverse phase HPLC using a binary solvent system (Buffer A: water with 0.1% TFA; Buffer B: acetonitrile with 0.1%

TFA). A Grace Vydac C18 Preparatory column was used with the gradient in **Table S1** to purify $CaM_{135\text{-}148}$-Cys_{135} Phe^{S}_{138}.

Table S1: Solvent gradient used for HPLC purification.

Time (min)	Buffer A (%)
0:00	98
5:00	98
10:00	75
34:00	62
35:00	2
40:00	2
41:00	98
46:00	98

Synthesis of Remaining CaM Thiopeptides. Synthesis of each CaM thiopeptide was performed as described above. MALDI (**Table S2**) and MALDI tandem MS/MS (data not shown) analysis was used to confirm the mass and intact thioamide presence in each peptide. We routinely obtained 2-6 mg (2-7% yield) of thiopeptide with these syntheses.

Table S2: Calculated and observed m/z for isolated CaM thiopeptides.

$CaM_{135\text{-}148}$ Sequence	Calculated m/z $[M+H]^+$	Observed m/z
H_2N-CV^{S}NYEEFVQMMTAK-OH	1709.72	1709.94
H_2N-CVNF^{S}EEFVQMMTAK-OH	1693.73	1693.74
H_2N-CVNY^{S}EEFVQMMTAK-OH	1709.72	1710.25
H_2N-CVNYE^{S}EFVQMMTAK-OH	1709.72	1709.47
H_2N-CVNYEE^{S}FVQMMTAK-OH	1709.72	1709.21
H_2N-CVNYEEF^{S}VQMMTAK-OH	1709.72	1710.28
H_2N-CVNYEEFV^{S}QMMTAK-OH	1709.72	1709.37
H_2N-CVNYEEFVQMMTA^{S}K-OH	1709.72	1710.19

Expression and Purification of WT and Cys_{135} CaM. Expression (1 L in LB media) and purification of these CaM constructs were done as previously reported.[8] Yields for each protein

totaled ~20 mg/L and each protein was stored as a lyophilized powder prior to further processing and preparation for CD measurements.

Cloning of CaM$_{1\text{-}134}$-GyrA-H6. Full length CaM was previously cloned into the pTXB1 vector containing the MxeGyrA intein and a six residue histidine tag for Ni-NTA purification.[10] The 14 amino acid deletion was carried out in four rounds of QuikChange® PCR using the following four sets of primers. Deletions were confirmed by DNA sequencing with T7 promoter and terminator primers.

i. Deletion of CaM$_{146\text{-}148}$
Forward: 5'-GAAGAGTTTGTACAGATGATGTGCATCACGGGAGAT-3'
Reverse: 5'-ATCTCCCGTGATGCACATCATCTGTACAAACTCTTC-3'

ii. Deletion of CaM$_{142\text{-}145}$
Forward: 5'-CAAGATAACTATGAAGAGTTTTGCATCACGGGAGAT-3'
Reverse: 3'-ATCTCCCGTGATGCAAAACTCTTCATAGTTTACTTG-3'

iii. Deletion of CaM$_{138\text{-}141}$
Forward: 5'-GATGGTGATGGTCAAGTAAACTGCATCACGGGAGAT-3'
Reverse: 5'-ATCTCCCGTGATGCAGTTTACTTGACCATCACCATC-3'

iv. Deletion of CaM$_{135\text{-}137}$
Forward: 5'-GACATTGATGGTGATGGTTGCATCACGGGAGAT-3'
Reverse: 5'-ATCTCCCGTGATGCAACCATCACCATCAATGTC-3'

Original Protein Sequence:

(M)ADQLTEEQIAEFKEAFSLFDKDGDGTITTKELGTVMRSLGQNPTEAELQDMINEVDA
DGNGTIDFPEFLTMMARKMKDTDSEEEIREAFRVFDKDGNGYISAAELRHVMTNLGEK
LTDEEVDEMIREADIDGDGQVNYEEFVQMMTAKCITGDALVALPEGESVRIADIVPGAR
PNSDNAIDLKVLDRHGNPVLADRLFHSGEHPVYTVRTVEGLRVTGTANHPLLCLVDVA
GVPTLLWKLIDEIKPGDYAVIQRSAFSVDCAGFARGKPEFAPTTYTVGVPGLVRFLEAH
HRDPDAQAIADELTDGRFYYAKVASVTDAGVQPVYSLRVDTADHAFITNGFVSHATGL
TGLKLHHHHHH

Resulting Protein Sequence:

(M)ADQLTEEQIAEFKEAFSLFDKDGDGTITTKELGTVMRSLGQNPTEAELQDMINEVDA
DGNGTIDFPEFLTMMARKMKDTDSEEEIREAFRVFDKDGNGYISAAELRHVMTNLGEK
LTDEEVDEMIREADIDGDGCITGDALVALPEGESVRIADIVPGARPNSDNAIDLKVLDRH
GNPVLADRLFHSGEHPVYTVRTVEGLRVTGTANHPLLCLVDVAGVPTLLWKLIDEIKPG
DYAVIQRSAFSVDCAGFARGKPEFAPTTYTVGVPGLVRFLEAHHRDPDAQAIADELTDG
RFYYAKVASVTDAGVQPVYSLRVDTADHAFITNGFVSHATGLTGLKLHHHHHH

The sequence in black is the WT calmodulin sequence. The portion in blue is the deleted sequence to be added by NCL with Cys in place of Gln at the ligation site. The sequence in green is the Mxe GyrA intein followed by the His_6 tag in red. The initial methionine (M) is removed during expression by an endogenous *Escherichia Coli* methionine aminopeptidase.

Cloning of CaM_{1-71}-GyrA-CBD. The gene encoding for $CaM_{S17C,\ 1-116}$ is in the pTXB1 vector which contains a GyrA intein from the *Mycobacterium xenopi* followed by a chitin-binding domain (CBD) as a purification tag. A PacI site was introduced at Met_{71} and Leu_{116} of the CaM gene using QuikChange® mutagenesis. With the PacI sites encoded, the plasmid was digested with PacI and then the resulting sticky ends were ligated using T4 DNA ligase resulting in a pTXB1-CaM_{1-71}-PacI plasmid. Next, the PacI site was mutated back to wild type Met_{71}. The Cys_{17} mutation was then reverted back to Ser_{17} to return the gene to the native sequence. Primers used for each set of mutations are shown below:

i. Insertion of PacI at Met_{71}
Forward: 5'- CAATTGACTTCCCAGAGTTTCTTAATTAAATGGCGCGCAAAATGAAAGATAC-3'
Reverse: 5'- GTATCTTTCATTTTGCGCGCCATTTAATTAAGAAACTCTGGGAAGTCAATTG -3'

ii. Insertion of PacI at Leu_{116}
Forward: 5'- GTGATGACAAACCTTGGTGTTAATTAATGCATCACGGGAGATGCACTAG -3'
Reverse: 5'- CTAGTGCATCTCCCGTGATGCATTAATTAACACCAAGGTTTGTCATCAC -3'

iii. Change $PacI_{71}$ to Met_{71}
Forward: 5'- CAATTGACTTCCCAGAGTTTCTGACAATGTGCATCACGGGAGATGCAC -3'
Reverse: 5'- GTGCATCTCCCGTGATGCACATTGTCAGAAACTCTGGGAAGTCAATTG -3'

iv. Revert to Cys_{17} to $Ser_{17.}$
Forward: 5'- CAGAGTTCAAAGAGGCTTTTAGCCTG -3'
Reverse: 5'- CAGGCTAAAAGCCTCTTTGAACTCTG -3'

CaM_{1-134}-GyrA-H_6 Expression. CaM_{1-134}-GyrA was transformed into BL21-Gold (DE3) *E. Coli* cells and grown against ampicillin (100 µg/mL) on an LB-agar plate. Single colonies were picked and grown in liquid LB media (3 x 5 mL, 100 µg/mL ampicillin) with shaking (250 RPM) at 37 °C until saturation. Each primary culture was then added to a secondary culture of autoclaved LB media (1 L, 100 µg/mL ampicillin) and grown at 37 °C, with shaking (250 RPM) until OD_{600}= 0.8-1.0 (3-4 h). IPTG was then added (final concentration = 1 mM) and the temperature and shaking speed were reduced to 25 °C and 225 RPM respectively for 16 h of additional incubation.

$CaM_{1\text{-}134}$-GyrA-H_6 Purification. Cells (3 x 1 L expression) were harvested by centrifugation at 5,000 RPM in a GS3 rotor and Sorvall RC-5 centrifuge for 10 min at 4 °C. The supernatant was discarded and the cell pellet was suspended in 25 mL lysis buffer (50 mM HEPES, pH 7.5) containing two broad spectrum protease inhibitor tablets. Resuspended cells were then lysed on ice by sonication (30 amps power, 1 second pulse, 1 second rest, 2 minute total sonication time) and then pelleted at 13,000 RPM in an SS-34 rotor (Sorvall RC-5 centrifuge) for 15 min at 4 °C. The supernatant was collected and incubated with Ni^{2+}-NTA resin (4 mL column volume) for 1 h on ice with shaking. The slurry was then added to a fritted column and the liquid was allowed to flow through. The resin was then washed with 3 x 10 mL of buffer (50 mM HEPES, pH 7.5) and 3 x 10 mL of wash buffer (50 mM HEPES, 10 mM imidazole, pH 7.5). $CaM_{1\text{-}134}$-GyrA was then eluted from the resin in 5 fractions each containing 5 mL of elution buffer (50 mM HEPES, 300 mM imidazole, pH 7.5). The pooled fractions were dialyzed against MES•Na cleavage buffer (20 mM Tris, pH 7.3) overnight at 4 °C.

$CaM_{1\text{-}134}$-GryA-H_6 MES•Na Intein Cleavage and Purification of $CaM_{1\text{-}134}$-MES Thioester. MES•Na (200 mM final concentration) was added to 25 mL of the solution containing $CaM_{1\text{-}134}$-GyrA in MES•Na cleavage buffer and allowed to incubate with stirring for 16 h at 4 °C. After 16 h, a 1:30 dilution in water of the cleavage solution was analyzed by MALDI-MS and the reaction appeared to be at completion. The cleavage reaction was dialyzed against 20 mM Tris, pH 8.0 at RT for 2 h to remove the remaining MES•Na from solution. $CaM_{1\text{-}134}$-MES was purified in three batches over a HiTrap Q column using a 120 min NaCl gradient (0.1 M to 0.8 M NaCl in 20 mM Tris, pH 8.0). The desired product partially coelutes with the impurity $CaM_{1\text{-}135}$-OH, a hydrolytic byproduct of the intein cleavage reaction, which can be resolved after ligation. This eluent was collected in 6 x 1.5 mL fractions (product presence confirmed by MALDI-MS). The pooled fractions were dialyzed against Milli-Q water at 4 °C overnight before being flash frozen with liquid nitrogen and lyophilized to a solid. Total yield from a 3 x 1 L expression is approximately 30 mg of 90% pure $CaM_{1\text{-}134}$-MES.

Purification of $CaM_{1\text{-}134}$-OH. $CaM_{1\text{-}134}$-GyrA-H_6 was expressed as above. After Ni^{2+}-NTA purification, the eluent was dialyzed against 20 mM Tris pH 7.3 (2 L) overnight at 4 °C. $CaM_{1\text{-}134}$-GyrA-H_6 was then treated with βME (200 mM) to cleave the intein and reveal $CaM_{1\text{-}134}$-OH. This

cleavage process is discussed elsewhere.[10] This protein was then purified by anion exchange over a HiTrap Q column as described above for $CaM_{1\text{-}134}$-MES. The protein was sufficiently pure after one round of FPLC purification and was dialyzed against Milli-Q water overnight at 4 °C before being frozen in liquid nitrogen and lyophilized to a white powder. The total isolated yield of $CaM_{1\text{-}134}$-OH was 8 mg per L of culture.

Expression and Purification of $CaM_{1\text{-}71}$-OH. $CaM_{1\text{-}71}$-GyrA-CBD was transformed into BL21-Gold (DE3) *E. coli* cells and grown on an LB-agar plate with ampicillin (100 μg/mL). A single colony was picked and grown in liquid LB media (5 mL, 100 μg/mL ampicillin) with shaking (250 RPM) at 37 °C until saturation. The primary culture was then added to a secondary culture of sterile Terrific Broth media[11] (1 L, 100 μg/mL ampicillin) and grown at 37 °C, shaking (250 RPM) until OD_{600}= 0.8-1.0 (3-4 h). IPTG was then added (final concentration = 1 mM) and the temperature and shaking speed were reduced to 25 °C and 225 RPM. The culture was allowed to grow for 16 h. Cells were harvested by centrifugation at 5,000 RPM in GS3 rotor and Sorvall RC-5 centrifuge for 10 min at 4 °C. The supernatant was discarded and the cell pellet was resuspended in 10 mL lysis buffer (20 mM Tris, pH 8.0) containing one broad spectrum protease inhibitor tablet. Resuspended cells were then lysed on ice by sonication (30 amps power, 1 second pulse, 1 second rest, 2 minute total sonication time) and spun down at 13,000 RPM in an SS-34 rotor in a Sorvall RC-5 centrifuge for 15 min at 4 °C. The supernatant was collected and incubated with chitin beads (5 mL column volume, CV) for 1 h on ice with shaking. The slurry was then added to a fritted column and the liquid was allowed to flow through. The beads were washed with 10 x CV of 20 mM Tris pH 8.0. Intein cleavage buffer (20 mM Tris, 200 mM βME, pH 7.5, 10 mL) was then added to the beads and incubated on a rotisserie at RT for 16 h. The column was then drained and the flow through collected. The eluent was dialyzed against 20 mM Tris, pH 8.0 to remove any excess βME and purified by FPLC using anion exchange as described above. The protein was pure after one round of FPLC purification and was dialyzed against Milli-Q water overnight at 4 °C before being frozen in liquid nitrogen and lyophilized to white powder. The total isolated yield was 6 mg per L of culture.

Native Chemical Ligation (NCL) for Synthesis of Thioamide CaM Constructs. The following general protocol was used for the synthesis of all thioamide containing constructs. Ligation buffer (2 mL) was prepared fresh prior to each reaction by adding NaH_2PO_4 (0.2 M), Gdn•HCl (6 M), TCEP•HCl (20 mM) and thiophenol (2% v/v) to 1.7 mL of water and adjusting the pH to 7.3 followed by the volume to 2 mL. Approximately 10 mg (660 nmol) of $CaM_{1\text{-}134}$-MES of was weighed out using an analytical balance and added to an Eppendorf tube. To this tube, ligation buffer (500 μL) was added and the tube was vortexed to ensure all dried protein dissolved and then incubated at RT for 2 min to allow the more electrophilic $CaM_{1\text{-}134}$-SPh to form by transthioesterification of the MES thioester with thiophenol (see **Scheme S2**). Thiopeptide (2 equiv) was then weighed out into a separate Eppendorf tube and ligation buffer (500 μL) was added. This tube was then vortexed to ensure that all of the thiopeptide dissolved. The thiopeptide was then added to the tube containing the $CaM_{1\text{-}134}$-SPh protein and incubated at 37 °C with constant shaking (500 RPM) for 20 h. The reaction was monitored by MALDI for product formation and it should be noted that additional $CaM_{1\text{-}134}$-OH byproduct was formed during the reaction. At reaction completion, the mixture was diluted to 3 mL with Milli-Q water and dialyzed against 20 mM Tris pH 8.0 overnight at 4 °C in preparation for FPLC purification.

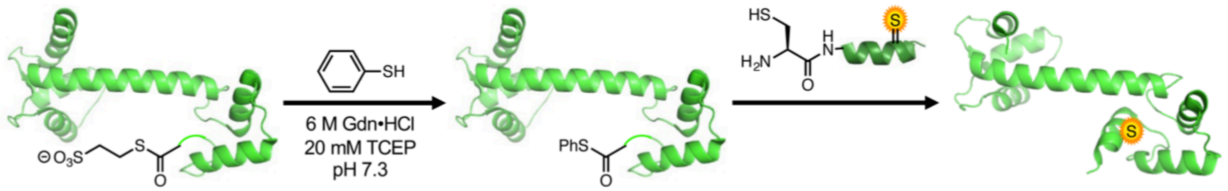

Scheme S2. NCL methodology used to generate all CaM thiproteins. Transthioesterification of $CaM_{1\text{-}134}$-MES with thiophenol, followed by ligation to $CaM_{135\text{-}148}$-Cys_{135} thiopeptide.

NCL Control for CaM Cys_{135}. The expressed CaM Cys_{135} protein was subjected to NCL conditions by incubating it with ligation buffer for 20 h with constant shaking (500 RPM) at 37 °C. This protein was then subjected to the same purification and alkylating protocols as the thioamide CaM mutants.

Purification of Thioamide CaM NCL Reactions. The dialysate from the crude reaction was filtered and treated with TCEP Bond-Breaker™ (5 μL) prior to being manually loaded onto a 4 mL bed volume HiTrap Q column. The purification proceeded using the same gradient as the

CaM_{1-134}-MES thioester purification. The full-length CaM thioprotein elutes over 5 fractions with CaM_{1-134}-OH but the two proteins cannot be completely separated using any gradient on anion exchange. To remove the impurity, a cysteine capture thiopropyl sepharose 6b resin is used. The NCL product containing a cysteine will form a covalent disulfide bond with the thiopropyl group while the impurity CaM_{1-134}-OH will flow through the resin. Prior to purification by this resin, the fractions from anion exchange purification were dialyzed against 2 L thiopropyl binding buffer (20 mM Tris, 1 mM EDTA pH 7.5) overnight at 4 °C. The resin (0.6 g, 1.5 mL CV) is prepared by washing it with 50 x CV of Mili-Q water, followed by 10 x CV of binding buffer. The dialysate is then added to the slurry and incubated on a rotisserie for 1 h at RT. The flow through is collected and analyzed by MALDI to ensure that the thioamide CaM bound to the resin. The resin was then washed with 10 x CV of binding buffer, 25 x CV of wash buffer (20 mM Tris, 100 mM NaCl, 1 mM EDTA, pH 7.5), and again with 10 x CV binding buffer. Elution buffer is prepared by adding βME to a final concentration of 30 mM in 6 mL binding buffer. Elution buffer (4 mL) is then added to the resin and incubated on a rotisserie for 20 min at RT. The eluent is collected and the resin washed with the remaining 2 mL of elution buffer, followed by 3 mL of binding buffer to elute the remaining protein. The presence of thioamide CaM is determined by MALDI by observing the mass of the βME disulfide adduct (M+76 Da). This solution is then dialyzed against 2 L of capping buffer (20 mM Tris, pH 8.3) for 2 h at RT before proceeding to the alkylation of Cys_{135}.

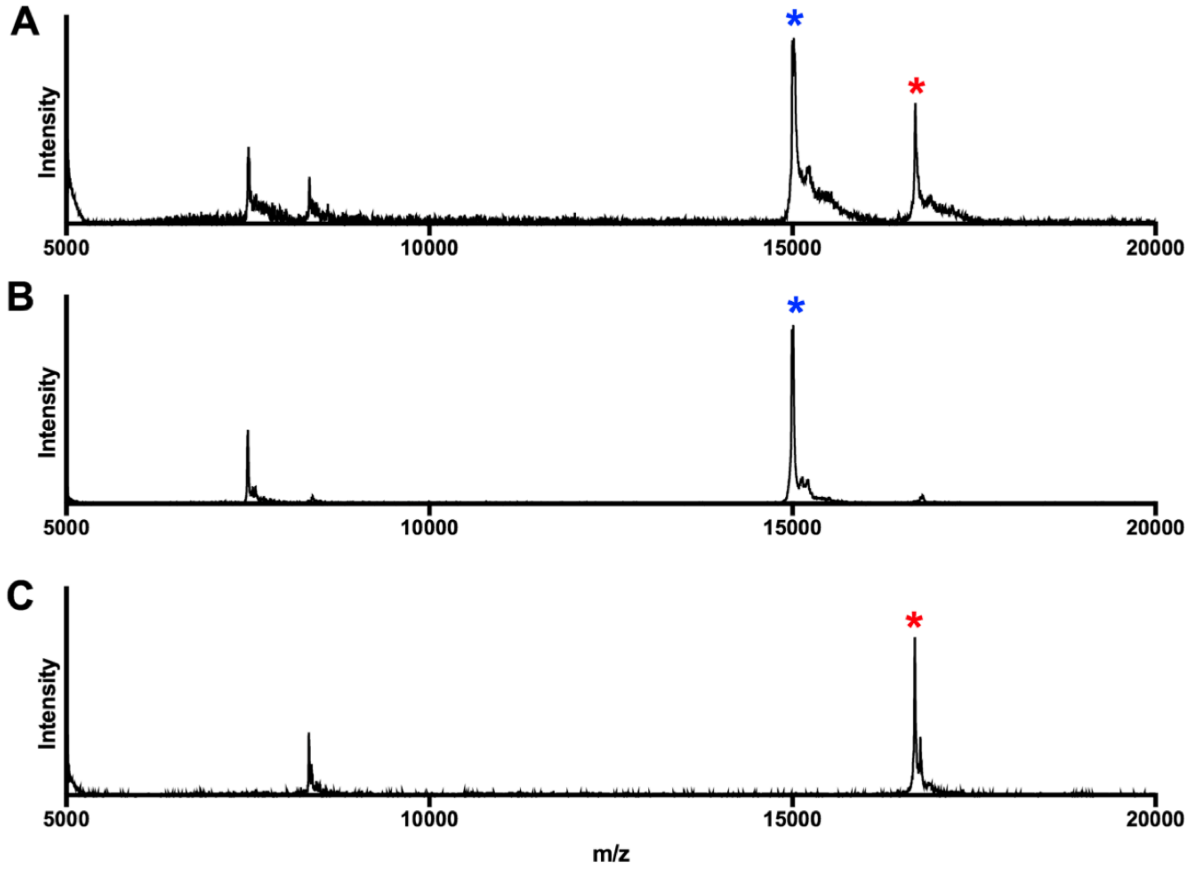

Fig. S1: MALDI mass spectra analysis of purification of CaM-$Cys_{135}Phe^S{}_{138}$ by thiopropyl sepharose resin. (A) Mixture of $CaM_{1\text{-}134}$ (*) and $CaM_{1\text{-}148}$-$Cys_{135}Phe^S{}_{138}$ (*) prior to resin incubation. (B) Flow through containing only $CaM_{1\text{-}134}$ (calculated m/z $[M+H]^+$ = 15008 Da; observed 15008 Da) after resin incubation. (C) Elution containing only the desired $CaM_{1\text{-}148}$-$Cys_{135}Phe^S{}_{138}$ NCL product (calculated m/z $[M+H]^+$ = 16682 Da; observed 16682 Da).

Alkylation of Cys_{135} with Iodoacetamide. The dialysate from the previous step was added to a 15 mL Falcon tube and incubated with TCEP Bond-Breaker™ (10 mM) for 2 min. Next, iodoacetamide (final concentration = 200 mM) was added and the solution was vortexed slowly until all of the iodoacetamide was dissolved. The reaction was incubated at RT for five min and the presence of singly alkylated thioamide CaM or CaM-Cys_{135} was confirmed by MALDI-MS (**Table S3**). This solution was then dialyzed against 2 L of water for 2 h, followed by a second round of dialysis overnight at 4 °C to remove the excess TCEP and iodoacetamide. The resulting solution was flash frozen in liquid nitrogen and lyophilized to a fine white powder.

Table S3: Calculated and observed protein m/z. All CaM thioproteins routinely fly as $[M+Na]^+$.

CaM Variant	Calculated m/z $[M+H]^+$	Calculated m/z $[M+Na]^+$	Observed m/z
WT	16708	16730	16705
$CaM_{1\text{-}134}$-OH	15008	15030	15007
$CaM_{1\text{-}134}$-MES	15130	15152	15130
$CaM_{1\text{-}71}$	7832	7854	7832
CaM-Cys^{Q}_{135}	16740	16762	16740
CaM-$Cys^{Q}_{135}Val^{S}_{136}$	16756	16778	16777
CaM-$Cys^{Q}_{135}Phe^{S}_{138}$	16740	16762	16762
CaM-$Cys^{Q}_{135}Tyr^{S}_{138}$	16756	16778	16777
CaM-$Cys^{Q}_{135}Glu^{S}_{139}$	16756	16778	16778
CaM-$Cys^{Q}_{135}Glu^{S}_{140}$	16756	16778	16775
CaM-$Cys^{Q}_{135}Phe^{S}_{141}$	16756	16778	16778
CaM-$Cys^{Q}_{135}Val^{S}_{142}$	16756	16778	16775
CaM-$Cys^{Q}_{135}Ala^{S}_{147}$	16756	16778	16777

Trypsin Digest of CaM-Cys^{Q}_{135} Ala^{S}_{147}. To a 5 µL aliquot of sequencing grade trypsin (0.1 mg/mL), 50 µL of a 25 µM stock of CaM-$Cys^{Q}_{135}Ala^{S}_{147}$ was added. This mixture was incubated at 37 °C for 16 h. The resulting tryptic peptide mixture was analyzed by MALDI. The desired $[M+Na]^+$ (expected: 2561.8 Da, observed: 2561.2 Da) was detected for the peptide H_2N-EADIDGDGC^{Q}VNYEEFVQMMTA^{S}K-OH obtained from the cut site at Arg_{126}. The spectrum was absent of any peaks indicating multiple or missed alkylations by iodoacetamide. To ensure that Cys_{135} was alkylated while leaving the thioamide sulfur intact, MALDI-MS/MS of this peptide was performed. The resulting mass spectrum could be assigned for fragmentation to the x-, y-, b- and a-series of this peptide (**Figs. S2** and **S3**). Fragments could be unambiguously assigned to a peptide containing the alkylated Cys^{Q} and unmodified Ala^{S}_{147} without any detectable missed or aberrant alkylations, respectively (**Table S4**).

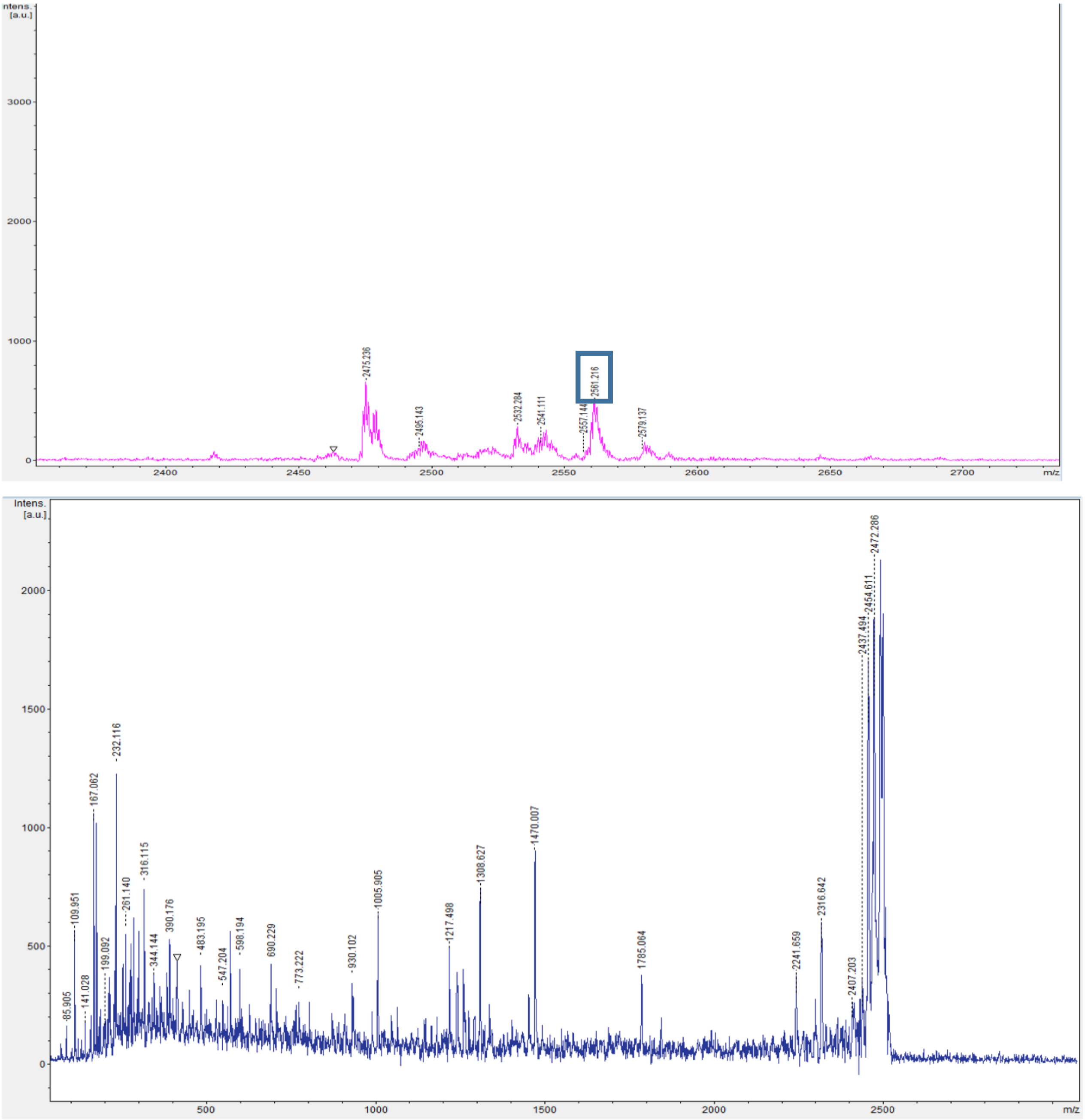

Fig. S2: (Top) Trypsin digest. The blue box indicates the desired peak selected for MS/MS ($[M+Na]^+$expected: 2561.8 Da, observed: 2561.2 Da). (Bottom) MS/MS spectrum of fragmented tryptic peptide. Ions from the a-,b-,x- and y-series are observed.

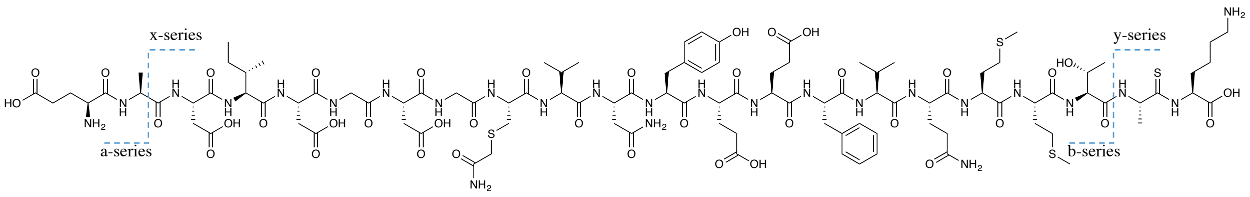

Fig. S3: Fragmentation patterns of the H_2N-EADIDGDGC[Q]VNYEEFVQMMTA[S]K-OH tryptic peptide.

Table S4: Expected (black) and observed (red) MS/MS fragment m/z (a-, b-, x- and y- series ions) for the tryptic peptide H_2N-EADIDGDGC[Q]VNYEEFVQMMTA[S]K-OH. Nearly complete coverage of the peptide is observed.

Sequence	Observed Series	a (m/z)	Obs a (m/z)	b (m/z)	Obs b (m/z)	x (m/z)	Obs x (m/z)	y (m/z)	Obs y (m/z)
E	b	102.113		130.123	129.031	---		2539.811	
A	a,b,x,y	173.192	175.091	201.202	200.068	2436.690	2437.508	2410.696	2412.637
D	a,b	288.280	288.113	316.290	316.115	2365.611		2339.617	
I	a,b	401.440	401.163	429.450	429.166	2250.522		2224.528	
D	b	516.528		544.538	547.022	2137.363		2111.369	
G	a	573.580	571.227	601.590		2022.274		1996.280	
D	a	688.669	690.220	716.679		1965.222		1939.228	
G	b	745.721		773.731	773.218	1850.134		1824.140	
C	a,b	905.899	930.107	933.909	934.077	1793.082		1767.088	
V	a	1005.032	1005.909	1033.042		1632.903		1606.909	
N	---	1119.136		1147.146		1533.771		1507.776	
Y	b	1282.312		1310.322	1308.626	1419.667		1393.672	
E	b,x	1411.427		1439.437	1452.515	1256.491	1258.493	1230.496	
E	---	1540.543		1568.553		1127.375		1101.381	
F	---	1687.719		1715.729		998.260		972.266	
V	a	1786.852	1785.055	1814.862		851.083		825.089	
Q	x	1914.983		1942.993		751.951	773.218	725.956	
M	y	2046.175		2074.185		623.820		597.826	598.194
M	---	2177.368		2205.378		492.627	495.222	466.633	
T	x,y	2278.473		2306.483		361.435	361.199	335.441	334.140
A	a,x,y	2365.612	2387.506	2393.622		260.330	261.142	234.335	235.096
K	a,x,y	2493.786	2495.692	2521.796		173.191	175.091	147.197	

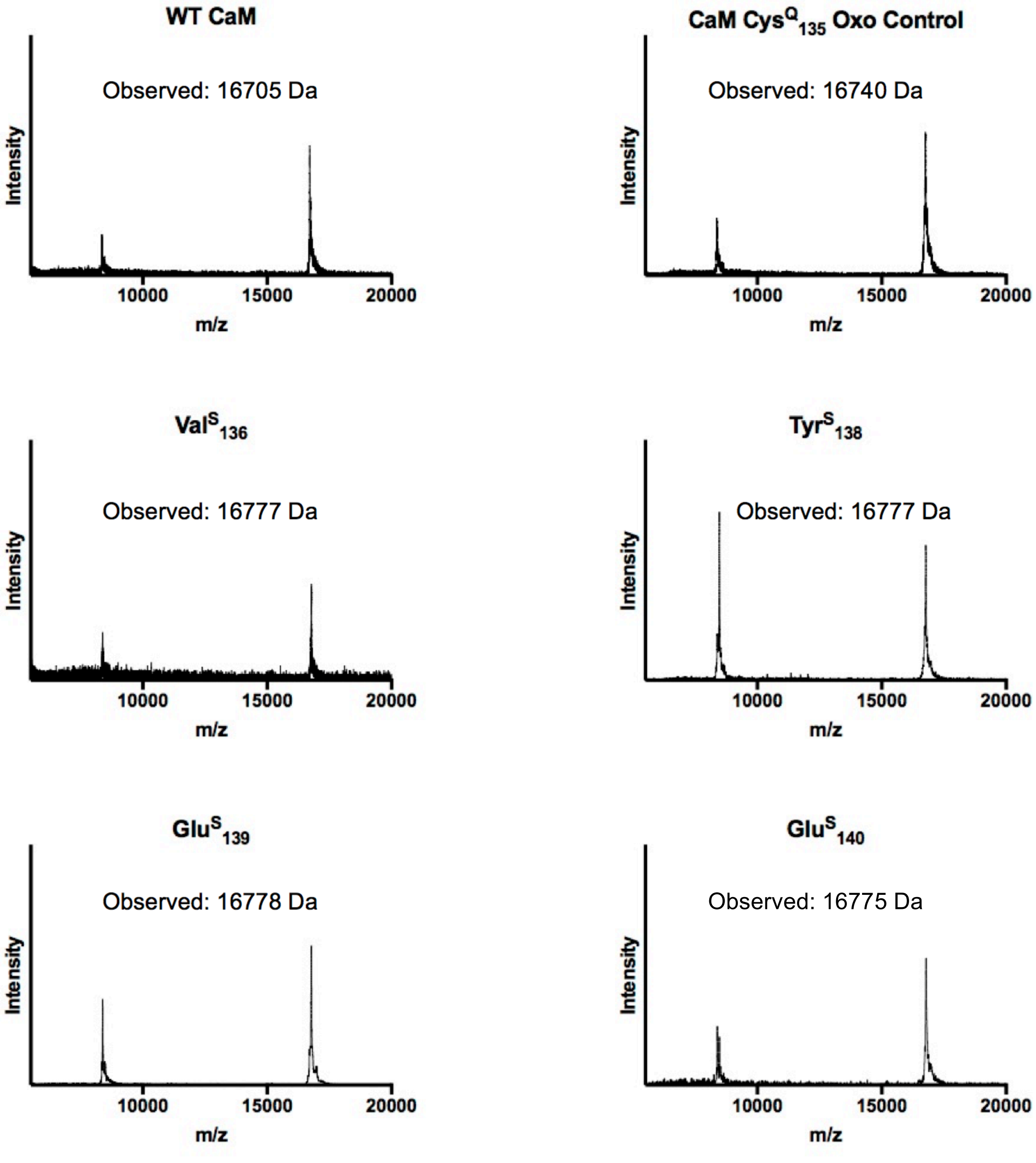

Fig. S4a: MALDI MS spectra showing CaM proteins (WT CaM, Cys^{Q}_{135}, Val^{S}_{136}, Tyr^{S}_{138}, $Glu^{S}_{139,}$ and Glu^{S}_{140}; see **Table 3** for observed and expected molecular weights) without any impurities. CaM proteins routinely fly as the M and M/2 ions.

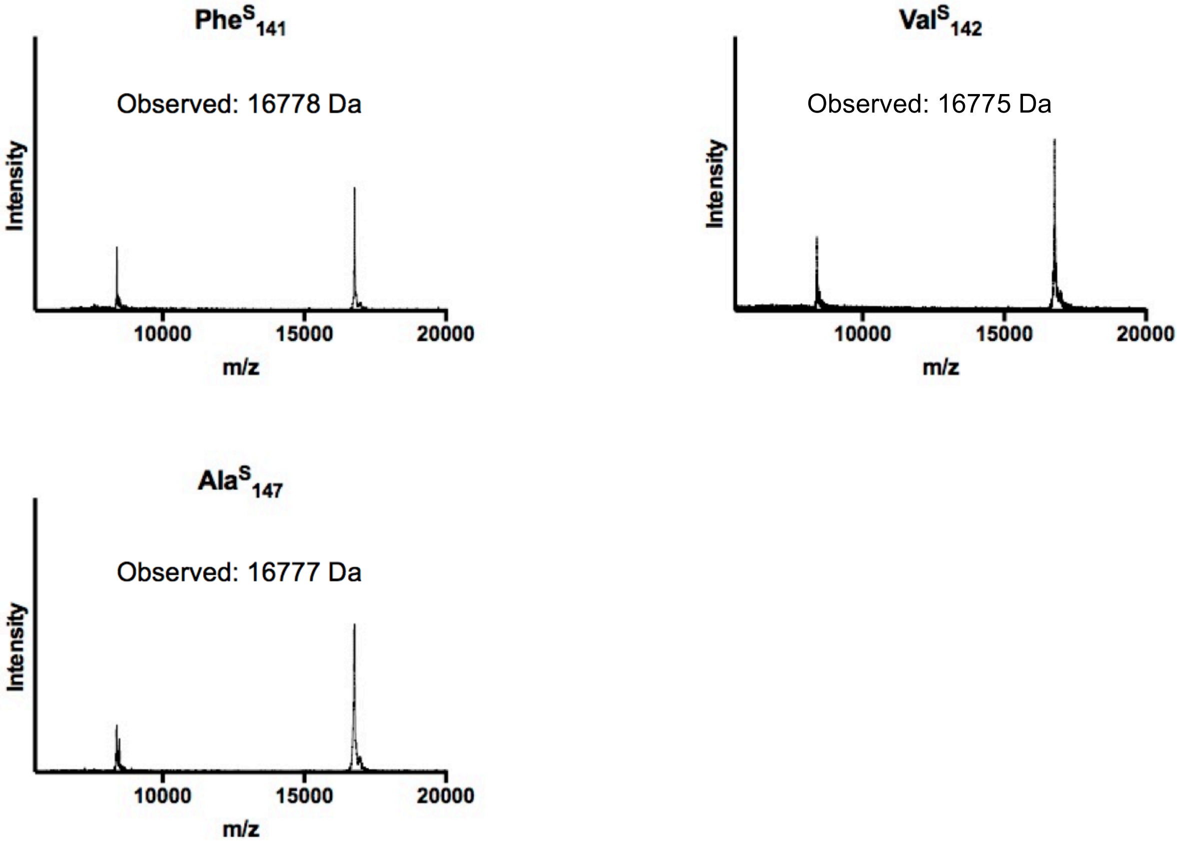

Fig. S4b: MALDI MS spectra showing CaM proteins (Phe^{S}_{141}, Val^{S}_{142}, and Ala^{S}_{147}; see **Table 3** for observed and expected molecular weights) without any impurities. CaM proteins routinely fly as the M and M/2 ions.

Table S5: Synthesized CaM variants and their yields based upon BCA quantification prior to CD measurements.

CaM Variant	Percent Yield
CaM-$Cys^{Q}_{135}Val^{S}_{136}$	7.0%
CaM-$Cys^{Q}_{135}Tyr^{S}_{138}$	13.9%
CaM-$Cys^{Q}_{135}Glu^{S}_{139}$	7.6%
CaM-$Cys^{Q}_{135}Glu^{S}_{140}$	13.0%
CaM-$Cys^{Q}_{135}Phe^{S}_{141}$	8.2%
CaM-$Cys^{Q}_{135}Val^{S}_{142}$	24,8%
CaM-$Cys^{Q}_{135}Ala^{S}_{147}$	11.2%

Preparation of Samples for Circular Dichroism (CD) Measurements. For each pure CaM variant, the lyophilized powder was dissolved in 2 mL of holo CD buffer (10 mM Tris, 2 mM $CaCl_2$, pH 7.5) and incubated for 10 min at RT to anneal the protein. This solution was then aliquoted into 2 x 1 mL samples. The holo aliquot for Ca^{2+} bound CaM measurements was dialyzed once more against 2 L of holo buffer for 2 h at RT to ensure any remaining iodoacetamide or TCEP salt from the capping reaction was removed. The apo aliquot for Ca^{2+} free CaM measurements was dialyzed against 2 L of calcium stripping buffer (10 mM Tris, 1 mM EDTA, pH 7.5) for 2 h at RT before an additional round of dialysis against 2 L of apo CD buffer (10 mM Tris, 0.5 mM EDTA, pH 7.5) for 2 h at RT. The dialysis buffers were saved to be used for further dilutions of the samples as well as background measurements in CD spectra acquisition.

Modified BCA Assay for Concentration Determination of CaM CD Samples. A commercial Pierce™ Thermo Fisher Scientific BCA assay kit (Bellefonte, PA) was used to determine the concentration of each sample of CaM. However, the assay was modified slightly to give more consistent concentration readings amongst replicates of samples. BCA reagent was prepared as described by the commercial protocol. A set of standard bovine serum albumen (BSA) dilutions (1.25, 1.0, 0.75, 0.5, 0.25, and 0.125 mg/mL) were used as a calibration curve. A separate calibration curve was generated for holo and apo samples as each buffer affects the absorbance measurement differently. BCA reagent (200 µL) was added to each empty Eppendorf tube for standards and samples. A 0 mg/mL BSA control tube was also used. Then each standard and sample (25 µL) were added to their corresponding BCA reagent tubes. The 0 mg/mL sample received either holo or apo CD buffer. The samples were then vortexed to mix and incubated at 37 °C for 30 min. Each sample was then diluted with 1.2 mL of buffer and vortexed to mix. Absorbance at 562 nm (A_{562}) was obtained by a HP 8452 UV/Vis spectrophotometer in a 1 cm path length cuvette. The A_{562} values were fit linearly and the fit was used to determine the concentration of the CaM samples. This method was compared to CaM quantification based upon the A_{276} and a previously published extinction coefficient (ε_{276} = 3006 M^{-1} cm^{-1}) and was in good agreement for WT CaM samples.[12] However, direct quantification by A_{276} for thioamide containing constructs using this method is not viable as the extinction coefficient in this absorbance region for each thioamide position in the context of the protein is unknown.

CD Wavelength Measurements. Based upon the concentrations obtained from the BCA assay, each holo and apo protein was divided into three aliquots and diluted to 10 μM in their respective buffers with the exception of Tyr^{S}_{138}, which was diluted to 30 μM to obtain a sufficiently high signal-to-noise ratio for CD measurements. The wavelength measurements were taken in triplicate to average out any small error in the concentration measurements or dilutions. Each sample (300 μL) was added to a 1 mm path length Helma Analytics 110-QS CD cuvette and loaded into a Jasco J-1500 CD Spectrometer. CD wavelength scans were taken at 25 °C by scanning at a 1 nm step between 350 nm and 190 nm with a bandwidth of 1 nm and an averaging time of 8 s per measurement. Background spectra were obtained by measuring the CD signal of the relevant buffer in each cuvette with an averaging time of 2 s. The raw signal (θ_d, mDeg) from each sample was background subtracted against the blanks and converted to molar residue ellipticity (θ_{MRE}) using

$$\theta_{MRE} = \theta_d/(cln_R) \tag{S1}$$

where c is the concentration (M), l is the path length (1 mm), and n_R is the number of residues in the protein. The resulting averaged scans for each holo and apo protein along with a table of θ_{222} and θ_{208} values are shown in **Fig. S5** and **Table S6**. Plots showing non-residue adjusted molar ellipticity (θ_n) for holo and apo $CaM_{1\text{-}135}$ and $CaM_{1\text{-}71}$ truncations, as well as selected thioproteins, are shown in **Fig. S6**.

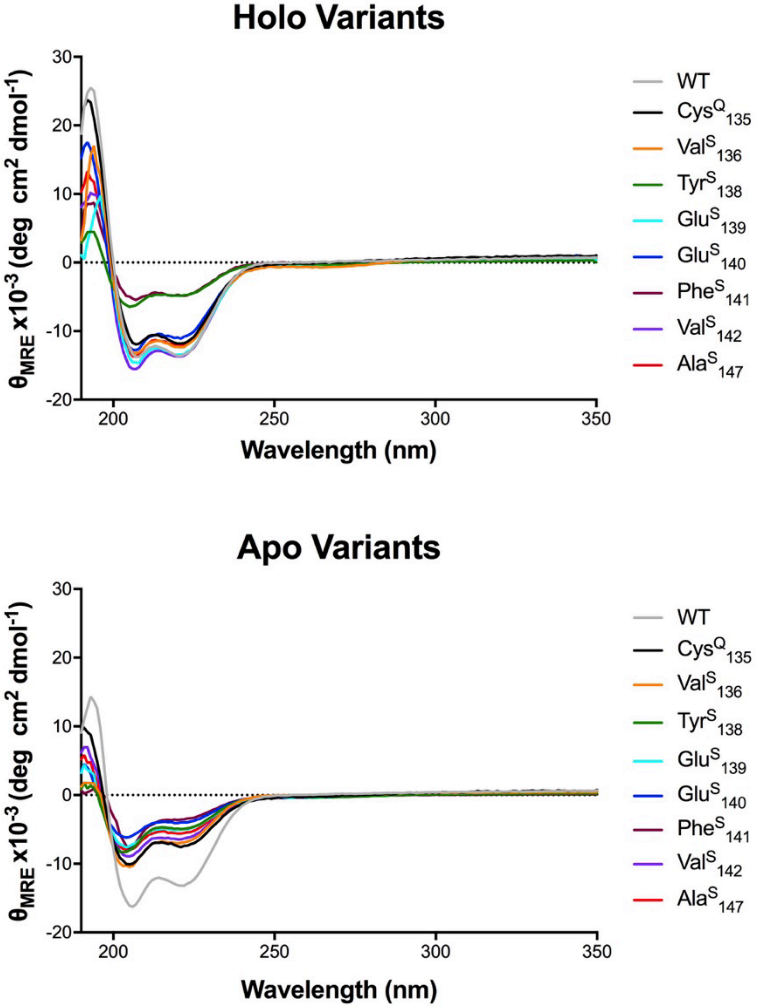

Fig. S5: Holo (top) and apo (bottom) θ_{MRE} spectra for the WT, $Cys^{Q}{}_{135}$, and all thioCaM variants. $Tyr^{S}{}_{138}$, $Glu^{S}{}_{140}$, and $Phe^{S}{}_{141}$ show the largest disruptions in helicity.

Table S6: Average values for θ_{222} (holo and apo) and θ_{208} (holo) MREs from three replicate spectra. The ratio between θ_{222} and θ_{208} is only given for the holo proteins due to the shift in global minima from 208 to 205 nm between holo and apo CaM.

CaM Variant	Holo θ_{222}	Holo θ_{208}	Holo $\theta_{222}/\theta_{208}$	Apo θ_{222}
WT	-13.40	-13.74	0.98	-13.20
Cys^{Q}_{135}	-11.73	-11.85	0.99	-7.39
Val^{S}_{136}	-12.31	-13.07	0.94	-6.89
Tyr^{S}_{138}	-4.77	-5.91	0.81	-4.92
Glu^{S}_{139}	-13.36	-14.63	0.91	-4.98
Glu^{S}_{140}	-10.80	-12.55	0.86	-3.99
Phe^{S}_{141}	-4.80	-5.22	0.92	-3.52
Val^{S}_{142}	-13.56	-15.24	0.89	-6.33
Ala^{S}_{147}	-12.03	-13.39	0.90	-5.52

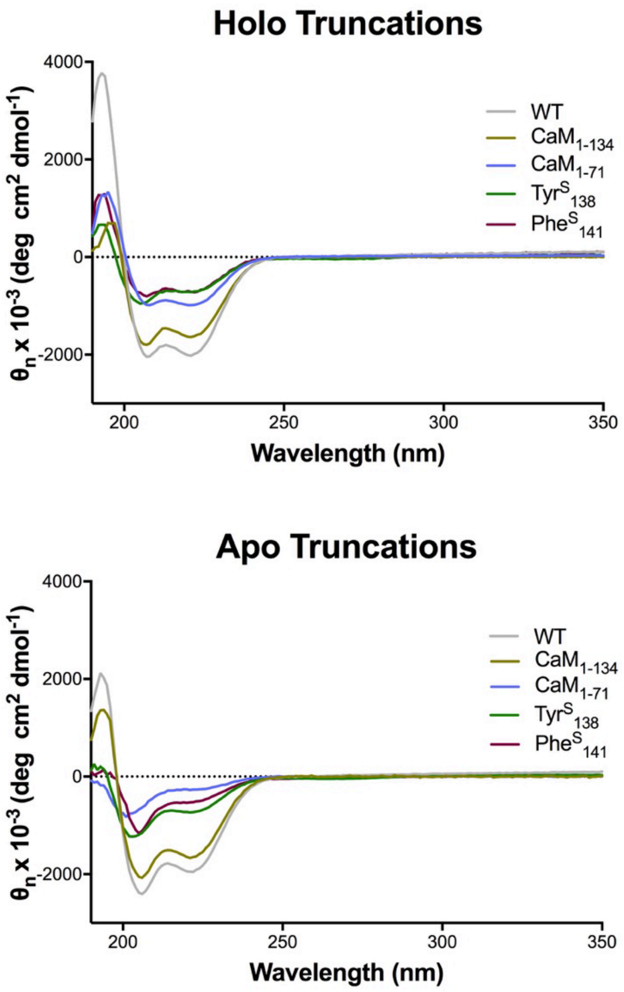

Fig. S6: Molar ellipticity (θ_n) of holo CaM truncations (top) and apo CaM truncations (top). Tyr$^S_{138}$ and Phe$^S_{141}$ modifications are more disruptive than losing the C-terminal helix (CaM$_{1\text{-}134}$), but not as disruptive as removal of the C-terminal domain (CaM$_{1\text{-}71}$) in the apo structure.

Thermal Denaturation of CaM Measured by CD. Thermal denaturation was performed by monitoring θ_d at 222 nm over a temperature range of 5 to 95 °C. The temperature increase was set to 0.5 °C per min with an equilibration time of 10 s. Measurements were made with an averaging time of 8 s per sample and a bandwidth of 1 nm. All holo samples did not completely melt over this temperature range and have melting temperatures (T_M) greater than 85 °C (**Fig. S7**). All apo proteins melted over this temperature range with significant initial transitions prior to global unfolding. Fitting procedures for raw apo melting data are described in the following sections.

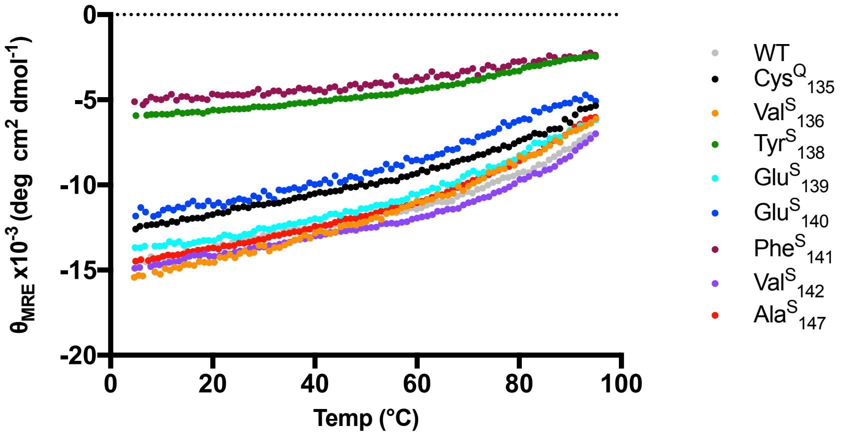

Fig. S7: Thermal melting profiles of holo CaM variants plotted as θ_{MRE} transformed data.

Two State Fit of Apo CaM CD Data. The two state model assumes that there is a single transition between the folded and unfolded states, with no intermediate state populated. For this model, the folded and unfolded linear baselines were fit between 5 to 20 °C and 65 to 80 °C, respectively, by using the low temperature ($\theta_f = m_f T + b_f$) and high temperature ($\theta_u = m_u T + b_u$) equations. The entire data set was then fit to

$$\theta_{fit} = \theta_f(T)(1 - F_{calc}) + \theta_u(T)(F_{calc}) \qquad F_{calc} = \frac{e^{-(\Delta H + T\Delta S)/RT}}{1+e^{-(\Delta H + T\Delta S)/RT}} \tag{S2}$$

where ΔH and ΔS are adjustable parameters and R = 1.9872 cal•mol^{-1}•K^{-1}. The fraction unfolded curve is generated by minimizing the root mean square difference (RMSD) between F_{calc} and the experimental fraction unfolded (F_{exp}, calculated from the experimental θ_{MRE} using equation S3) against the parameters listed above over the entire temperature range.

$$F_{exp} = \frac{\theta_{MRE} - \theta_f}{\theta_u - \theta_f} \tag{S3}$$

The F_{calc} fits of the θ_{MRE} data and fraction folded ($1 - F_{calc}; F_f$) plots for all controls and mutants can be seen in **Figs. S8** and **S9**. The T_Ms were calculated by $T_M = \frac{\Delta H}{\Delta S}$, assuming the free energy $\Delta G_U = 0$ at the midpoint of the transition. The free energy at 25 °C ($\Delta G_U(25)$) was determined by $\Delta G = \Delta H - T\Delta S$ using the ΔH and ΔS calculated from the fits and T = 298.15 K. All data fitting was performed using the solver function in Microsoft Excel (Redmond, WA) and the plots were rendered in GraphPad Prism (LaJolla, CA). We note that although the $\Delta\Delta G_U(25)$ values obtained from these two state fits are unreasonably low, using the simple analysis based on ΔT_M values in equation S8, we obtain $\Delta\Delta G_U$* values that are in reasonable agreement with the three state unfolding data given in **Table 1** in the main text.

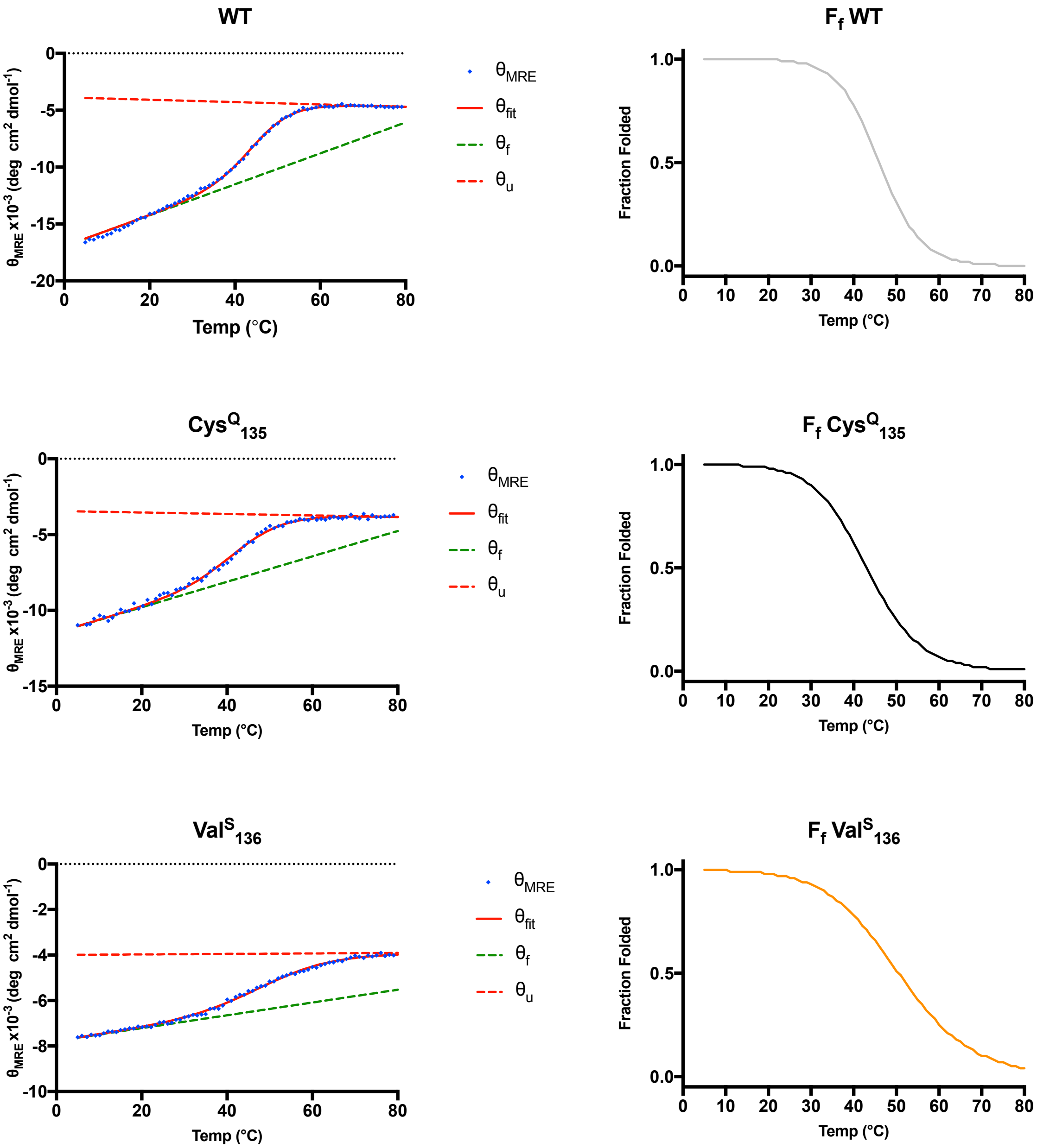

Fig. S8a. Plots generated using the two state fitting method for WT, Cys^{Q}_{135}, and Val^{S}_{136} . Left: For each CaM variant a single set of raw data (θ_{MRE}) and fits from equations for θ_{fit}, θ_f, and θ_u. Right: The averaged fraction folded plots (1-F_{calc}) from three replicated melts.

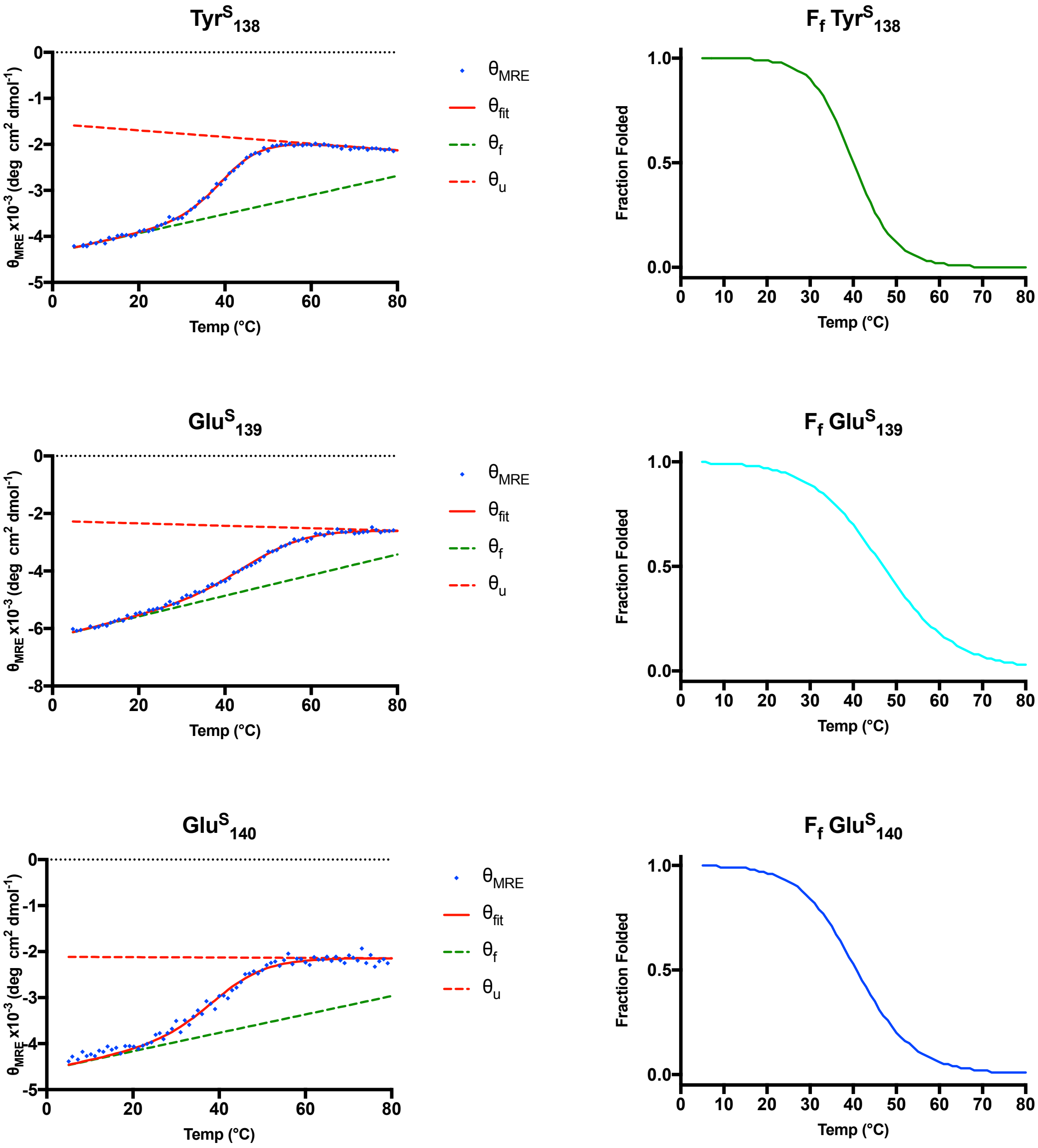

Fig. S8b. Plots generated using the two state fitting method for Tyr^{S}_{138}, Glu^{S}_{139}, and Glu^{S}_{140}. Left: For each CaM variant a single set of raw data (θ_{MRE}) and fits from equations for θ_{fit}, θ_{f}, and θ_{u}. Right: The averaged fraction folded plots (1-F_{calc}) from three replicated melts.

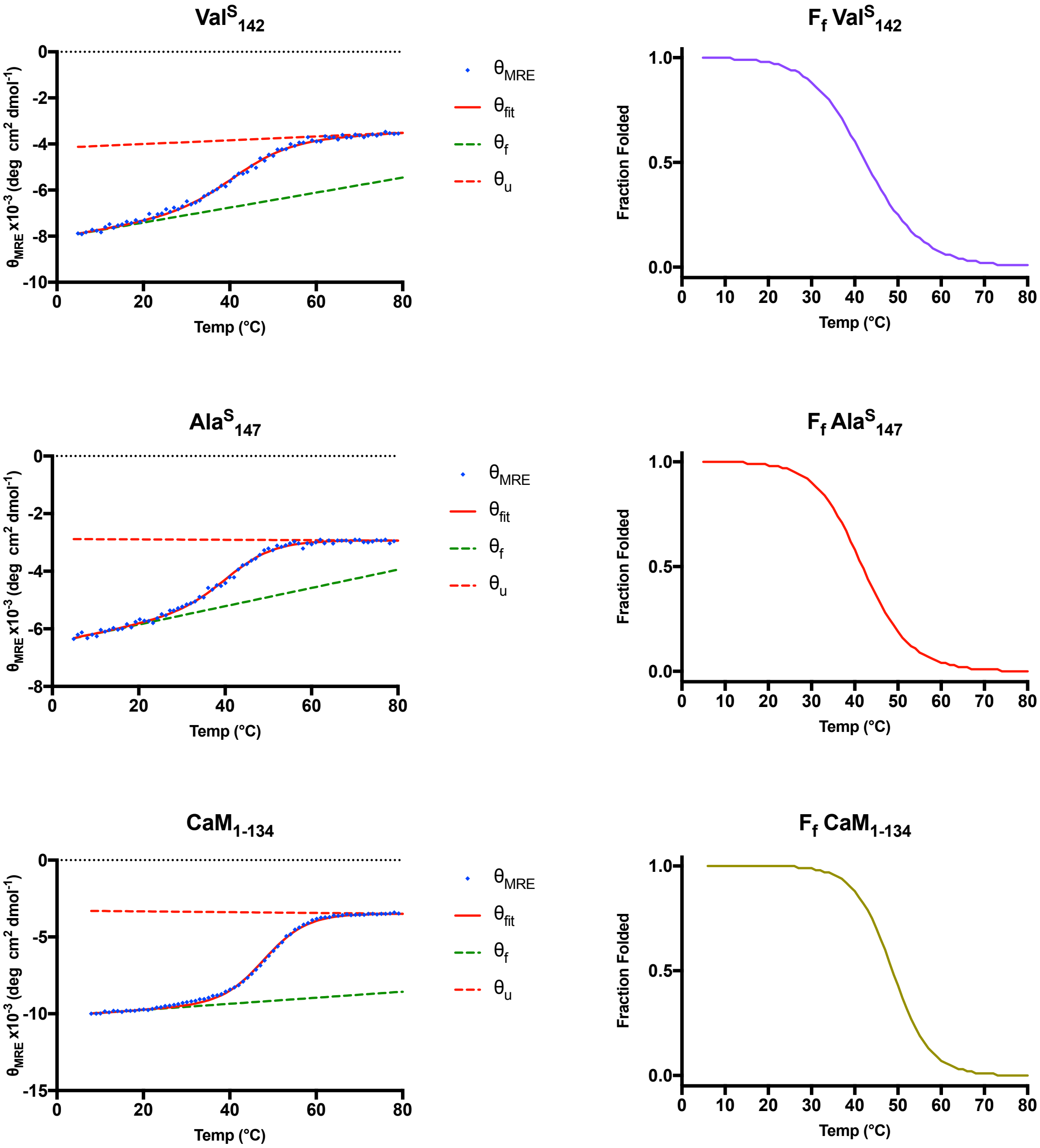

Fig. S8c: Plots generated using the two state fitting method for Val^{S}_{142}, Ala^{S}_{147}, and $CaM_{1\text{-}134}$. Left: For each CaM variant a single set of raw data (θ_{MRE}) and fits from equations for θ_{fit}, θ_{f}, and θ_{u}. Right: The averaged fraction folded plots ($1\text{-}F_{calc}$) from three replicated melts.

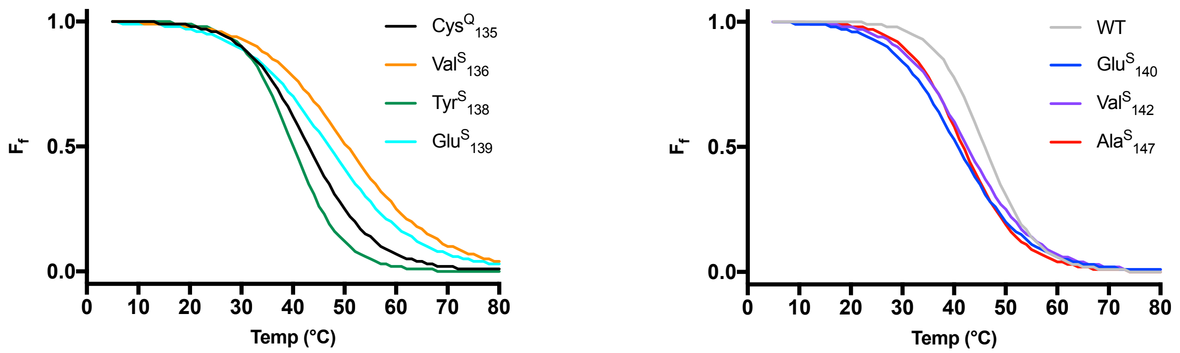

Fig. S9: Overlay of average fraction unfolded (F_{calc}) plots generated from the two state fitting analysis. Left: Cys^{Q}_{135}, Val^{S}_{136}, Tyr^{S}_{138}, and Glu^{S}_{139}. Right: WT, Glu^{S}_{140}, Val^{S}_{142}, and Ala^{S}_{147}.

Table S7: Average values for the thermodynamic parameters determined from the two state fits of F_{calc}. ΔT_M and $\Delta\Delta G_U$ are values relative to the Cys^{Q}_{135} control.

CaM Variant	T_M (°C)	ΔT_M (°C)	ΔH (kcal mol^{-1})	ΔG_U(25) (kcal mol^{-1})	$\Delta\Delta G_U$(25) (kcal mol^{-1})	$\Delta\Delta G_U$* (kcal mol^{-1})
WT	46.0 ± 0.1	---	41.9 ± 1.1	2.74 ± 0.08	---	
$CaM_{1\text{-}134}$	48.7 ± 0.3	---	48.5 ± 5.7	3.54 ± 0.43	---	
Cys^{Q}_{135}	44.1 ± 0.2	---	31.5 ± 2.2	1.88 ± 0.15	---	
Val^{S}_{136}	50.6 ± 0.6	+6.5	24.3 ± 0.3	1.91 ± 0.05	+0.03	-0.71
Tyr^{S}_{138}	40.1 ± 0.6	-4.0	40.9 ± 0.3	1.95 ± 0.09	+0.07	0.44
Glu^{S}_{139}	46.8 ± 1.7	+2.7	24.5 ± 2.7	1.70 ± 0.31	-0.18	-0.29
Glu^{S}_{140}	40.8 ± 0.7	-3.3	29.5 ± 1.8	1.47 ± 0.08	-0.41	0.36
Val^{S}_{142}	42.7 ± 0.4	-1.4	30.7 ± 2.0	1.70 ± 0.09	-0.18	0.15
Ala^{S}_{147}	41.8 ± 0.8	-2.3	35.9 ± 2	1.90 ± 0.18	+0.02	0.25

* Calculated from ΔT_M using equation S8.

Three State Fit of Apo CaM Data. In this model, the protein undergoes two transitions: F→I and I→U, where F, I, and U are the folded, intermediate, and unfolded states, respectively. This fit is more intuitive for CaM as the N- and C-terminal domains behave almost independently and have different melting temperatures.[13-15] The equations used here are adopted from Shea *et al.*[14] The folded, intermediate, and unfolded baselines were fit to $\theta_f = m_f T + b_f$, $\theta_i = m_i T + b_i$, and $\theta_u = m_u T + b_u$, respectively. In this case, m_i was constrained to the average of m_f and m_u as previously described.[14] The full data set was then fit to

$$\theta_{fit} = (\theta_f)(F_f) + (\theta_i)(F_i) + (\theta_u)(F_u) \quad \text{(S4)}$$

$$F_u = \frac{K_1 K_2}{1 + K_1 + K_1 K_2} \qquad F_i = \frac{K_1}{1 + K_1 + K_1 K_2} \qquad F_f = 1 - F_i - F_u \quad \text{(S5)}$$

$$K_n = e^{-\Delta G_n / RT} \quad \text{(S6)}$$

$$\Delta G_n(T) = \Delta H_n \left(1 - \frac{T}{T_{M_n}}\right) + \Delta C_{p_n} \left[(T - T_{M_n}) - (T)\left(Ln\left(\frac{T}{T_{M_n}}\right)\right)\right] \quad \text{(S7)}$$

where K_1 and K_2 are the equilibrium constants for the transitions F→I and I→U, respectively. F_f, F_i, and F_u are the calculated fractions of the total population that the folded, intermediate, and unfolded states make up at any given temperature. The heat capacity ΔC_{p_n} for each transition is set to zero in accordance with previous analyses of CaM unfolding.[14] ΔH_n and T_{M_n} are adjustable parameters for each transition. The RMSD between the observed θ_{MRE} and θ_{fit} (equation S4) was minimized against these adjustable parameters. A weighted fraction folded value $F_f^* = F_f + nF_i$ was generated to account for the contributions of both C- and N-terminal unfolding to the overall unfolding transition. Here, n is a weighting factor held constant to $n = 0.5$ to reflect the weighted contribution of m_i in θ_{fit}. A weighted average melting temperature of the two transitions (T_M*) is generated by finding T such that $\Delta G_1(T) + \Delta G_2(T) = 0$. T_M* corresponds to the midpoint of the F_f^* unfolding curve. $\Delta G_U(25)$ for the total transition of F→U was calculated as a sum of $\Delta G_1(298.15)$ and $\Delta G_2(298.15)$. Plots for each mutant and control are shown in **Figs. S10**, **S11**, and **S12**. Comparison of the fits from the two state (F_f) and the three state (F_f*) fitting methods is shown in **Fig. S13**.

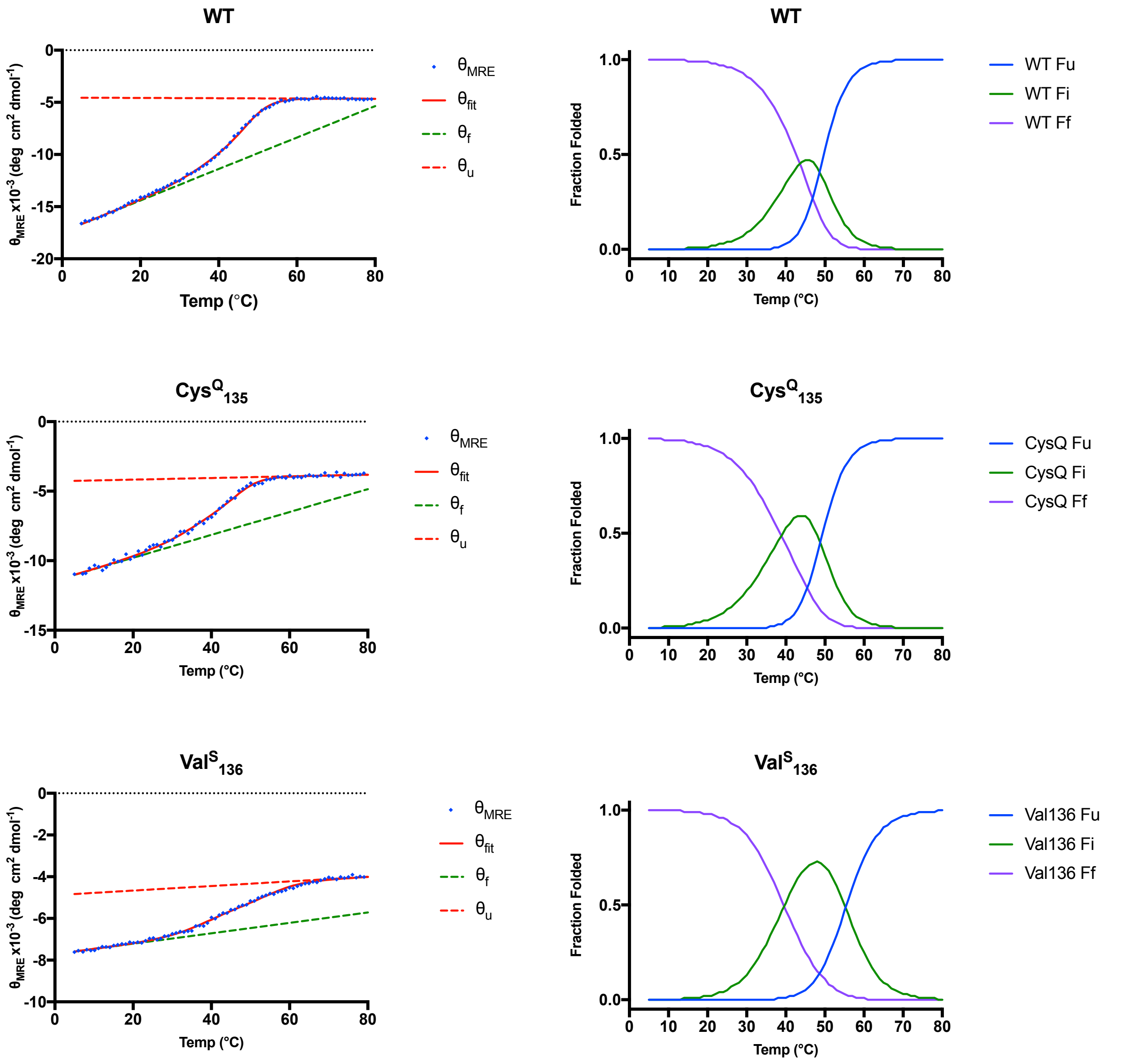

Fig. S10a. Plots generated using the three state fitting method for WT, Cys^{Q}_{135}, and Val^{S}_{136}. Left: For each CaM variant a single set of raw data (θ_{MRE}) and fits generated from θ_{fit}, θ_{f}, and θ_{u}. Right: The averaged F_f (purple), F_i (green), and F_u (blue) plots from the three replicates.

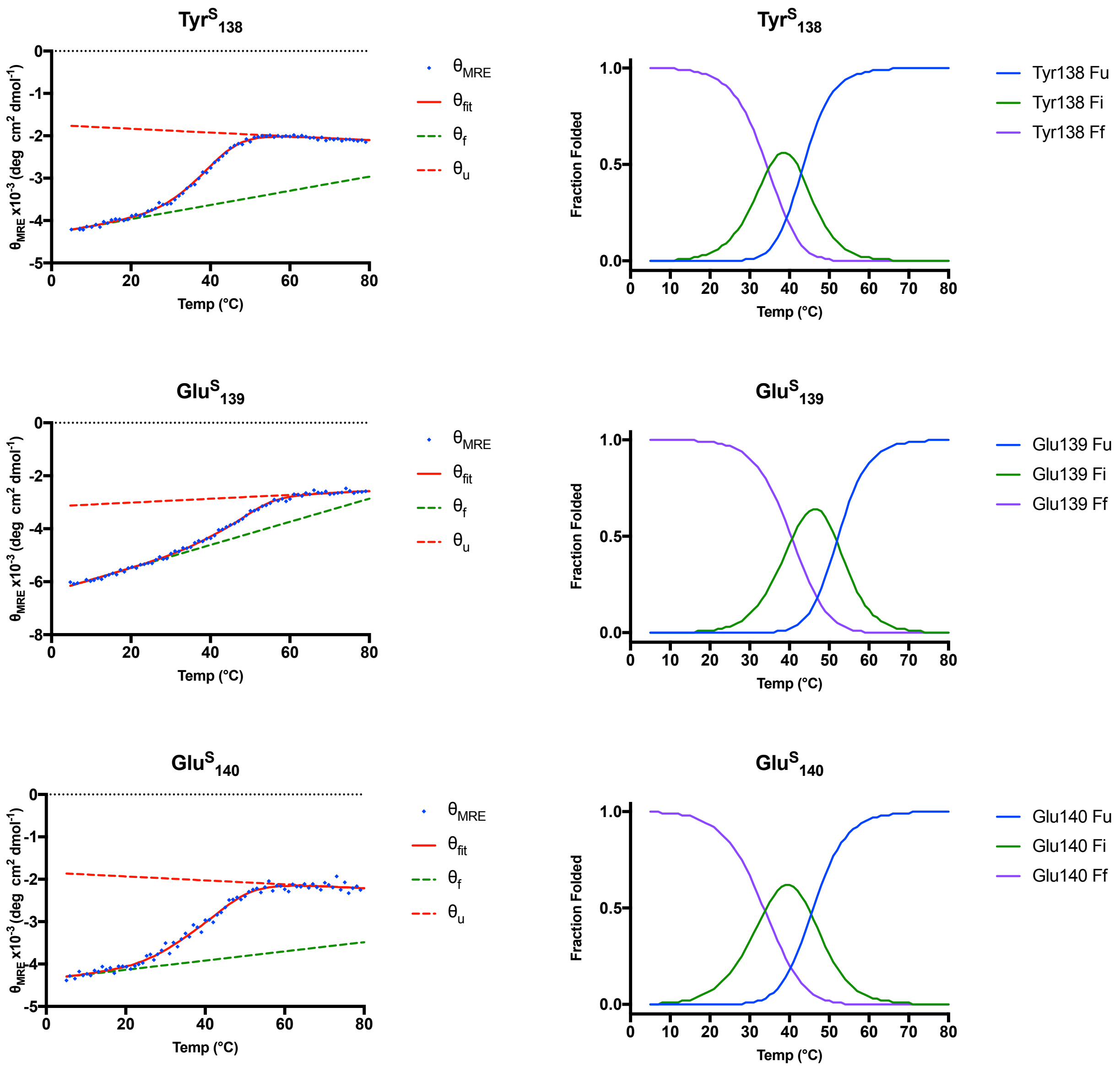

Fig. S10b. Plots generated using the three state fitting method for Tyr^{S}_{138}, Glu^{S}_{139}, and Glu^{S}_{140}. Left: For each CaM variant a single set of raw data (θ_{MRE}) and fits generated from θ_{fit}, θ_{f}, and θ_{u}. Right: The averaged F_f (purple), F_i (green), and F_u (blue) plots from the three replicates.

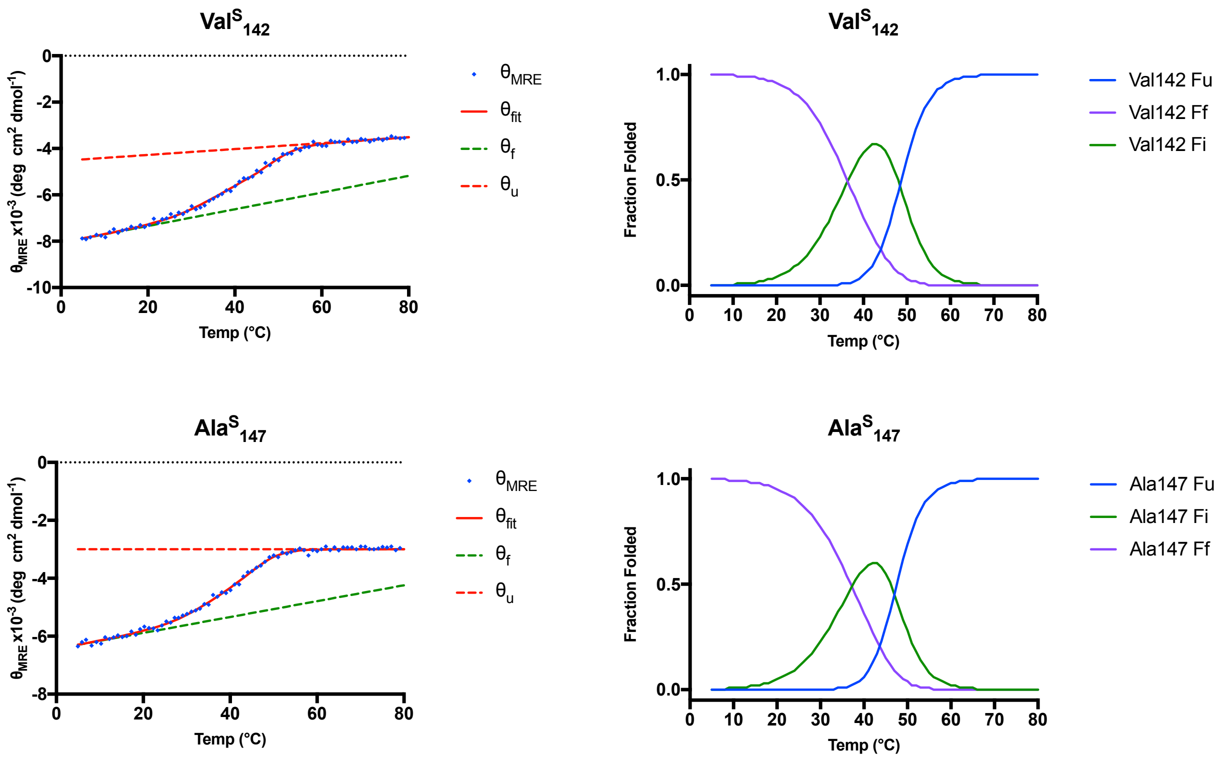

Fig. S10c: Plots generated using the three state fitting method for Val^{S}_{142} and Ala^{S}_{147}. Left: For each CaM variant a single set of raw data (θ_{MRE}) and fits generated from θ_{fit}, θ_f, and θ_u. Right: The averaged F_f (purple), F_i (green), and F_u (blue) plots from the three replicates.

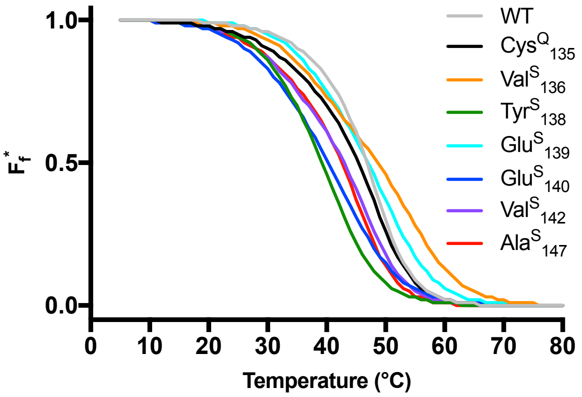

Fig. S11: F_f* plots for each CaM variant.

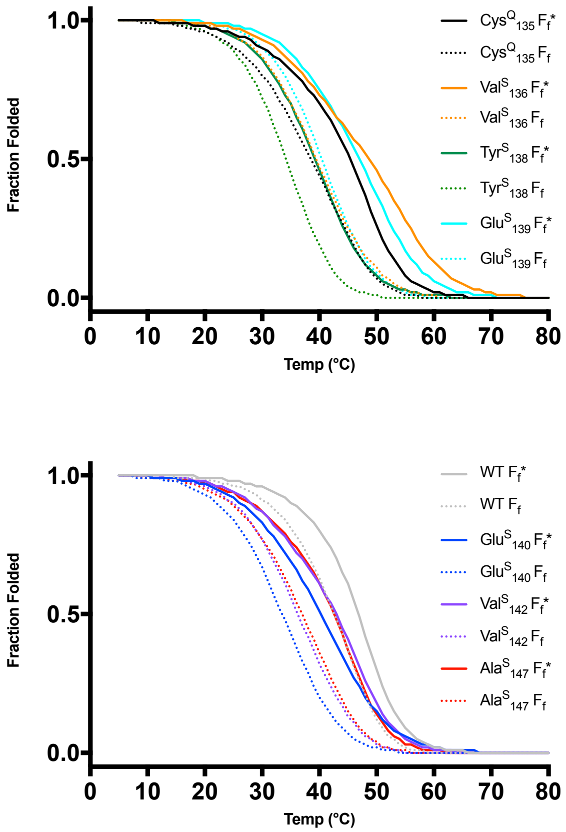

Fig. S12: Overlay of F_f (dotted lines) and F_f* (solid lines) from three state fits. Top: Cys^{Q}_{135}, Val^{S}_{136}, Tyr^{S}_{138}, and Glu^{S}_{139}. Bottom: WT, Glu^{S}_{140}, Val^{S}_{142}, and Ala^{S}_{147}.

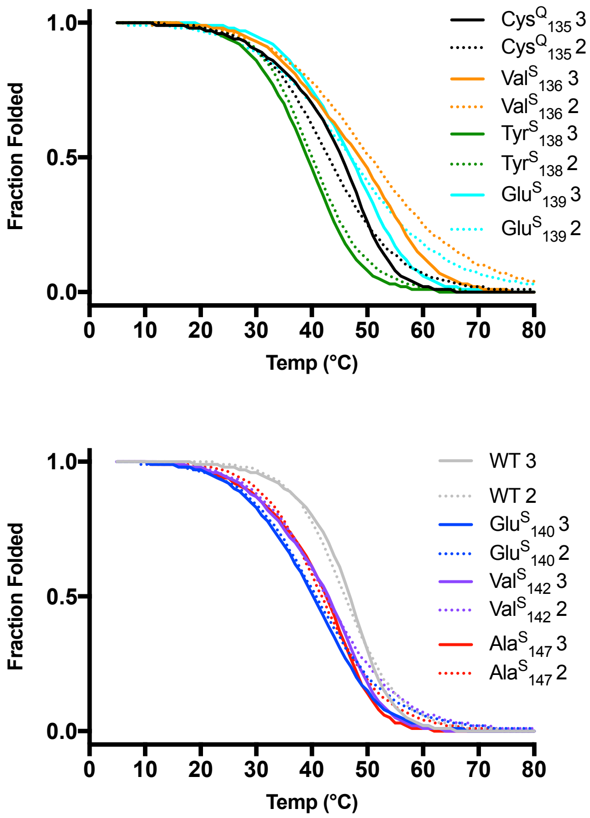

Fig. S13: Overlay of F_f (dotted lines) from two state fits and F_f^* (solid lines) from three state fits. Top: Cys^Q_{135}, Val^S_{136}, Tyr^S_{138}, and Glu^S_{139}. Bottom: WT, Glu^S_{140}, Val^S_{142}, and Ala^S_{147}.

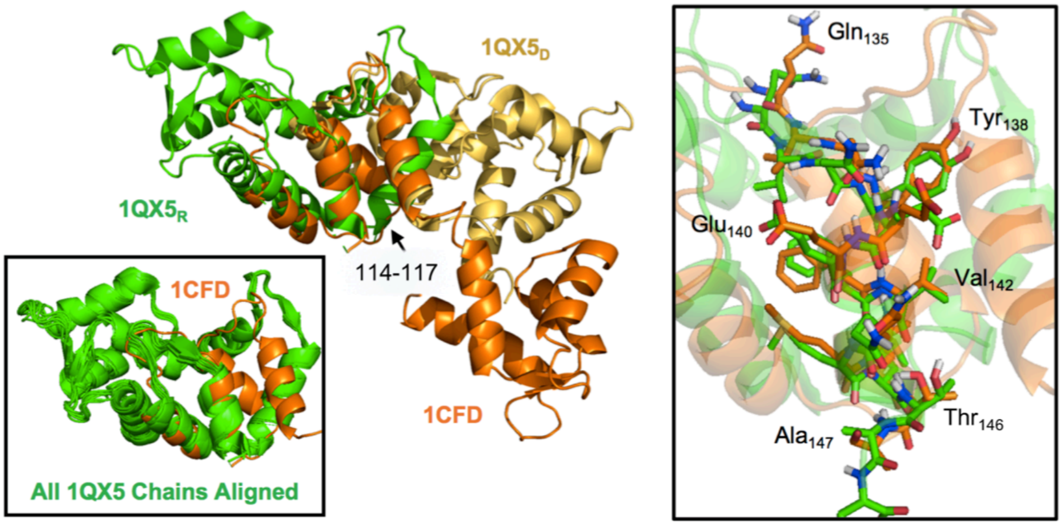

Fig. S14: Apo CaM structures. Overlay of C-terminal helix of 1CFD NMR structure with corresponding helix in chain$_R$ of 1QX5 crystal structure showing alignment of residues 118-148. Twisting about the 114-117 segment prevents simultaneous alignment of the rest of the CaM sequence. Residues 81-101 in chain$_D$ of 1QX5 structure make intermolecular contacts similar to the intramolecular contacts of residues 81-101 in 1CFD. Left Inset: All eight chains in 1QX5 structure aligned, showing identical folds. Right Inset: C-terminal helices of 1CFD and 1QX5 structures.

Table S8: Distances and angles for hydrogen bonds at sites of thioamide substitution in apo CaM structures from PDB entries 1CFD and 1QX5. 1QX5 distances are averages of distances in all 8 chains. X denotes site of thioamide insertion. Hydrogen bonding partners can be seen in Figs. S14-S16.

	C=X--N Dist. (Å)		C=X--N Angle (°)		N^X--O=C Dist. (Å)		N^X--O=C Angle (°)	
Residue	1CFD	1QX5	1CFD	1QX5	1CFD	1QX5	1CFD	1QX5
Val^S_{136}	3.4	3.1	137	151	---	---	---	---
Tyr^S_{138}	2.6	3.1	165	157	---	2.8*	---	123*
Glu^S_{139}	3.4	3.0	145	144	3.3*	---	130*	---
Glu^S_{140}	3.4	3.0	150	147	---	---	---	---
Phe^S_{141}	3.3	3.0	145	158	2.6	3.0	165	156
Val^S_{142}	3.3	3.3	160	152	3.4	3.0	150	147
Ala^S_{147}	---	---	---	---	3.4	3.2	150	115

*Asn$_{137}$ sidechain forms hydrogen bonds with either Tyr$_{138}$ or Glu$_{139}$.

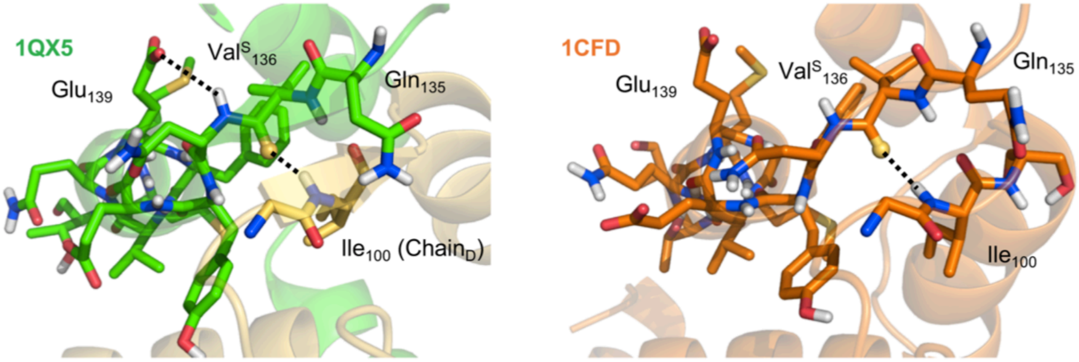

Fig. S15: Hydrogen bonding of Val$^{S}_{136}$. In the 1QX5 structure, Val$_{136}$ accepts a hydrogen bond from Ile$^{S}_{100}$ in another chain.

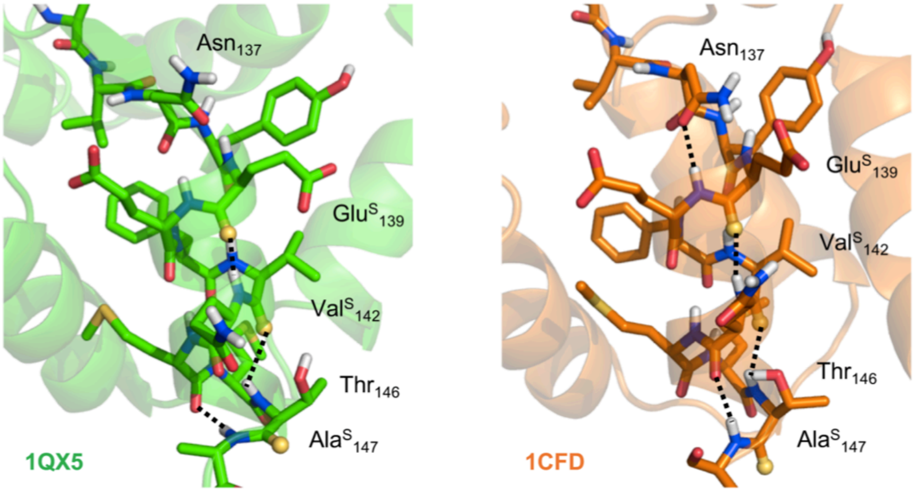

Fig. S16: Hydrogen bonding of Glu$^{S}_{139}$, Val$^{S}_{142}$, Ala$^{S}_{147}$. The 1CFD structure shows the potential stabilizing hydrogen bond from the N-H of the Glu$^{S}_{139}$ thioamide to the sidechain carbonyl of Asn$_{137}$.

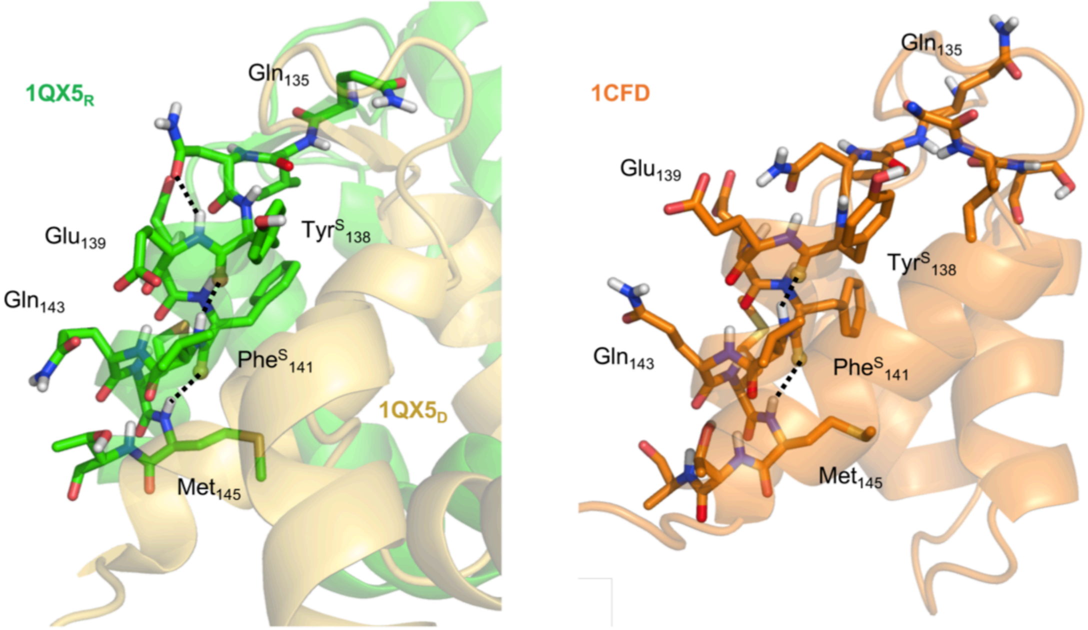

Fig. S17: Hydrogen bonding of Tyr^{S}_{138} and Phe^{S}_{141}. 1QX5 structure shows the potential stabilizing hydrogen bond from the N-H of the Tyr^{S}_{138} thioamide to the sidechain carbonyl of Asn_{137}.

GB1 Synthesis and Analysis

SPPS of Leu$^S_5$ and Ile$^S_6$ GB1 Thioproteins. Wild-type GB1 and thioamide-containing mutants were synthesized by automated methods on a PTI Tribute synthesizer (Tuscon, AZ) using NovaPEG Rink Amide resin (70 μmol scale). Coupling reactions were performed by combining 3.0 mL of 0.4 M *N*-methylmorpholine in DMF with 7 equivalents each of Fmoc-amino acid and HCTU. Following a 2 min preactivation, the solution was added to resin and vortexed for 45 min. The resin was washed with DMF (3 x 3 mL) and resuspended in DMF (3 mL). For incorporation of the thioamide, either Fmoc-Leu^S-NBt or Fmoc-Ile^S-NBt (5 equivalents) was dissolved in DMF (1 mL) and immediately added to the resin. DIPEA (10 equivalents) was added and the mixture was stirred at room temperature for 2 h. The resin was then washed thoroughly with DMF (3 x 3 mL) after each coupling reaction. Each thioamide precursor was double coupled using the modified coupling procedure. Deprotection reactions were carried out twice using a 20% v/v solution of 4-methylpiperidine in DMF (3 mL) for 4 min. The resin was washed with DMF (3 x 3 mL) for 40 sec between each cycle. After the final Fmoc deprotection reaction, the resin was washed with DCM (3 mL) followed by methanol (3 mL). Resin was dried and treated with a solution of 92% v/v TFA, 4% v/v TIPS, 4% v/v EDT for 3 h to cleave protein from resin and remove side chain protecting groups. After filtration, the crude protein was precipitated by addition of cold diethyl ether. The solid was pelleted by centrifugation and dissolved in 6 M Gdn•HCl, 25 mM sodium phosphate, pH 6 for purification.

Each protein was purified by a two-stage protocol of HPLC followed by ion exchange. Preparative reverse-phase HPLC was carried out on a C18 column using the two solvents 0.1% TFA in water and 0.1% TFA in acetonitrile. Anion-exchange chromatography was carried out on a monoQ 5/50GL column (GE Healthcare) using 20 mM Tris buffer at pH 8 and eluting with increasing concentrations of KCl. Final protein samples were ≥ 95% pure by analytical reversed-phase HPLC. The identity of each protein was confirmed by MALDI (Leu$^S_5$ $[M+H]^+$ = 6196.7, observed 6191.8; Ile$^S_6$ $[M+H]^+$ = 6196.7, observed 6191.3). Typical isolated yields of GB1 thioproteins by SPPS were 1% (0.7 μmol).

Native Chemical Ligation Synthesis of GB1 Variants

Purification of $GB1_{24\text{-}56}$ Cys_{24} (10). Crude peptide **10** was purchased from Genscript (Piscataway, NJ). Crude peptide (20 mg) was dissolved in CH_3CN/H_2O (1:1) containing 20 μL of 0.5 M TCEP bond breaker™ and purified by reverse phase HPLC (gradient **1**, **Table S9**) at a flow rate of 15 mL/min on a Grace Vydac C18 preparatory column. Individual fractions were characterized by MALDI- MS (**Table S10**), and dried by lyophilization. Dried peptide was dissolved in CH_3CN/H_2O (1:1), quantified by UV/Vis (ε_{280} = 8,480 M^{-1} cm^{-1}), aliquoted into 100 nmol portions and lyophilized for later use.

Synthesis and Purification of $GB1_{1\text{-}23}$-N_2H_3 (11). The N-terminal fragment of GB1 (**11**) was synthesized on a CEM Liberty 1 Automated Microwave Peptide Synthesizer (Matthews, NC) on a 100 μmol scale. The hydrazide resin was prepared according to Liu and coworkers[16] and the first residue was coupled manually. 20% piperidine in DMF was used as deprotection reagent, 0.5 M HBTU in DMF was used as the activating reagent, and 2 M DIPEA in *N*-methyl-2-pyrrolidone (NMP) was used as the activator base. Five molar equivalents of each amino acid were used for each coupling step. Ala_{23} was coupled twice using Method 1 (listed below). Leu_{12}, Glu_{19}, and Asp_{22} were coupled using a single coupling step as in Method 2. All other residues were coupled twice using Method 3. After synthesis was completed, a final deprotection was performed, consisting of an initial deprotection for 30 sec (35 W, 50 °C), followed by a 3 min deprotection (35 W, 50°C).
Method 1: Two consecutive couplings for 10 min each (25 W, 50°C) were used.
Method 2: Initial deprotection for 30 sec (35 W, 50°C), followed by a 3 min deprotection (35 W, 50°C). A single coupling for 10 min (25 W, 50°C) was used.
Method 3: Initial deprotection for 30 sec (35 W, 50°C), followed by a 3 min deprotection (35 W, 50°C). Two consecutive couplings for 10 min each (25 W, 50°C) were used.

Peptide cleavage from resin was performed by treating the resin with a cleavage cocktail (18:1:1 TFA:TIPS:H_2O) for 45 min. The solution was then collected by filtration, and dried by rotary evaporation. For purification, the crude residues were dissolved in CH_3CN/H_2O (1:1), diluted with H_2O, and then purified by reverse phase HPLC (gradient **2**, **Table S10**) at a flowrate of 15 mL/min on a Grace Vydac C18 preparatory column. Individual fractions were characterized by MALDI-MS (**Table S11**), and dried by lyophilization. Dried peptide was dissolved in

CH_3CN/H_2O (1:1), quantified by UV/Vis (ε_{274} = 1,400 M^{-1} cm^{-1}), aliquoted into 100 nmol aliquots, and lyophilized for later use.

Synthesis and Purification of $GB1_{1\text{-}23}$-N_2H_3 Thiopeptides (12-14). Residues 8-23 were synthesized on a peptide synthesizer on a 150 μmol scale as described for $GB1_{1\text{-}23}$-N_2H_3 (**11**). Resin was split into 3 equal parts and remaining amino acids were coupled manually. For each coupling, 5 equiv of amino acid and 5 equiv of HBTU were dissolved in DMF (2 mL), pre-activated for 1 min in the presence of 10 equiv of DIPEA, and then stirred with the resin for 30 min at room temperature. For deprotections, 2% 1,8-diazabicyclo[5.4.0]undec-7-ene (DBU) in DMF (2 mL) was stirred with the resin for 2 min. The vessel was then drained, the resin was washed, and the process was repeated twice more. For thioamide couplings, 2 equiv of either Fmoc-Ile^S-NBt or Fmoc-Leu^S-NBt were dissolved in dry DCM (1 mL), and stirred with the resin for 45 min in the presence of 2 equiv DIPEA. Upon completion of SPPS, resin was rinsed thoroughly with DCM and dried under vacuum. For cleavage, resin was treated with a cleavage cocktail (18:1:1 TFA:TIPS:H_2O) for 45 min. The solution was then collected by filtration, and dried by rotary evaporation. For purification, the crude residues were brought up in CH_3CN/H_2O (1:1), diluted with H_2O to 5% CH_3CN, and then purified by reverse phase HPLC using (gradient **2**, **Table S10**) at a flowrate of 15 mL/min on a Grace Vydac C18 preparatory column. Individual fractions were characterized by MALDI-MS (**Table S11**), and dried by lyophilization. Dried peptide was dissolved in CH_3CN/H_2O (1:1), quantified by UV/Vis (ε_{274} = 11,569 M^{-1} cm^{-1}), aliquoted into 100 nmol portions and lyophilized for later use.

Native Chemical Ligation of $GB1_{24\text{-}56}$ Cys_{24} with $GB1_{1\text{-}23}$-N_2H_3. Activation buffer (200 mM Na_2HPO_4, 6 M Gdn•HCl, pH 3.0) and ligation buffer (200 mM Na_2HPO_4, 6 M Gdn•HCl, pH 7.0) were degassed by purging with argon for 15 min. 100 nmol of the C- terminal fragment (**10**) was dissolved in 95 μL ligation buffer and 5 μL thiophenol was added. Then, 100 nmol of the N-terminal fragment (**11**, **12**, **13** or **14**) was dissolved in 90 μL activation buffer and stirred at -15 °C for 10 minutes. Next, 10 μL of a 1 M solution of $NaNO_2$ in activation buffer was added and the reaction was stirred for 15 minutes at -15 °C. After 15 minutes, the C-terminal fragment was added to the N-terminal fragment, the pH was adjusted to 7.0, and the solution stirred at room temperature overnight. Upon completion, the reaction mixture was diluted to a volume of 3 mL with H_2O,

filtered through a 0.22 μm syringe filter and purified by HPLC (gradient **3**, Table **S10**) at a flowrate of 3.5 mL/min on a Grace Vydac C18 semi-preparatory column. Individual fractions were characterized by MALDI-MS (**Table S11**), and pure fractions dried by lyophilization. Typical isolated yields for NCL reactions are 30 to 40 nmol (30-40% yield).

Desulfurization of Cys_{24}. Desulfurization buffer (6 M Gdn•HCl, 0.2 M Na_2HPO_4, 0.5 M TCEP, 0.2 M thioacetamide, pH 7.2) and water were degassed by purging with argon for 15 min. A 0.5 M solution of VA-044 was freshly prepared in degassed water. The GB1 NCL products were dissolved in 160 μL desulfurization buffer. Next, 20 μL of tBuSH and 20 μL of VA-044 solution were added and the microcentrifuge tube was put under Ar atmosphere, sealed with Parafilm and incubated in a 37 °C water bath for 10 hours. Once the reaction was complete, the tube was removed from the water bath and put on ice. The reaction was diluted with 450 μL H_2O, then 150 μL CH_3CN was added and the reaction mixture was purged with Ar for 10 minutes to remove excess tBuSH. The reaction was then diluted to 3 mL with H_2O and purified by reverse phase HPLC (gradient **4**, **Table S10**) at a flowrate of 3.5 mL/min on a Grace Vydac C18 semi-preparatory column. Individual fractions were characterized by MALDI-MS (**Table S11**), and dried by lyophilization.

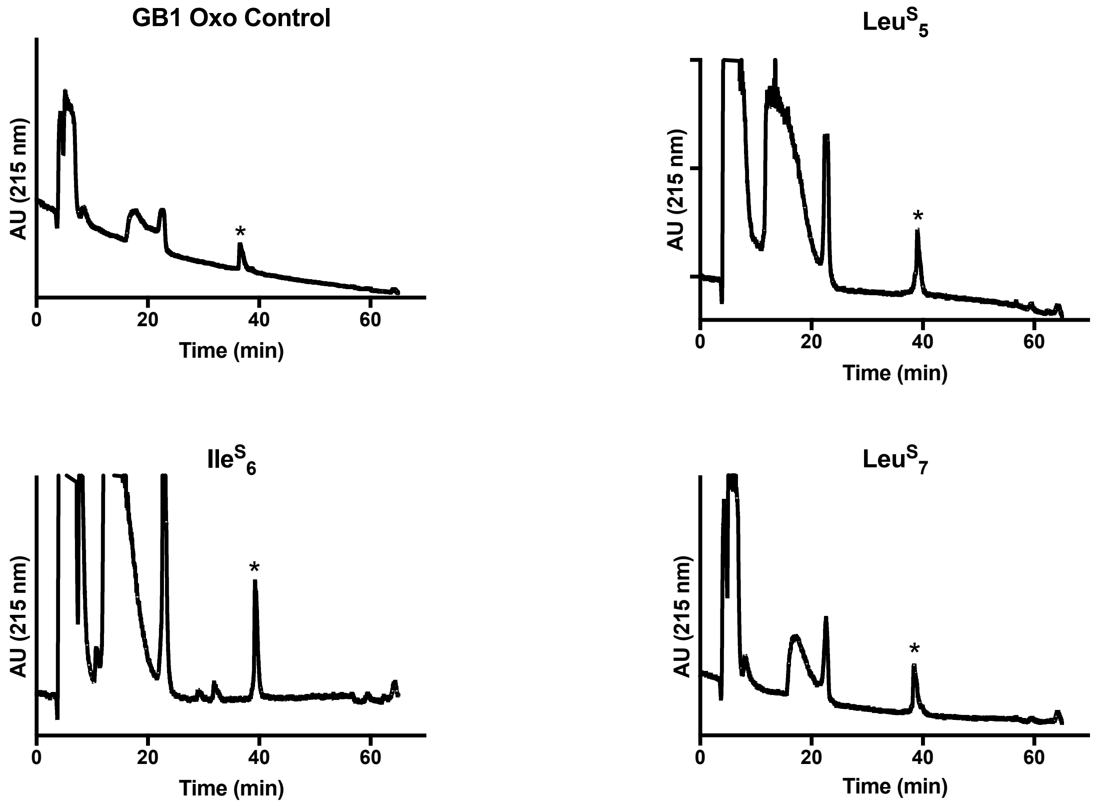

Figure S18: Preparative HPLC chromatographic traces (215 nm) of GB1 variants (Oxo control, Leu$^{S}_{5}$, Ile$^{S}_{6}$, and Leu$^{S}_{7}$) after desulfurization. High intensity peaks with elution times before 20 minutes are guanidinium salts from the desulfurization buffer. The desired products elute in a single pure peak (*) with retention times of approximately 40 minutes using the gradient **4** (**Table S10**).

Table S9: Sequences of GB1 peptides and full length proteins.

Peptide	Sequence
$GB1_{24\text{-}56}$ Cys_{24} (**10**)	CTAEKVFKQYANDNGVDGEWTYDDATKTFTVTE
$GB1_{1\text{-}23}$-N_2H_3 (**11**)	DTYKLILNGKTLKGETTTEAVDA-N_2H_3
$GB1_{1\text{-}23}$ $Leu^S{}_5$-N_2H_3 (**12**)	DTYKL^{S}ILNGKTLKGETTTEAVDA-N_2H_3
$GB1_{1\text{-}23}$ $Ile^S{}_6$-N_2H_3 (**13**)	DTYKLI^{S}LNGKTLKGETTTEAVDA-N_2H_3
$GB1_{1\text{-}23}$ $Leu^S{}_7$-N_2H_3 (**14**)	DTYKLIL^{S}NGKTLKGETTTEAVDA-N_2H_3
GB1 Cys_{24} (**15**)	DTYKLILNGKTLKGETTTEAVDACTAEKVFKQYANDNGVDGEWTYDDATKTFTVTE
GB1 $Leu^S{}_5$ Cys_{24} (**16**)	DTYKL^{S}ILNGKTLKGETTTEAVDACTAEKVFKQYANDNGVDGEWTYDDATKTFTVTE
GB1 $Ile^S{}_6$ Cys_{24} (**17**)	DTYKLI^{S}LNGKTLKGETTTEAVDACTAEKVFKQYANDNGVDGEWTYDDATKTFTVTE
GB1 $Leu^S{}_7$ Cys_{24} (**18**)	DTYKLIL^{S}NGKTLKGETTTEAVDACTAEKVFKQYANDNGVDGEWTYDDATKTFTVTE
WT Oxo GB1 (**19**)	DTYKLILNGKTLKGETTTEAVDAATAEKVFKQYANDNGVDGEWTYDDATKTFTVTE
GB1 $Leu^S{}_5$ (**20**)	DTYKL^{S}ILNGKTLKGETTTEAVDAATAEKVFKQYANDNGVDGEWTYDDATKTFTVTE
GB1 $Ile^S{}_6$ (**21**)	DTYKLI^{S}LNGKTLKGETTTEAVDAATAEKVFKQYANDNGVDGEWTYDDATKTFTVTE
GB1 $Leu^S{}_7$ (**22**)	DTYKLIL^{S}NGKTLKGETTTEAVDAATAEKVFKQYANDNGVDGEWTYDDATKTFTVTE

Table S10: HPLC gradients for the purification of GB1 peptides.

No.	Time (min)	Buffer A (%)	No.	Time (min)	Buffer A (%)
**1**	0:00	98	**2**	0:00	95
	5:00	98		5:00	95
	10:00	80		10:00	80
	30:00	70		25:00	70
	32:00	70		27:00	0
	33:00	0		30:00	0
	38:00	0		42:00	95
	39:00	95			
**3**	0:00	98	**4**	0:00	98
	15:00	98		15:00	98
	18:00	75		18:00	85
	48:00	65		48:00	55
	52:00	65		52:00	55
	53:00	0		53:00	0
	58:00	0		58:00	0
	60:00	98		60:00	98

Table S11: Calculated and observed m/z for isolated GB1 peptides.

Peptide	$[M + H]^+$		$[M + Na]^+$	
	Calculated	Observed	Calculated	Observed
$GB1_{24\text{-}56}$ Cys_{24} (**10**)	3746.68	3746.74	3768.66	3768.69
$GB1_{1\text{-}23}$-N_2H_3 (**11**)	2495.33	2495.40	2517.31	2517.41
$GB1_{1\text{-}23}$ $Leu^S{}_5$-N_2H_3 (**12**)	2511.39	2511.37	2533.37	2533.38
$GB1_{1\text{-}23}$ $Ile^S{}_6$-N_2H_3 (**13**)	2511.39	2511.30	2533.37	2533.29
$GB1_{1\text{-}23}$ $Leu^S{}_7$-N_2H_3 (**14**)	2511.39	2511.36	2533.37	2533.36
GB1 Cys_{24} (**15**)	6212.78	6212.38	---	---
GB1 $Leu^S{}_5$ Cys_{24} (**16**)	6228.84	6228.71	---	---
GB1 $Ile^S{}_6$ Cys_{24} (**17**)	6228.84	6228.23	---	---
GB1 $Leu^S{}_7$ Cys_{24} (**18**)	6228.84	6228.90	---	---
WT Oxo GB1 (**19**)	6180.72	6181.22	---	---
GB1 $Leu^S{}_5$ (**20**)	6196.78	6197.19	---	---
GB1 $Ile^S{}_6$ (**21**)	6196.78	6197.27	---	---
GB1 $Leu^S{}_7$ (**22**)	6196.78	6198.87	---	---

Table S12: Synthesized GB1 variants and their yields based upon UV-Vis quantification prior to CD measurements.

GB1 Variant	Percent Yield
Oxo Control	17.2 %
$Leu^S{}_5$	10.9 %
$Ile^S{}_6$	10.6 %
$Leu^S{}_7$	11.9 %

Sample Preparation for CD. Dried peptides were dissolved in 500 µL ligation Buffer (200 mM Na_2HPO_4, 6 M GnHCl, pH 7.0) and dialyzed against 2 L 20 mM Na_2HPO_4, pH 7.0. The dialysis buffer was exchanged for fresh buffer twice over a period of 24 hours. After dialysis, samples were concentrated to 300-400 uL, using Amicon Ultra centrifugal filter units (3 kDa MWCO).

Concentration Determination of GB1. The absorbance for each GB1 sample was measured at 274 nm and Beer's law was used to determine the concentration of each sample by using extinction

coefficients of $\varepsilon_{274} = 9567\ M^{-1}\ cm^{-1}$ and $\varepsilon_{274} = 19736\ M^{-1}\ cm^{-1}$ for the GB1 Oxo and GB1 thioamide proteins, respectively.

CD Wavelength Measurements. Each sample (300 µL) was added to a 1 mm path length Helma Analytics 110-QS CD cuvette and loaded into a Jasco J-1500 CD Spectrometer. CD wavelength scans were taken at 25 °C by scanning at a 1 nm step between 350 nm and 190 nm with a bandwidth of 1 nm and an averaging time of 8 s per measurement. Blank spectra were obtained by measuring the CD signal of the relevant buffer in each cuvette with an averaging time of 2 s. The raw signal (θ_d, mDeg) from each sample was background subtracted against the blank buffer spectrum and converted to molar residue ellipticity (θ_{MRE}) using equation S1. The resulting averaged scans for each GB1 variant are shown in **Fig. S19**.

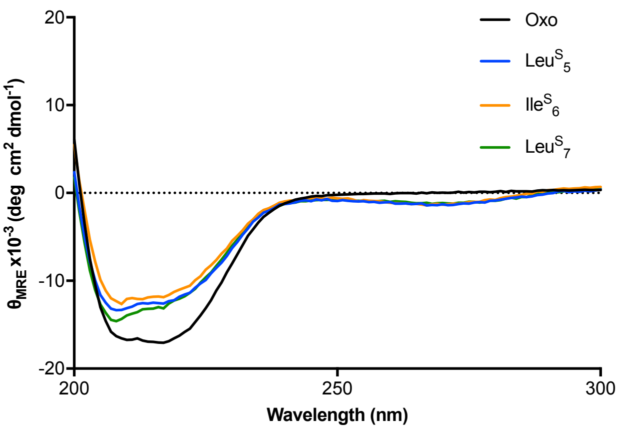

Fig. S19: CD scans of GB1 Oxo and GB1 thioproteins. Each of the thioproteins displays the characteristic minimum centered at 270 nm from the thioamide absorbance.

CD Thermal Melts. Thermal denaturation was performed by monitoring θ_d at 220 nm over a temperature range of 5 to 95 °C. The temperature increase was set to 0.2 °C per min with an equilibration time of 10 s. Measurements were made with an averaging time of 8 s per sample and a bandwidth of 1 nm.

Two-State Fitting of GB1 Thermal Melts. The two state fitting procedure described in the above CaM section was used to fit all GB1 melting profiles with a few minor changes. The linear folded and unfolded baselines for GB1 Oxo were fit from 5 to 60 °C and 90 to 95 °C, respectively. The

thioprotein folded and unfolded baselines were fit from 5 to 50 °C and 85 to 95 °C, respectively. These changes reflect the elevated melting temperature observed in GB1 compared to CaM. Changes in free energy of unfolding between GB1 Oxo and the thioprotein variants were calculated using equation S8 as described by Becktel *et al* and previously used in GB1 studies.[17,18]

$$\Delta\Delta G = \frac{\Delta T_M \cdot \Delta H_{oxo}}{T_{M,oxo}} \quad \text{(S8)}$$

Here, ΔH_{oxo} and $T_{M,\,oxo}$ are values for GB1 Oxo that are obtained from the two state fits discussed above. ΔT_M is the difference in melting temperatures between GB1 Oxo and a particular thioprotein analogue. These calculations are made under the assumption that a single amino acid change only provides a small perturbation to the system and that the structural and functional characteristics of the system largely remain intact.[17]

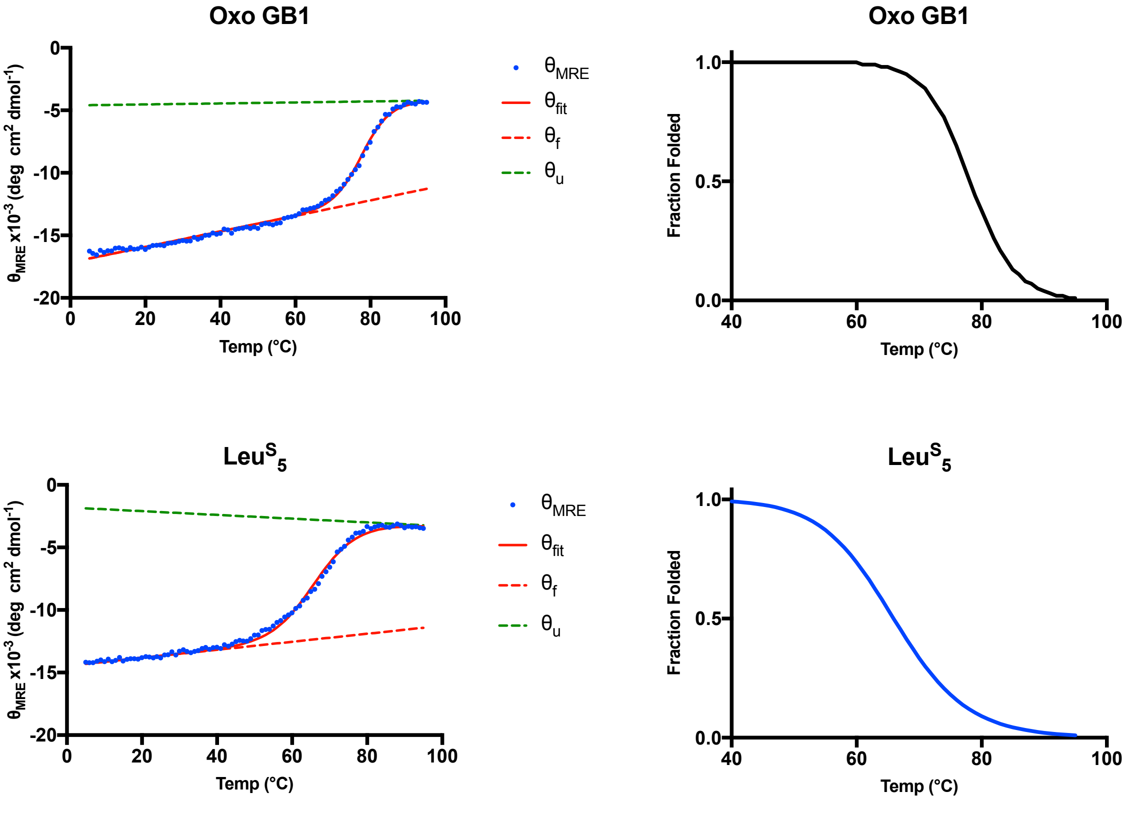

Fig. S20: Plots generated using the two state fitting method for GB1 Oxo and Leu^S_5. Left: For each GB1 variant, a single set of raw data (θ_{MRE}) and fits from equations for θ_{fit}, θ_f, and θ_u. Right: The averaged fraction folded plots (1-F_{calc}) from two replicated melts.

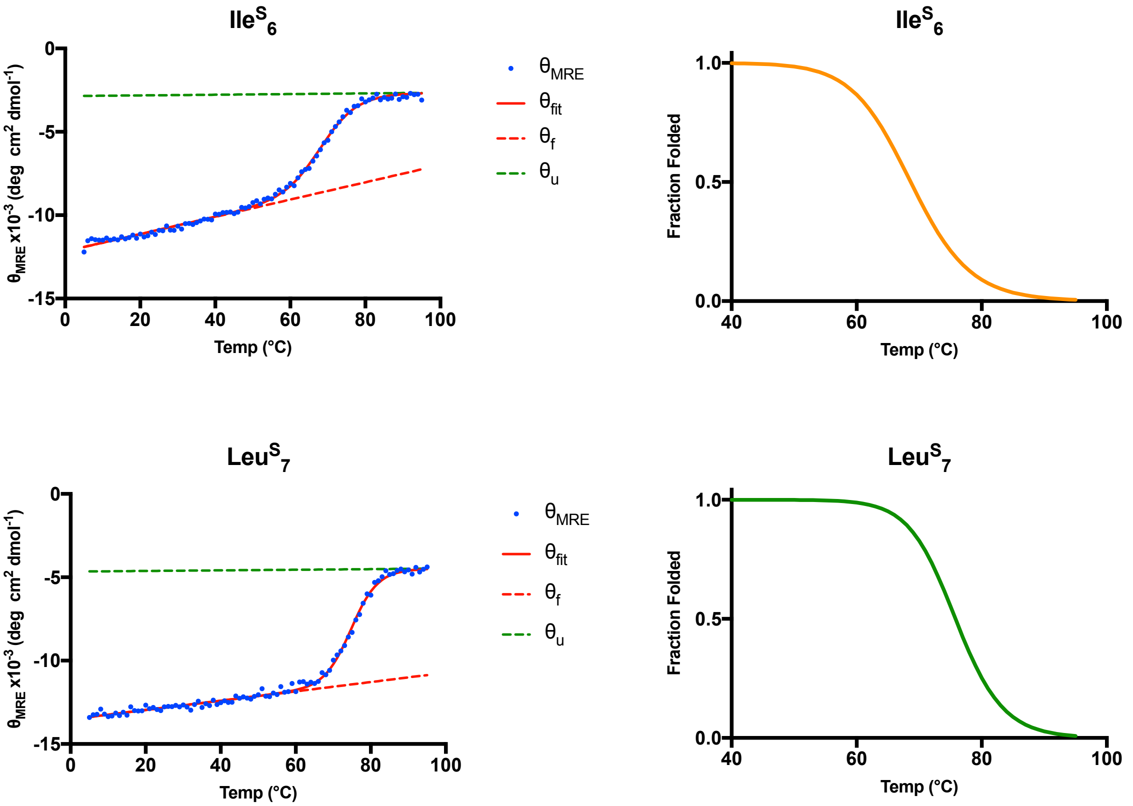

Fig. S21: Plots generated using the two state fitting method for Ile^{S}_{6} and Leu^{S}_{7}. Left: For each GB1 variant, a single set of raw data (θ_{MRE}) and fits from equations for θ_{fit}, θ_{f}, and θ_{u}. Right: The averaged fraction folded plots (1-F_{calc}) from two replicated melts.

Table S13: Distances and angles for hydrogen bonds at sites of thioamide substitution in GB1 from PDB entry 2QMT. X denotes site of thioamide insertion.

Residue	C=X--N Dist. (Å)	C=X--N Angle (º)	N^{X}--O=C Dist. (Å)	N^{X}--O=C Angle (º).
Leu^{S}_{5}	3	154	2.8	152
Ile^{S}_{6}	3	175	2.8	147
Leu^{S}_{7}	3.1	134	2.8	177

Collagen Synthesis and Analysis

Synthesis and characterization of Collagen Model Peptides (CMPs). The following series of protocols was used in the synthesis of the collagen peptides.

Protocol A – Resin Preparation followed by Fmoc-deprotection. The collagen peptides were synthesized by manual SPPS with Rink Amide AM Resin (0.62mmol/g) on a 0.02 mmol scale. Rink Amide resin (32 mg) was weighed out and transferred to a 10 ml solid phase synthesis vessel. The resin was swelled in DMF (~ 5 ml) for 30 min. The solvent was drained and the resin was washed with DMF (1 x 3 mL). A 20% piperidine in DMF solution (3 ml) was added to the resin followed by stirring at ambient temperature for 30 min. The solution was drained and the resin was washed with DMF (6 x 3 mL).

Protocol B – Trimer building block coupling followed by Fmoc-deprotection. Fmoc-Pro-Hyp(OtBu)-Gly-OH and HATU (3 eq) were dissolved in DMF (0.67 ml). Then, DIPEA (9 equiv) was added to the mixture and the mixture was incubated for another 3-5 min. This mixture was added to the reaction vessel containing the resin. The mixture was stirred for 50-80 min, drained, and washed with DMF (6 x 3 mL). The resin was immersed in 1 ml solution of 2% (v/v) DBU, 1% HOBt (m/v) in DMF for 1 minute with stirring followed by draining. This process was repeated twice more (a total of 3 doses of DBU/HOBt solution). The solution was drained and the resin was thoroughly washed with DMF (6x).

Protocol B' – Single amino acid (Aa) coupling followed by Fmoc-deprotection. Fmoc-Aa-OH (3 eq) and HATU (3 eq) were dissolved in DMF (0.67 ml). Then, DIPEA (9 eq) was added to the solution and the mixture was let stand for another 3-5 min. This mixture was added to the vessel containing amino functionalized resin. The mixture was stirred for 40-60 min, drained, and washed with DMF (6x). The resin was immersed in 1 ml solution of 2% (v/v) DBU, 1% HOBt (m/v) in DMF for 1 minute with stirring followed by draining. This process was repeated twice more (a total of 3 doses of DBU/HOBt solution). The solution was drained and the resin was thoroughly washed with DMF (6x).

Protocol B'' – Single thioamide coupling (Fmoc-Aa^{S}-NBt) followed by Fmoc-deprotection. Dry Fmoc-Aa^{S}-NBt solid (3 eq) was added to the solid phase synthesis vessel. Anhydrous DCM

(0.67 ml) was added to the vessel followed by DIPEA (1 eq). The mixture was allowed to stir at ambient temperature for 2 h, drained, and washed with DMF (6x). The resin was then immersed in 1 ml solution of 2% (v/v) DBU, 1% HOBt (m/v) in DMF for 1 minute with stirring followed by draining. This process was repeated twice more (a total of 3 doses of DBU/HOBt solution). The solution was drained and the resin was thoroughly washed with DMF (6x).

Protocol C – Acylation. A solution of DIEA (0.11 ml, 30 eq) and Ac_2O (0.06 ml, 30 eq) in DCM (3.4 ml) were added to the amino functionalized resin. This mixture was stirred for 40 min -1 h at RT. The solution was drained and thoroughly washed with DCM (5x).

Protocol D – Cleavage from the resin and collection of the crude product. The resin was suspended for 40 min in a 4 ml mixture of TFA:H_2O:TIPS (95:2.5:2.5) at RT. The filtrate was collected and dropwise added to cold diethyl ether (~11ml). The sample was incubated at 4 °C for 1 h and a white solid precipitated. The resulting sample was centrifuged and the supernatant was decanted. The white solid was dissolved in Milli-Q water and stored at -80 °C before HPLC purification.

Protocol E – HPLC purification. Acetonitrile (Buffer B) and water containing 1% TFA (Buffer A) were used as eluents. The flow rate used for semipreparitive HPLC was 4 ml/min and 1 ml/min for analytical HPLC. Crude sample was heated to >65 °C for ~10 min before injecting to prevent early triple helix formation. A Phenomenx Luna (5u C18(2) 100A; 50 x 4.60 mm, 5 µm) (Torrance, CA) column was used for purification on a Jasco HPLC instrument.

Synthesis and characterization of control peptides Ac-$(POG)_7$-NH_2 and Ac-$(POG)_3$(PPG)$(POG)_3$-NH_2 were described in a previous publication.[19] All collagen thiopeptide syntheses are described below, with validation by MALDI shown in **Table S14.**

CMP 23 Ac-$(POG)_3$(PP^SG)$(POG)_3$-NH_2

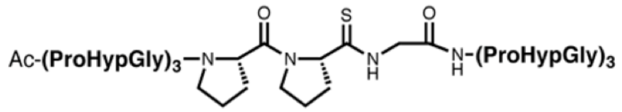

Synthesis: A + B(POG) + B (POG) + B(POG) + B'(G) + B''(Fmoc-Pro^S-NBt) + B'(P) + B(POG) + B(POG) + B(POG) + C + D + E

Note: "POG " refers to the trimer building block Fmoc-Pro-Hyp(tBu)-Gly-OH. Its synthesis was previously described by Moroder *et al.*[20]

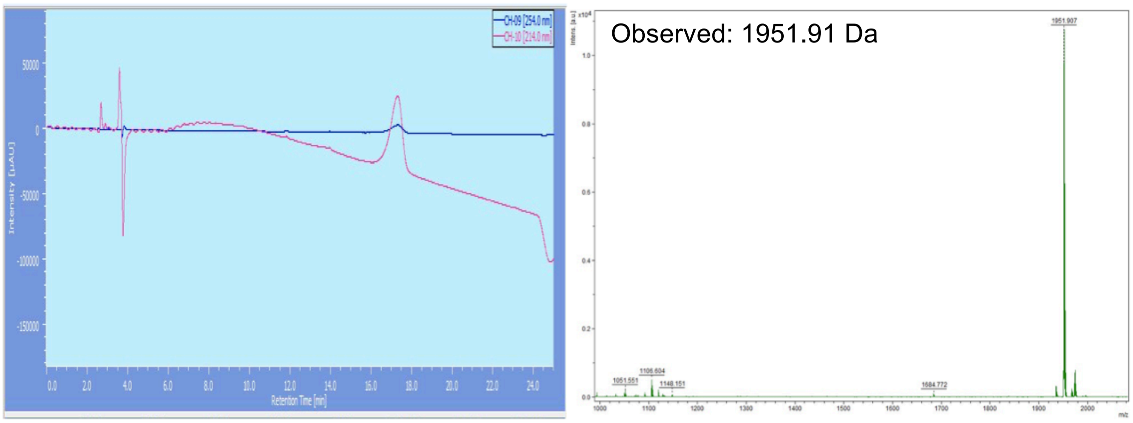

Figure S22: HPLC purification (left) and MALDI MS spectra (right) showing pure **CMP 23**.

CMP 24 Ac-$(POG)_3(P^SPG)(POG)_3$-NH_2

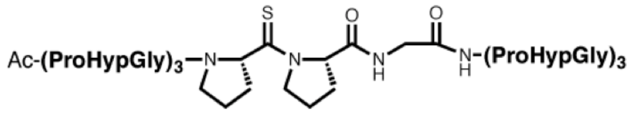

Synthesis: A + B(POG) + B (POG) + B(POG) + B'(G) + B'(P) + B''(Fmoc-Pro^S-NBt) + B(POG) + B(POG) + B(POG) + C + D + E

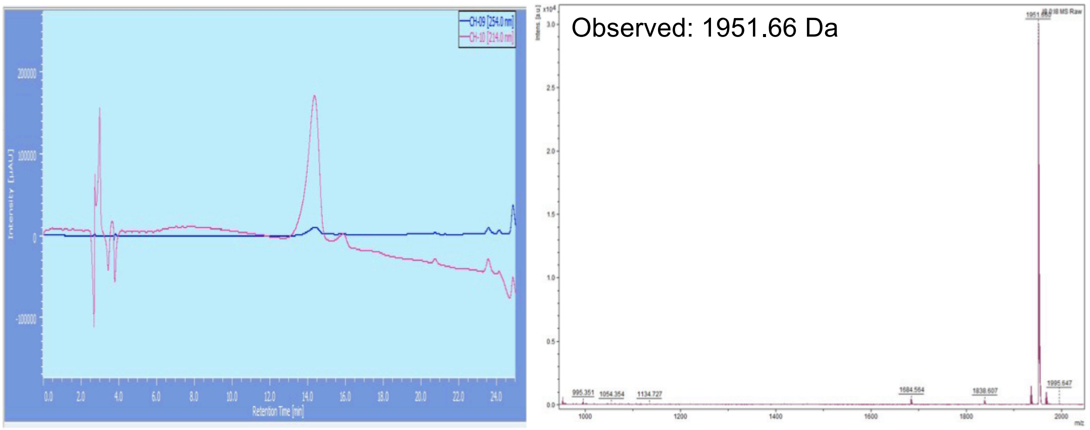

Figure S23: HPLC purification (left) and MALDI MS spectra (right) showing pure **CMP 24**.

CMP 25 Ac-(POG)$_3$(PO^{S}G)(POG)$_3$-NH$_2$

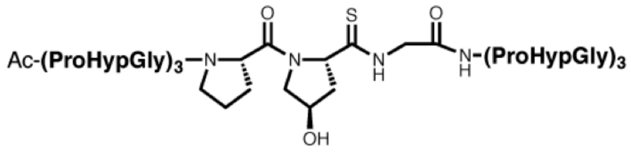

Synthesis: A + B(POG) + B (POG) + B(POG) + B'(G) + B''(Fmoc-Hyp^S(tBu)-NBt) + B'(P) + B(POG) + B(POG) + B(POG) + C + D + E

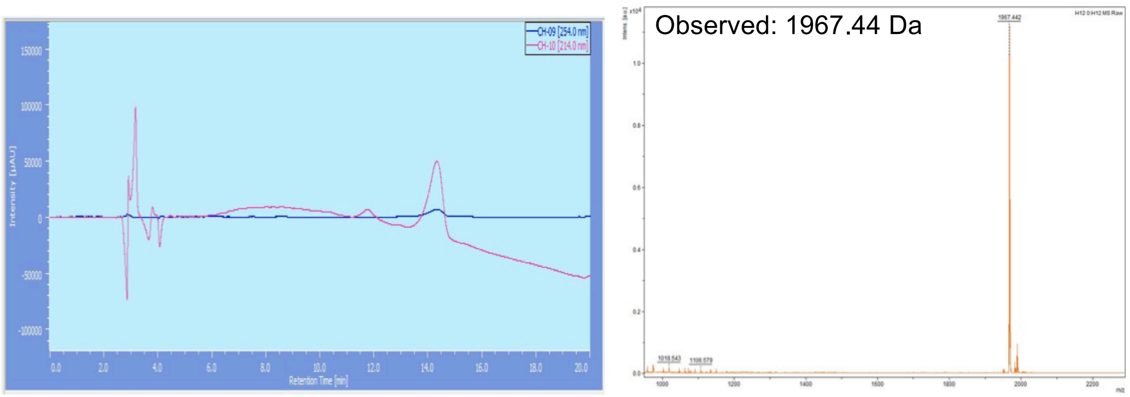

Figure S24: HPLC purification (left) and MALDI MS spectra (right) showing pure **CMP 25**.

CMP 26 Ac-$(POG)_3(PPG^S)(POG)_3$-NH_2

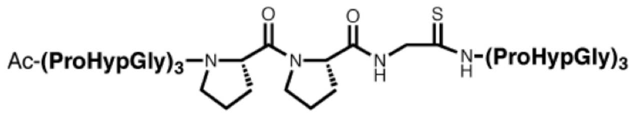

Synthesis: A + B(POG) + B (POG) + B(POG) + B’’(Fmoc-Gly^S-NBt) + B’(P) + B’(P) + B(POG) + B(POG) + B(POG) + C + D + E

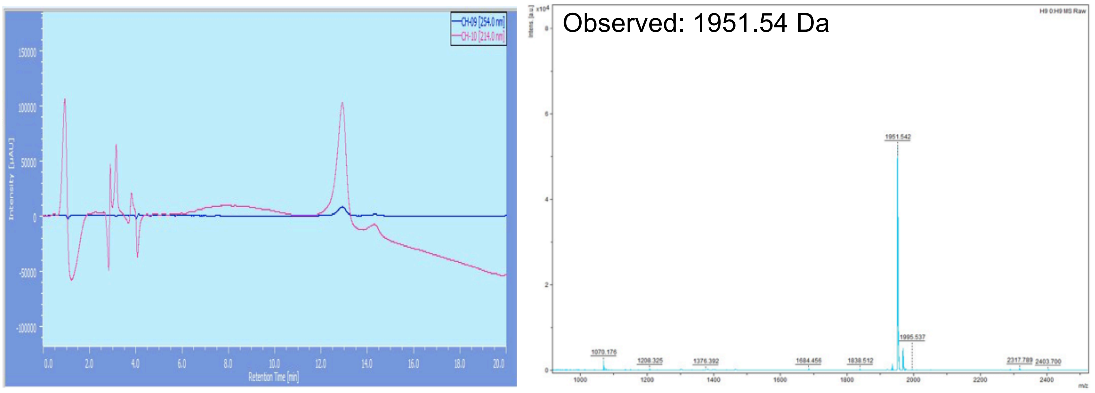

Figure S25: HPLC purification (left) and MALDI MS spectra (right) showing pure **CMP 26**.

CMP 27 Ac-$(POG)_3(POG^S)(POG)_3$-NH_2

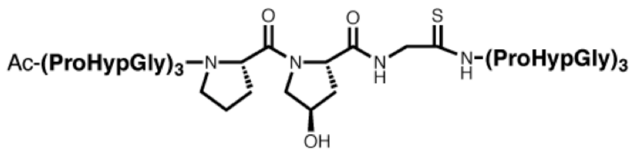

Synthesis: A + B(POG) + B (POG) + B(POG) + B''(Fmoc-Gly^S-NBt) + B'(O) + B'(P) + B(POG) + B(POG) + B(POG) + C + D + E

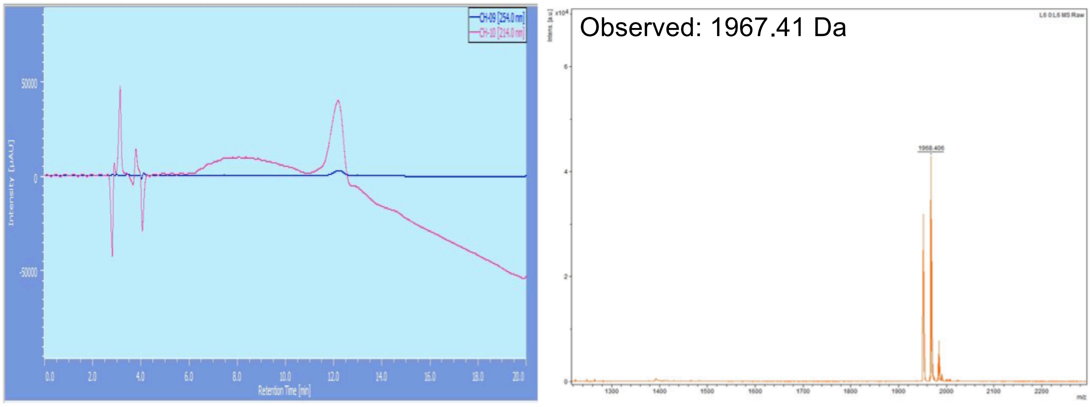

Figure S26: HPLC purification (left) and MALDI MS spectra (right) showing pure **CMP 27**.

Table S14. Calculated and observed m/z by MALDI-MS for all collagen peptides.

Collagen Variant	Calculated m/z $[M+Na]^+$	Observed m/z	Percent Yield after HPLC
Ac-$(POG)_3(PP^SG)(POG)_3$-NH_2	1951.86	1951.91	4%
Ac-$(POG)_3(P^SPG)(POG)_3$-NH_2	1951.86	1951.66	4%
Ac-$(POG)_3(PO^SG)(POG)_3$-NH_2	1967.86	1967.44	6%
Ac-$(POG)_3(PPG^S)(POG)_3$-NH_2	1951.86	1951.54	4%
Ac-$(POG)_3(POG^S)(POG)_3$-NH_2	1967.86	1967.41	5%

Peptide sample preparation. After HPLC purification, peptides were dried by lyophilization. The dried samples were dissolved in PBS buffer (0.20 g KCl, 0.20 g KH_2PO_4, 8.0 g NaCl, 2.16 g Na_2HPO_4•$7H_2O$ in 1.0 L Milli-Q H_2O) and the concentration of the stock solution was determined by a UV-Vis measurement (Jasco V-650 spectrophotometer) at 214 nm by applying $\varepsilon = 6.0 \times 10^4$ $M^{-1}cm^{-1}$ as the extinction coefficient as reported previously.[21] Solutions of peptides used in this study were then diluted to 0.2 mM final concentration using the same buffer. Samples were incubated at 4 °C for at least 24 h before CD experiments.

CD Wavelength Scans. Peptide samples (0.2 mM concentration in PBS buffer) were used for each experiment. CD spectra were recorded at a step of 1.0 nm from 390 nm to 190 nm at 10 °C with a 1.0 s equilibration time. Initial units of millidegrees were converted to θ_{MRE} before plotting.

CD Thermal Denaturation. Samples at 0.2 mM concentration in PBS buffer were used. The wavelength with the highest absorption (224 nm or 225 nm) was monitored as a function of temperature in the thermal denaturation experiment. Averaging time was set to 15 s with an equilibration time period of 2 min and θ_d monitored every 1 °C over a total range of 0 to 70 °C (overall average heating rate is 12 °C/h). Collected data from the experiments were fitted to a two-state model according to Engel *et. al.*[22] in order to obtain a T_M (temperature at which 50% of the triple helix unfolds). We used the software Scientist 3.0 (Micromath Scientific Software®, St. Louis, MO) for plotting the resulting data and followed the procedure described by Erdmann & Wennemers.[23] Plots are shown in **Figs. S27-S36**.

CD Reverse folding kinetics experiment. The peptide solution (0.2 mM) was heated at 80 °C for 15 min and transferred to a quartz cuvette (pre-cooled at 4 °C.) After 1 min, the ellipticity at 224 nm was monitored at 4 °C for 3 h, with a 10 s time constant and 5 s time interval. Fraction refolded (F_R) was calculated using equation S9.[11]

$$F_R = \frac{\theta_t - \theta_I}{\theta_N - \theta_I} \quad \text{(S9)}$$

Here, $\theta_t = \theta_{MRE}$ at time t, $\theta_N = \theta_{MRE}$ before denaturation, and θ_I = first θ_{MRE} reading (initial) recorded after ~65 s cooling (dead time). Plot of fraction refolded data versus time were then fitted to a 3rd order kinetic equation as previously reported.[24] Half-refolding time ($t_{1/2}$) was then

obtained as the time value at which 50% of peptide recovered triple helicity. At least 3 measurements and fitting process were performed on each peptide in this study.

CD Hysteresis. The wavelength with the highest absorption (224 nm or 225 nm) was monitored as a function of temperature in this unfolding/refolding experiment. The averaging time was set to be 1 s with an average heating rate of 6 °C / h. The ellipticity of every 0.1 °C was recorded from 7 °C to 57 °C and then from 57 °C to 7 °C. The data obtained from these experiments was fitted using the software Scientist 3.0 according to Model 3 developed and reported by Engel & Bächinger[25], and recently applied by Erdmann & Wennemers.[23, 26] Initial values for parameters DEN, DEU, REFN and REFU were extrapolated via trend line tool in Microsoft Excel. The activation energy was fixed to be 535,000 J mol^{-1} during the fitting process.[23, 25, 26]

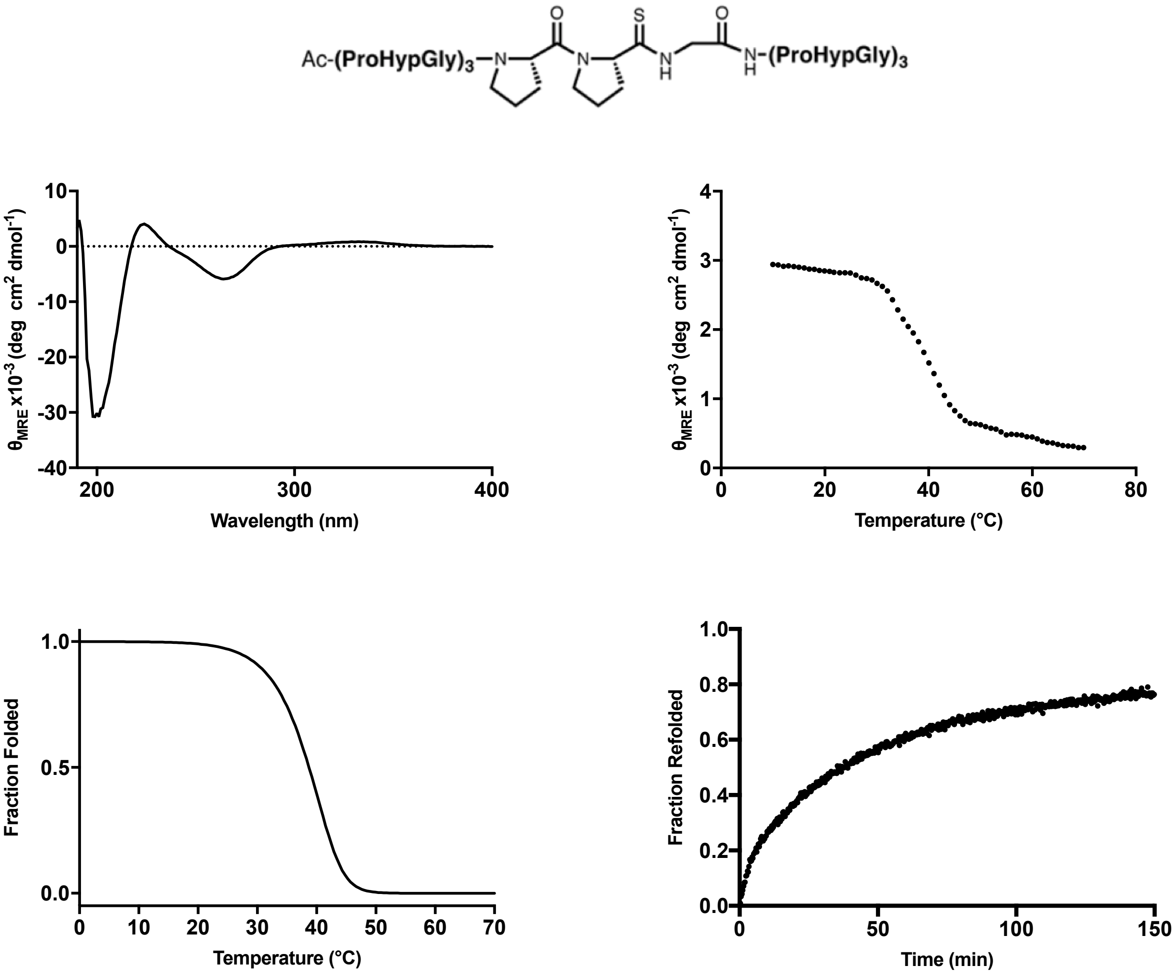

Fig. S27: CD plots for **CMP 23**: Ac-(Pro-Hyp-Gly)$_3$(Pro-Pro^S-Gly)(Pro-Hyp-Gly)$_3$-NH_2. Top middle: Chemical structure of **CMP 23**. Top left: CD wavelength scan at 10 °C. Top right: CD thermal denaturation plot at 224 nm. Bottom left: Fitted fraction folded from thermal denaturation experiment. Bottom right: Fraction refolding kinetics after denaturation.

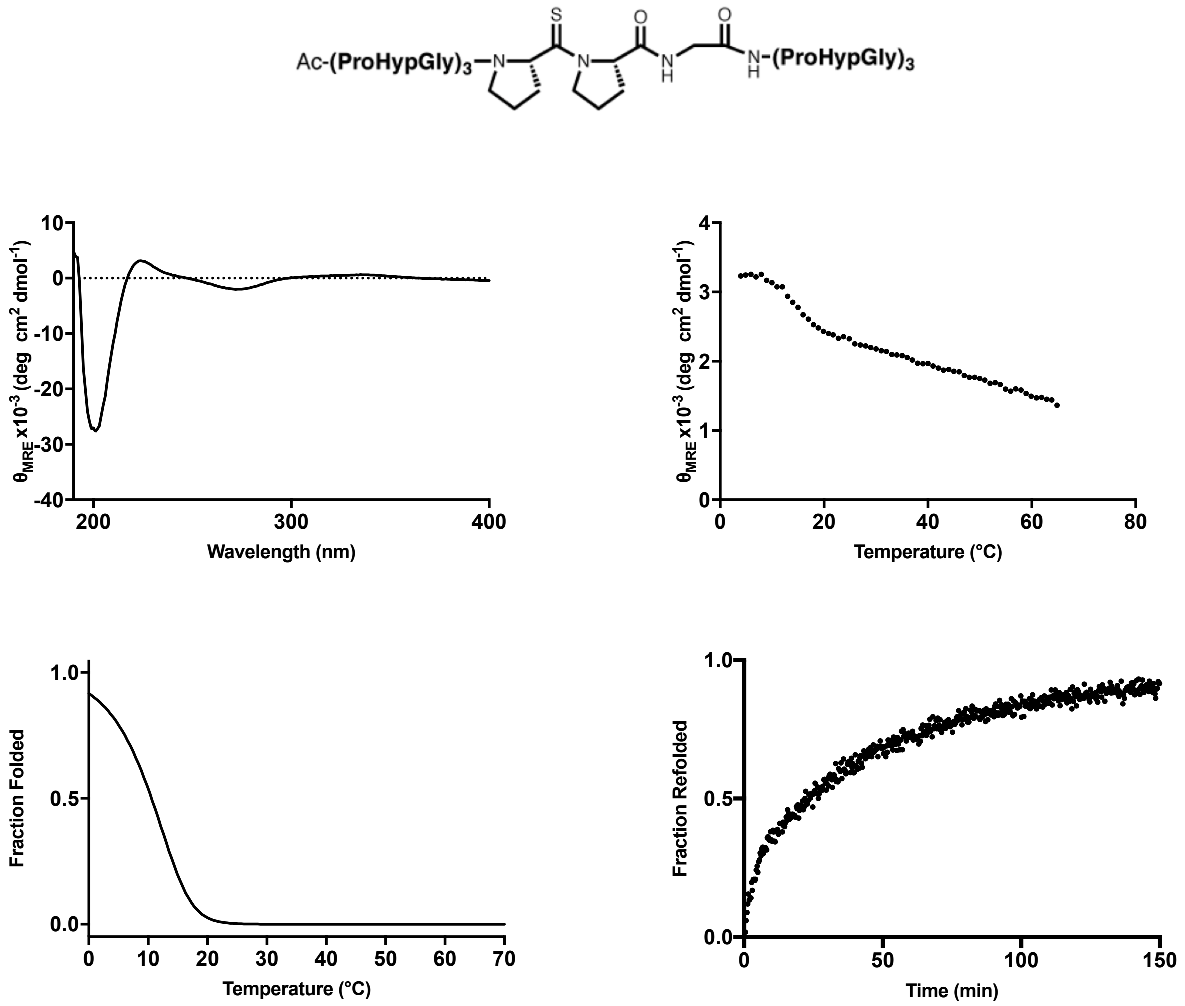

Fig. S28: CD plots for **CMP 24**: Ac-(Pro-Hyp-Gly)$_3$(Pro^S-Pro-Gly)(Pro-Hyp-Gly)$_3$-NH$_2$. Top middle: Chemical structure of **CMP 24**. Top left: CD wavelength scan at 10 °C. Top right: CD thermal denaturation plot at 224 nm. Bottom left: Fitted fraction folded from thermal denaturation experiment. Bottom right: Fraction refolding kinetics after denaturation.

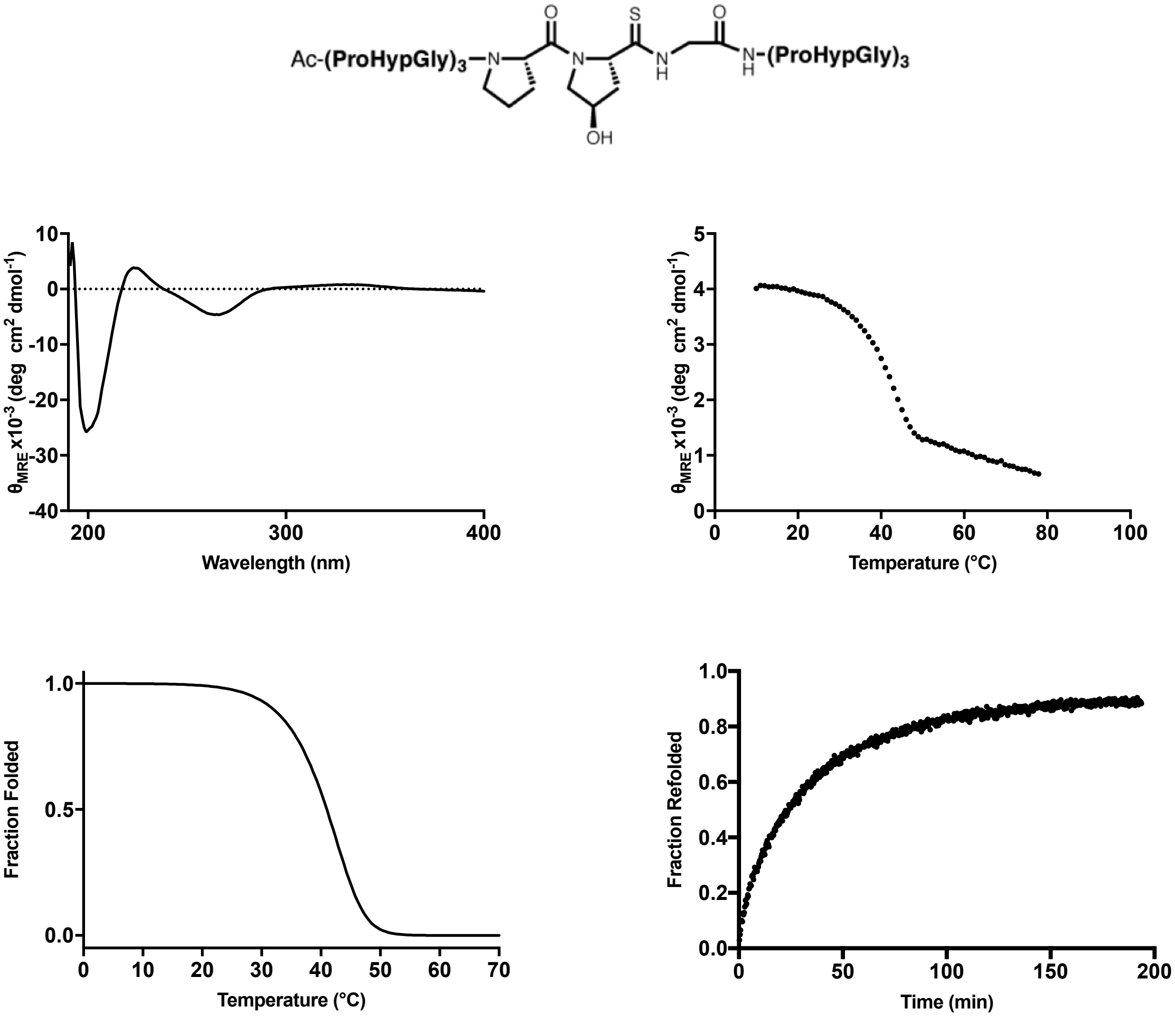

Fig. S29: CD plots for **CMP 25**: Ac-(Pro-Hyp-Gly)$_3$(Pro-Hyp^S-Gly)(Pro-Hyp-Gly)$_3$-NH$_2$. Top middle: Chemical structure of **CMP 25**. Top left: CD wavelength scan at 10 °C. Top right: CD thermal denaturation plot at 224 nm. Bottom left: Fitted fraction folded from thermal denaturation experiment. Bottom right: Fraction refolding kinetics after denaturation.

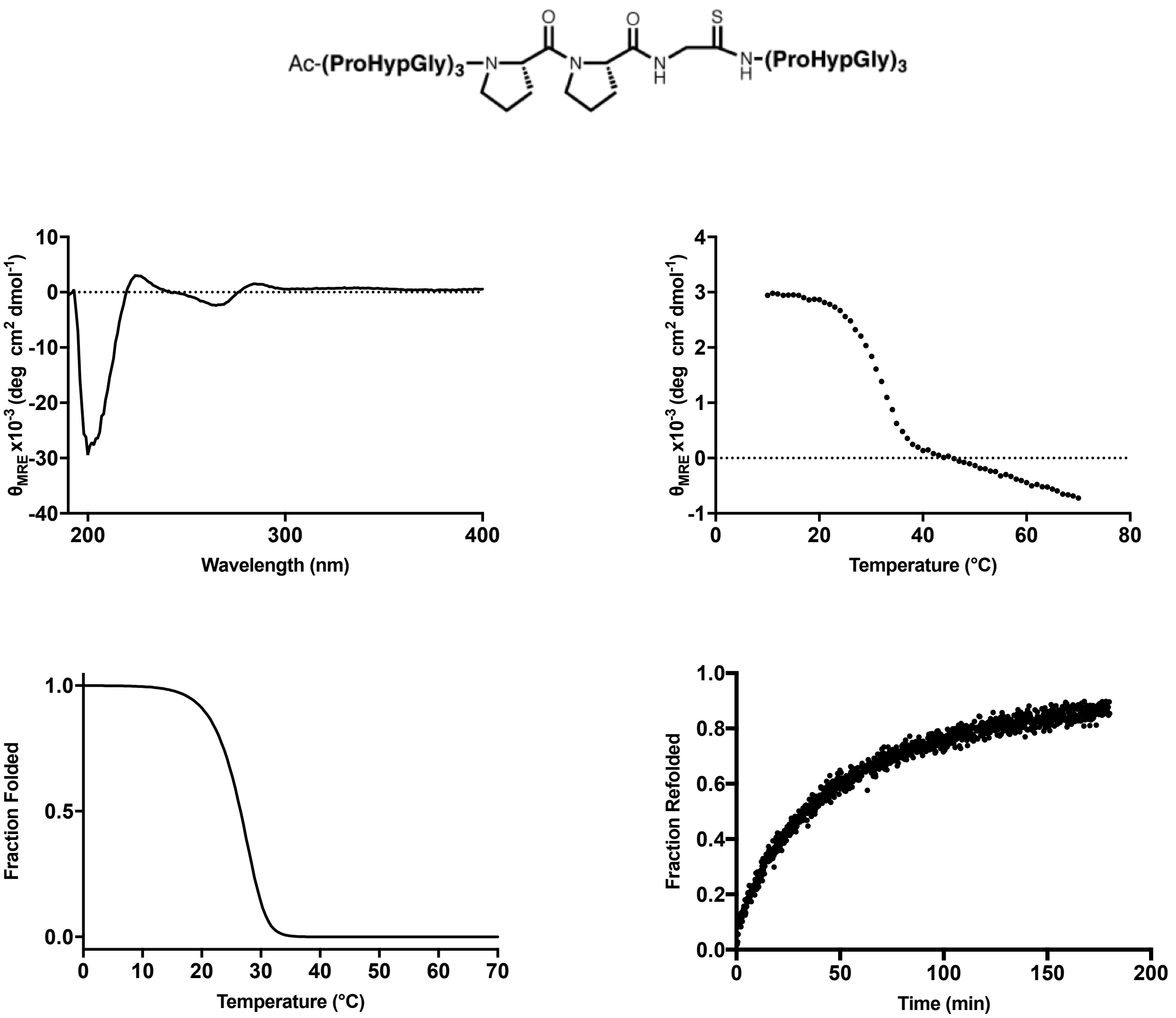

Fig. S30: CD plots for **CMP 26**: Ac-(Pro-Hyp-Gly)$_3$(Pro-Pro-Gly^S)(Pro-Hyp-Gly)$_3$-NH$_2$. Top middle: Chemical structure of **CMP 26**. Top left: CD wavelength scan at 10 °C. Top right: CD thermal denaturation plot at 224 nm. Bottom left: Fitted fraction folded from thermal denaturation experiment. Bottom right: Fraction refolding kinetics after denaturation.

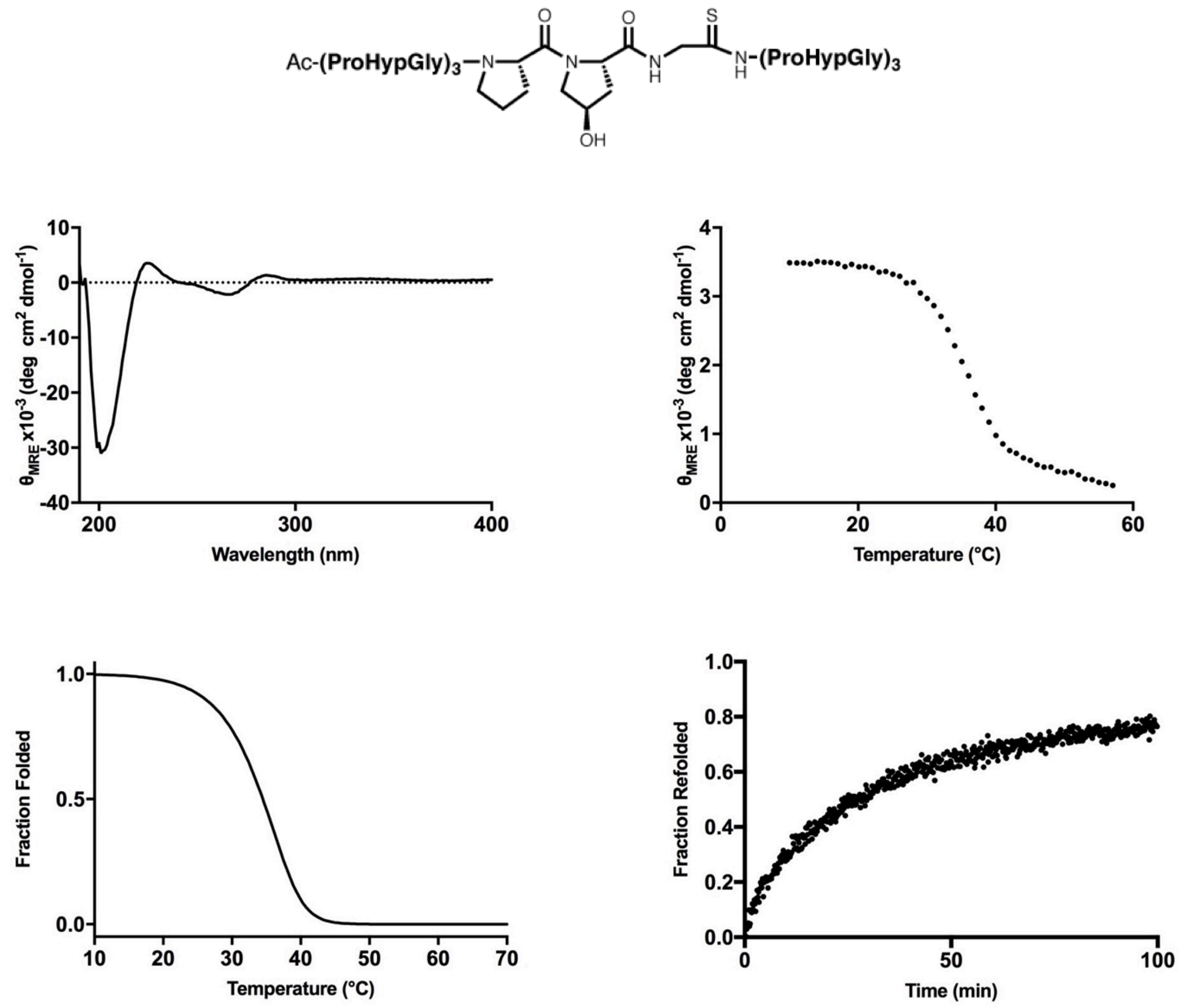

Fig. S31: CD plots for **CMP 27**: Ac-(Pro-Hyp-Gly)$_3$(Pro-Hyp-Gly^S)(Pro-Hyp-Gly)$_3$-NH$_2$. Top middle: Chemical structure of **CMP 27**. Top left: CD wavelength scan at 10 °C. Top right: CD thermal denaturation plot at 224 nm. Bottom left: Fitted fraction folded from thermal denaturation experiment. Bottom right: Fraction refolding kinetics after denaturation.

CMP 23 Ac-$(POG)_3(PP^SG)(POG)_3$-NH_2

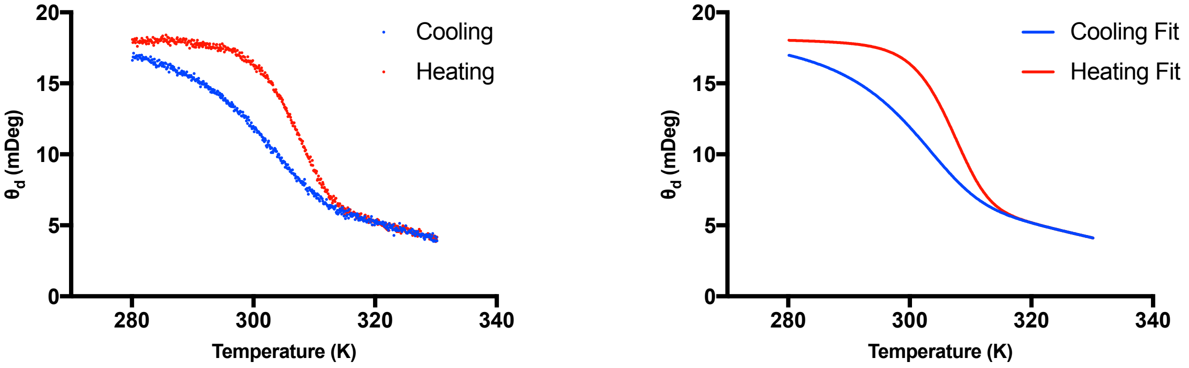

ΔH (kcal mol^{-1})	-TΔS (kcal mol^{-1})	ΔG (kcal mol^{-1}) at 298 K
-69.7	58.5	-11.2

Fig. S32: Hysteresis plot for **CMP 23.** Left: Raw CD signal at 224 nm. Right: Fits generated from the raw data. ΔH and ΔS are calculated from the fits.

CMP 24 Ac-$(POG)_3(P^SPG)(POG)_3$-NH_2

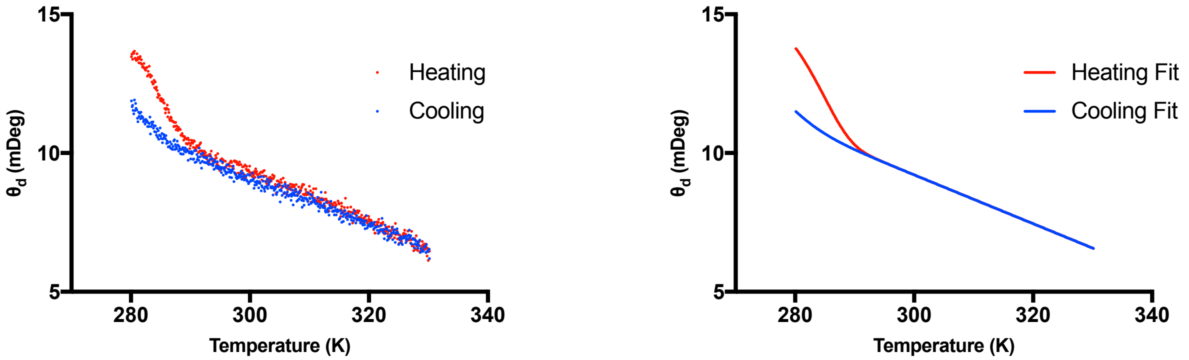

ΔH (kcal mol^{-1})	-TΔS (kcal mol^{-1})	ΔG (kcal mol^{-1}) at 298 K
-70.6	65.4	-5.2

Fig. S33: Hysteresis plot for **CMP 24.** Left: Raw CD signal at 224 nm. Right: Fits generated from the raw data. ΔH and ΔS are calculated from the fits.

CMP 25 Ac-(POG)$_3$(PO^{S}G)(POG)$_3$-NH$_2$

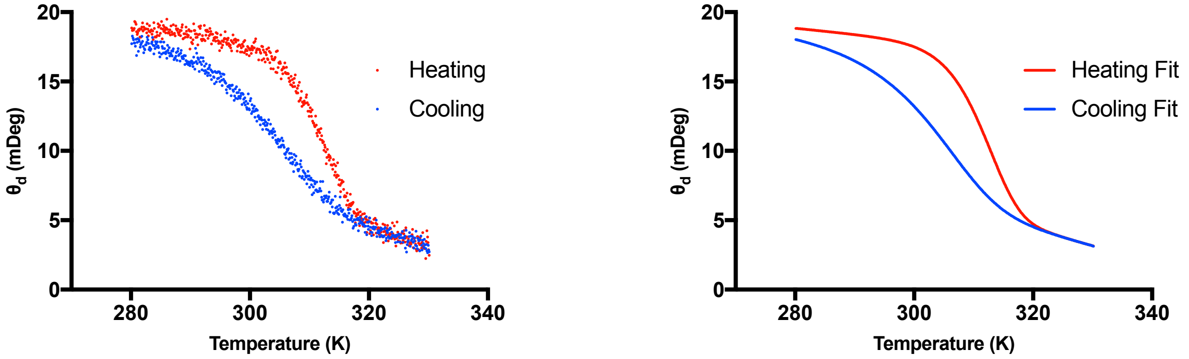

ΔH (kcal mol^{-1})	-TΔS (kcal mol^{-1})	ΔG (kcal mol^{-1}) at 298 K
-75.9	63.6	-12.3

Fig. S34: Hysteresis plot for **CMP 25.** Left: Raw CD signal at 224 nm. Right: Fits generated from the raw data. ΔH and ΔS are calculated from the fits.

CMP 26 Ac-(POG)$_3$(PPG^S)(POG)$_3$-NH$_2$

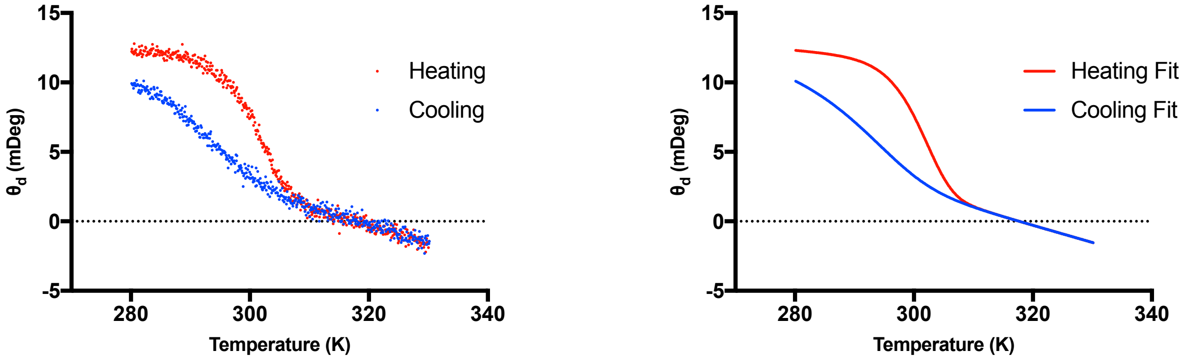

ΔH (kcal mol^{-1})	-TΔS (kcal mol^{-1})	ΔG (kcal mol^{-1}) at 298 K
-68.6	59.2	-9.3

Fig. S35: Hysteresis plot for **CMP 26.** Left: Raw CD signal at 224 nm. Right: Fits generated from the raw data. ΔH and ΔS are calculated from the fits.

CMP 27 Ac-$(POG)_3(POG^S)(POG)_3$-NH_2

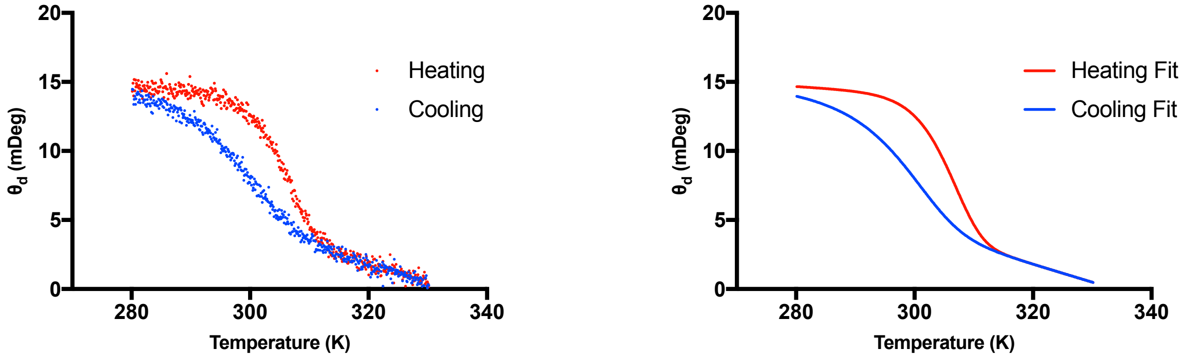

ΔH (kcal mol^{-1})	-TΔS (kcal mol^{-1})	ΔG (kcal mol^{-1}) at 298 K
-85.0	73.9	-11.1

Fig. S36: Hysteresis plot for **CMP 27.** Left: Raw CD signal at 224 nm. Right: Fits generated from the raw data. ΔH and ΔS are calculated from the fits.

References

1. M. A. Shalaby, C. W. Grote and H. Rapoport, *J. Org. Chem.*, 1996, **61**, 9045-9048.
2. S. Batjargal, Y. Huang, Y. X. J. Wang and E. J. Petersson, *J. Pept. Sci.*, 2014, **20**, 87-91.
3. S. Batjargal, Y. J. Wang, J. M. Goldberg, R. F. Wissner and E. J. Petersson, *J. Am. Chem. Soc.*, 2012, **134**, 9172-9182.
4. J. M. Goldberg, S. Batjargal, B. S. Chen and E. J. Petersson, *J. Am. Chem. Soc.*, 2013, **135**, 18651-18658.
5. Y. X. J. Wang, D. M. Szantai-Kis and E. J. Petersson, *Org. Biomol. Chem.*, 2016, **14**, 6262-6269.
6. R. F. Wissner, S. Batjargal, C. M. Fadzen and E. J. Petersson, *J. Am. Chem. Soc.*, 2013, **135**, 6529-6540.
7. Y. Huang, J. J. Ferrie, X. Chen, Y. T. Zhang, D. M. Szantai-Kis, D. M. Chenoweth and E. J. Petersson, *Chem. Commun.*, 2016, **52**, 7798-7801.
8. J. M. Goldberg, L. C. Speight, M. W. Fegley and E. J. Petersson, *J. Am. Chem. Soc.*, 2012, **134**, 6088-6091.
9. S. Mukherjee, H. Verma and J. Chatterjee, *Org. Lett.*, 2015, **17**, 3150-3153.
10. S. Batjargal, C. R. Walters and E. J. Petersson, *J. Am. Chem. Soc.*, 2015, **137**, 1734-1737.
11. F. W. Kotch and R. T. Raines, *Proc. Natl. Acad. Sci. USA*, 2006, **103**, 3028-3033.
12. D. Juminaga, S. A. Albaugh and R. F. Steiner, *J. Biol. Chem.*, 1994, **269**, 1660-1667.
13. I. Protasevich, B. Ranjbar, V. Lobachov, A. Makarov, R. Gilli, C. Briand, D. Lafitte and J. Haiech, *Biochemistry*, 1997, **36**, 2017-2024.
14. B. R. Sorensen and M. A. Shea, *Biochemistry*, 1998, **37**, 4244-4253.
15. G. Gibrat, L. Assairi, C. T. Craescu, G. Hui Bon Hoa, D. Loew, B. Lombard, L. Blouquit and M. C. Bellissent-Funel, *Biochim. Biophys. Acta*, 2012, **1824**, 1097-1106.
16. J. S. Zheng, S. Tang, Y. K. Qi, Z. P. Wang and L. Liu, *Nat. Protocols*, 2013, **8**, 2483-2495.
17. W. J. Becktel and J. A. Schellman, *Biopolymers*, 1987, **26**, 1859-1877.
18. Z. E. Reinert, G. A. Lengyel and W. S. Horne, *J. Am. Chem. Soc.*, 2013, **135**, 12528-12531.
19. Y. T. Zhang, R. M. Malamakal and D. M. Chenoweth, *J. Am. Chem. Soc.*, 2015, **137**, 12422-12425.
20. B. Sacca and L. Moroder, *J. Pept. Sci.*, 2002, **8**, 192-204.
21. S. K. Holmgren, L. E. Bretscher, K. M. Taylor and R. T. Raines, *Chem. Biol.*, 1999, **6**, 63-70.
22. J. Engel, H. T. Chen, D. J. Prockop and H. Klump, *Biopolymers*, 1977, **16**, 601-622.
23. R. S. Erdmann and H. Wennemers, *Angew. Chem. Int. Ed.*, 2011, **50**, 6835-6838.
24. N. J. Greenfield, *Nat. Protocols*, 2006, **1**, 2891-2899.
25. K. Mizuno, S. P. Boudko, J. Engel and H. P. Bachinger, *Biophys. J.*, 2010, **98**, 3004-3014.
26. R. S. Erdmann and H. Wennemers, *J. Am. Chem. Soc.*, 2012, **134**, 17117-17124.
